# Changes in the dopaminergic circuitry and adult neurogenesis linked to reinforcement learning in corvids

**DOI:** 10.3389/fnins.2024.1359874

**Published:** 2024-05-14

**Authors:** Pooja Parishar, Madhumita Rajagopalan, Soumya Iyengar

**Affiliations:** National Brain Research Centre, Gurugram, India

**Keywords:** corvids, visual discrimination, reinforcement learning, dopamine, adult neurogenesis, neurite pruning

## Abstract

The caudolateral nidopallium (NCL, an analog of the prefrontal cortex) is known to be involved in learning, memory, and discrimination in corvids (a songbird), whereas the involvement of other brain regions in these phenomena is not well explored. We used house crows (*Corvus splendens*) to explore the neural correlates of learning and decision-making by initially training them on a shape discrimination task followed by immunohistochemistry to study the immediate early gene expression (Arc), a dopaminoceptive neuronal marker (DARPP-32, Dopamine- and cAMP-regulated phosphoprotein, Mr 32 kDa) to understand the involvement of the reward pathway and an immature neuronal marker (DCX, doublecortin) to detect learning-induced changes in adult neurogenesis. We performed neuronal counts and neuronal tracing, followed by morphometric analyses. Our present results have demonstrated that besides NCL, other parts of the caudal nidopallium (NC), avian basal ganglia, and intriguingly, vocal control regions in house crows are involved in visual discrimination. We have also found that training on the visual discrimination task can be correlated with neurite pruning in mature dopaminoceptive neurons and immature DCX-positive neurons in the NC of house crows. Furthermore, there is an increase in the incorporation of new neurons throughout NC and the medial striatum which can also be linked to learning. For the first time, our results demonstrate that a combination of structural changes in mature and immature neurons and adult neurogenesis are linked to learning in corvids.

## 1 Introduction

Both classical conditioning and operant learning depend on topographically organized fronto-striatal pathways composed of thalamo-cortical-basal ganglia loops (Powell, 1992; Chen et al., 2015; Averbeck and Murray, 2020) which receive dopaminergic input. In avian brains, the caudolateral nidopallium is functionally analogous to the mammalian prefrontal cortex (PFC) (Güntürkün, 2005; Nieder, 2017). Besides NCL, the medial striatum is reported to be involved in task acquisition (Watanabe, 2001) and multi-component behavior (Rook et al., 2020) in pigeons. Furthermore, an anterior nidopallial nucleus (NIML, nidopallium intermedium medialis pars laterale) is known to be involved in sequential learning and serial processing in pigeons (Helduser and Güntürkün, 2012; Rook et al., 2021). Its songbird equivalent, LMAN (lateral magnocellular nucleus of the anterior nidopallium), is a part of the anterior forebrain-basal ganglia loop involved in song learning (Brainard and Doupe, 2002).

The family Corvidae is known for its cognitive abilities, akin to those of great apes (Pika et al., 2020). Corvids display remarkable skills in physical cognition (Hunt and Gray, 2004; Bird and Emery, 2009; Taylor et al., 2009; Bogale et al., 2011a,b; Bogale and Sugita, 2014), social cognition (Dally et al., 2006; Mikolasch et al., 2013; Pika et al., 2020), and facial recognition and discrimination (Marzluff et al., 2010; Bogale et al., 2011c). Most of the studies on corvids have focused only on the role of the NCL in learning and decision-making whereas in other songbirds, the role of the song control system (a basal ganglia-thalamocortical loop) in vocalization and vocal learning, an important aspect of cognition, has been studied extensively. However, studies performed on various mammalian species and on pigeons have demonstrated a role for basal ganglia loops in cognition and learning (Helduser and Güntürkün, 2012; Rook et al., 2020, 2021; Averbeck and O’Doherty, 2022). We therefore hypothesized that brain regions connected to the NCL including components of the basal ganglia would be involved in learning and decision-making in corvids. Besides identifying these regions, we were interested in understanding whether we could identify changes in learning-induced plasticity at the structural level in these brain regions. For these experiments, we used the expression of the immediate early gene Arc (activity-regulated cytoskeletal protein) after house crows (*Corvus splendens*) were trained on a shape discrimination task (Bogale and Sugita, 2014) involving reinforcement learning. In rodents, the expression of Arc is increased by high-frequency stimulation (Lyford et al., 1995; Steward et al., 1998), spatial learning (Guzowski et al., 2000), and housing in enriched environments (Pinaud et al., 2001) in various cortical areas and in the hippocampus. Both singing and hearing induces Arc in song control and auditory areas in songbirds (Velho et al., 2005; Wada et al., 2006). During the sensorimotor phase of song learning, Arc was found to be attenuated in juvenile male birds in RA and NIf during the first 3 h of singing and this attenuation rate reduced when the song stabilized, such as with age or testosterone administration (Hayase and Wada, 2018). Besides vocal learning, auditory stimuli also affect Arc expression. In female zebra finches, hearing the songs of males induced Arc expression in auditory areas MLd (nucleus mesencephalicus lateralis, pars dorsalis), CMM (caudomedial mesopallium) and NCM (caudomedial nidopallium) compared to birds which had not heard these songs (Lin et al., 2014). In 1-day old chicks, a 30 min auditory stimulus (filial imprinting) was also found to induce changes in Arc expression in the association auditory areas [IMM (mesopallium intermediomediale), MNM (medio-rostral nidopallium/mesopallium), NDC (nidopallium dorsocaudal), M2 (mesopallium) and L] (Bock et al., 2005). Furthermore, Arc was upregulated in the context of visual imprinting, a form of learning which leads to the formation of long-term memories in chicks. In these experiments, the expression of Arc was found to increase 10 min after an imprinting stimulus in the nuclei of neurons in the visual Wulst. This was followed by an increase in the expression of cytosolic Arc mRNA 20 min after exposure to the imprinting stimulus (Nakamori et al., 2017). In zebra finches, Arc mRNA colocalizes with egr-1 and c-fos upon song exposure in auditory regions (Velho et al., 2005) and in rodents, Arc induction was found to be more sensitive to behavioral task demands than zif-268 and c-fos (Guzowski et al., 2001) suggesting that the expression of Arc is an important target to study learning-induced neural activity.

Furthermore, we wanted to understand whether changes in neural activity associated with learning led to changes in the dopaminergic system in house crows. The dopaminergic system plays a key role in the motivation to learn, reward prediction, and the subjective valuation of reward (Schultz, 2016). For example, infusions of a dopamine D1 receptor antagonist in the pigeon NCL led to a decrease in their performance on a novel attention-based task (Rose et al., 2010). Dopamine release and an increased expression of D1 receptors were also observed in the pigeon NCL linked to working-memory based tasks (Karakuyu et al., 2007; Herold et al., 2012). We, therefore, quantified changes in DARPP-32 positive dopaminoceptive neurons in different parts of the house crow NC after training them on visual discrimination.

Different strategies adopted by the brain for learning include changes in the number of synapses and/or dendritic remodeling in existing neural circuits to facilitate the consolidation of new information (Comeau et al., 2010) and the addition of new neurons to already existing circuits. For example, shifting rats to an enriched environment (Bose et al., 2010), training them on a T-maze task or spatial reversal learning in a parallel alley maze led to layer-specific changes in the dendritic field of pyramidal neurons in the medial prefrontal cortex (mPFC, CG3) and in the orbitofrontal cortex (OFC). These findings suggest that the plasticity induced by different kinds of experience varies in different cortical areas and layers (Comeau et al., 2010). Learning is also known to induce changes in adult neurogenesis (Leuner et al., 2006). In seasonal songbirds such as male canaries which change their songs annually during the breeding season, projections from the pallial region HVC to the motor nucleus RA (Robust nucleus of the arcopallium) are remodeled during adulthood as a result of neurogenesis. Since these neurons are lost after this period, they may be necessary or permissive for learning or producing new songs (Balthazart et al., 2008). New neurons are also incorporated in the caudomedial nidopallium (NCM), an auditory area important for perception and storage of conspecific songs in zebra finches (Pytte et al., 2010), in the NCL and hippocampus of adult house crows (Taufique et al., 2018) and in the striatum of humans (Parent et al., 1995), rodents (Suzuki and Goldman, 2003; Dayer et al., 2005; García-González et al., 2021), and songbirds (zebra finches) (Lipkind et al., 2002), where they mature into medium spiny neurons (MSNs). Although the striatum has been widely studied for its role in goal-oriented learning and decision-making (Balleine et al., 2007), changes in adult neurogenesis induced by these processes in this region have not been reported so far.

We were interested in understanding whether learning and decision-making induced structural changes in mature dopaminoceptive (DARPP-32) and immature Doublecortin-labeled (DCX) neurons (Taufique et al., 2018) in the house crow NC. Furthermore, we decided to study adult neurogenesis in the striatum and NC using DCX as a marker for immature neurons, since these areas were involved in visual discrimination and are known to recruit new neurons during adulthood (Lipkind et al., 2002).

## 2 Materials and methods

A total of 18 adult house crows (*n* = 12 males and 6 females) were used for the shape discrimination experiments. All experimental birds were wild-caught with the permission of the Chief Wild Life Warden, Haryana, and housed in aviaries at the Animal Facility, National Brain Research Centre, Manesar, and experimental protocols were approved by the Institutional Animal Ethics Committee, NBRC. All experimental procedures used for these studies were carried out according to guidelines laid down by the Committee for the Control and Supervision of Experiments on Animals (CCSEA), India, compliant with international standards on animal welfare. At the end of the experiments, tissue from the liver of the house crows was genotyped to determine their sex, using a previously described protocol (Singh et al., 2018).

### 2.1 Behavioral setup

All house crows were weighed prior to starting behavioral training. Experimental crows were housed in an outdoor aviary with natural day and light conditions. Two days prior to training on the visual discrimination experiments, they were transferred to a cage (dimensions: 30″ × 21″ × 34″) in a separate room (visually and auditorily isolated from other crows) also maintained in natural light and dark conditions for habituation. On the second day of this period, they were food-deprived from 4 pm in the evening until the beginning of the pre-training period which started at 9 a.m. the next day. Birds were given *ad libitum* access to water but the amount of food given post-behavioral training depended on the appetite of each bird, since some of the birds did not participate in the training paradigm if they were fully satiated the previous evening. For the food reward, we used pieces of dried shrimp, chicken sausage, or vitamin-fortified white bread depending on the crows’ preference. A total of two blocks of the shape discrimination task comprising 12 trials each were conducted every day with a gap of 4–5 h, 5 days a week. Over the weekend, experimental birds were transferred back to their aviaries where they were in visual and auditory contact with other crows and provided eggs, bread, dog food (Pedigree), and water *ad libitum*. There was no reduction in the body weight (∼250 gm) of any of the house crows at the end of training on the behavioral paradigm.

#### 2.1.1 Pre-training

All experimental crows were initially trained to retrieve food rewards placed on a platform in the cage to habituate them to the apparatus used for the behavioral experiments. After birds learned to retrieve food from the platform, food rewards were partially hidden by two three-dimensional plastic blocks with the same surface area (a triangle and a circle). With further training, birds learned to retrieve food from under the blocks, after which they were trained for the visual discrimination experiment.

#### 2.1.2 Training

During the training trials, the experimenter placed the two blocks on the platform in the cage, closed the cage door, and hid behind a screen outside the visual range of the crows, which marked the beginning of a trial (Supplementary Figure 1A). The positions of the shapes were randomized in the cage to prevent birds from associating the reward with spatial locations. A trial was considered complete when the crow hopped down from its perch, knocked over a shape to retrieve the food reward, and returned to the perch or at the end of 2 min, whichever was sooner. If crows did not attempt to retrieve food rewards in three consecutive trials, the block of experiments was stopped and if crows started attacking the camera, that trial was stopped.

The four groups used for our experiments are as follows:

1. Trained group (*n* = 4; males = 3, females = 1): The food reward was placed only under the triangular block. Birds were trained to retrieve food until their success rate on the task reached 80% or above in a block. After two consecutive blocks during which crows’ performance remained at 80% or above, they were considered fully trained.
2. Undertrained group (*n* = 6; males = 5, females = 1): The food reward was placed only under the triangular block, similar to conditions for the Trained group. For this group, the experiment ended when they achieved a success rate of ∼ 40 to 60%. The purpose of including the Undertrained group was to detect and compare changes in areas which were activated while they were learning.
3. No-Association group (*n* = 4; males = 2, females = 2). The food reward was randomly presented under either shape and the experiment ended after the birds in this group received two blocks of training. In each session, each shape was rewarded 50% of the times. This group acted as a negative control since these birds cannot associate the reward with either of the shapes.

Birds from Groups 1 to 3 were kept in the dark for 1 h to minimize neural activity resulting from other visual stimuli at the end of a block of trials.

4. Baseline group (*n* = 4; males = 2, females = 2): Crows were placed in the cage for behavioral assessment and exposed to the shapes placed on the platform for 6 h on 2 consecutive days without food deprivation. On the third day, they were exposed to the behavioral setup for 30 min after which they were placed in the dark prior to ending the experiment. In this group, birds were exposed to the apparatus for a long period in order to saturate them with the visual stimuli elicited by the behavioral setup. As a result, we did not expect to observe neural activity in their brains due to the novelty of the visual stimuli.

At the end of the last block of trials, birds were kept in the dark for 90 min and anesthetized with an overdose of ketamine (30 mg/kg) and xylazine (2 mg/kg). This was followed by intracardial perfusion with 0.01 M phosphate-buffered saline (PBS), followed by 4% paraformaldehyde (PFA). Brains were removed and post-fixed with 4% PFA (for 1 week at 4°C), after which they were cryopreserved in 30% sucrose and cryosectioned serially at 50 μm in the coronal plane. We obtained six series from each hemisphere. The first series of sections from both hemispheres were stained with Nissl’s stain for neuroanatomical localization of different regions and the rest of the series were processed for immunohistochemistry.

### 2.2 Western blotting

Immunoblotting was performed to check the validity of the DCX antibody [sc-271390, Anti-Doublecortin Antibody (E-6); RRID:AB_10610966, Santa Cruz Biotechnology] using samples of tissue from the anterior striatum of a house crow (*n* = 1, Female). The tissue was homogenized in SDS lysis buffer and sonicated with 25 pulses of 1 s each (thrice with an interval of 5 s), followed by centrifugation at 12000 rpm at 4°C. The supernatant was collected and protein was estimated using the bicinchoninic acid protein estimation method (BCA, B9643, Sigma-Aldrich). We separated a protein sample (80 μg) on an SDS-PAGE gel (11% Acrylamide-Bisacrylamide gel concentration). The resolved proteins were transferred to a nitrocellulose membrane which was blocked with 5% BSA (Bovine serum albumin) for 2 h. This was followed by incubation in the primary antibody solution (1:1000, anti-DCX) for 13–16 h at 4°C. The primary antibody was rinsed using six washes of TBST (Tris borate saline with 0.1% Tween 20, 10 min each) followed by incubation in a secondary antibody solution (1:3000, peroxidase labeled anti-mouse, PI-2000, Vector laboratories). This was followed by rinsing in TBST, after which the blot was developed with the ECL chemiluminescent reagent (WBKLS0500, Immobilon Western Chemiluminescent HRP Substrate, MERCK, USA). We obtained an intense band at ∼40 kD and a very faint one at ∼32 kD (Supplementary Figure 1B) when a western blot was performed on lysate from a house crow brain using an anti-DCX antibody (sc-271390, Santa Cruz Biotechnology). Bands were obtained at similar molecular weights in western blots performed on mouse brain tissue provided by the manufacturer.

### 2.3 Immunohistochemistry

#### 2.3.1 Arc, DCX, TH, and DARPP-32

Coronal serial sections (50 μm thick) from the right hemisphere were labeled using immunohistochemistry for Arc (Cat# ab85656, Abcam, RRID:AB_1924788), Tyrosine hydroxylase (Cat# MAB318, Merck-Millipore, RRID:AB_2201528) or DARPP 32 (Cat# ab-40801, Abcam; RRID:AB_731843). Sections from the left hemisphere were used for immunohistochemistry against DCX. The antibodies used to detect Arc, TH, and DARPP-32 have been previously used in songbirds (Gilbert and Soderstrom, 2013; Sen et al., 2019). Sections were incubated in an antigen unmasking solution (H-3300, Vector laboratories) for 30 min at 80°C in a water bath, followed by rinsing with 0.01 M PBS (only for DCX). After rinsing with 0.01 M PBS, sections were incubated in 1–3% H_2_O_2_ in 0.3% Triton-X 100 for 30 min to quench endogenous peroxidase activity. After quenching, sections were incubated in a blocking solution [5% normal goat serum, NGS, for Arc; 5% NGS and 2% Bovine serum albumin, BSA, for DARPP-32 and 5% Normal horse serum, NHS, and 2% BSA for TH and DCX; S-1000 (NGS) and S-2000 (NHS), Vector Laboratories, Burlingame, CA] for 2 h. This was followed by incubation in a solution containing the primary antibody [Arc; 1:1000; (incubation for 38–40 h at 4°C); DCX; 1:500 (incubation for 16–20 h at 4°C); TH; 1:200 (incubation for 38–40 h at 4°C) or DARPP-32; 1:500 (incubation for 16–20 h at 4°C); made in blocking buffer containing 0.3% triton-X PBS]. This was followed by rinsing the sections in PBS and incubating them in a secondary antibody solution (biotinylated anti-rabbit for Arc and DARPP-32 and biotinylated anti-mouse for DCX and TH; 1:200; BA-1000, BA-2000 Vector Laboratories) for 2 h. After rinsing in PBS, sections were incubated in a solution containing avidin-biotin complex (ABC reagent; PK-6100, Vectastain Elite ABC HRP kit, Vector Laboratories; 1:50) for 2 h. Sections were again rinsed in PBS and then developed in a solution containing the chromogen [Nova Red peroxidase (HRP) substrate kit (SK-4800, Vector Laboratories)] according to the manufacturer’s instructions. Finally, sections were rinsed with Milli Q and mounted on gelatin-coated slides, after which they were air-dried overnight and cover-slipped with DPX.

### 2.4 Double immunofluorescence

#### 2.4.1 Arc and DARPP-32

In order to study whether DARPP-32-positive neurons were activated as a result of the visual discrimination task, sections from the crow brain from different groups were double-labeled for DARPP-32 and Arc. Coronal serial sections at the level of the caudal nidopallium were used for these experiments. A different antibody was used for Arc (Cat# NBP1-56929, Novus Biologicals, RRID:AB_11010941) rather than the one used for single label (see above) due to technical problems associated with immunofluorescence. Before testing for double-immunofluorescence, we tested this antibody singly and found no difference in the expression patterns of the two Arc antibodies. For double immunofluorescence, the basic steps of quenching and permeabilization were performed as described above. Sections were then blocked with 5% NGS for 2 h, followed by incubation in the primary antibody solution (1:500, anti-DARPP-32 made in rabbit 5% NGS) for 38–40 h at 4°C. Sections were rinsed and incubated in the secondary antibody solution (1:200, anti-rabbit; Alexa-594, A11012, ThermoFisher Scientific, USA) for 4–5 h at room temperature. Later, they were washed with PBS and blocked with 5% NGS and 2% BSA for 2 h, followed by incubation in the anti-Arc primary antibody (1:200, anti-Arc antibody made in rabbit in blocking buffer with 0.3% triton-X) for 38–40 h at 4°C. Sections were washed with PBS and incubated in the secondary antibody solution (1:200, Alexa-488 anti-rabbit; A11008, ThermoFisher Scientific, USA) for 4–5 h followed by three washes in PBS. Sections were then transferred onto gelatin-coated slides and cover-slipped with antifade DAPI mounting media (H-1800, Vector laboratories).

#### 2.4.2 Arc and DCX

To study whether DCX-positive neurons were activated following the visual discrimination task, serial coronal sections of the house crow brain at the level of MSt, Area X, and caudal nidopallium were double-labeled with Arc and DCX. Sections were incubated in the antigen unmasking solution for 30 min at 80°C in a water bath followed by rinsing with PBS. The steps for quenching and permeabilization were performed next, as described above. Sections were then blocked with 5% NGS for 2 h, followed by incubation in the primary antibody cocktail (1:200, anti-DCX made in mouse and 1:200, anti-Arc) for 38–40 h at 4°C. Sections were rinsed and incubated in the secondary antibody cocktail (1:200, anti-mouse; Alexa-594, Cat. # A11005 and 1:200, Alexa-488 anti-rabbit, Cat. # A11008; ThermoFisher Scientific, USA) for 4–5 h at room temperature. After this step, they were washed with PBS, transferred onto gelatin-coated slides, and mounted with antifade DAPI mounting medium (H-1800, Vector laboratories).

### 2.5 Quantitative analysis of tyrosine hydroxylase positive profiles

Sections at the level of NCL were stained with TH and demarcated into various subdivisions according to an earlier report from our lab (Sen et al., 2019). In each subdivision, the area of TH-positive profiles was determined using ImageJ (version: 1.52). The contours of different subdivisions in NC (dNC, iNC, mNCL, lNCL, and vNC) determined using TH staining patterns were used to determine the boundaries of NC subdivisions in Arc and DARPP-32-stained sections. We converted the images to 8 bits and thresholded them based on staining in lNCL, followed by quantifying the stained areas (NC subdivisions). We normalized the % area fraction of the five NC subdivisions with a positive control, that is, a band of TH-positive profiles located between LAD (Lamina arcopallialis dorsalis) and AId.

### 2.6 Neuron counts

A contour was drawn around the area of interest in serial sections using the Stereoinvestigator software (Microbrightfield, Williston, VT) linked to an Olympus microscope (BX-51). The optical fractionator method (Olesen et al., 2017) was used to count Arc-, DCX-, and DARPP-32-positive cells, which is a stereological counting method and performs a Systematic Uniformly Random Sampling (SURS) by dividing the area of interest into a grid. Cell counts are performed by selecting the sampling fraction which is a proportion of the area (bin) in which counting is performed. Intensely stained Arc / DCX / DARPP-32-positive neurons were counted and considered for analysis. The Arc-positive neurons were counted at 100X using a sampling grid of size 70 μm × 70 μm with an area sampling fraction (asf) of 5 or 10 based on the size of the area and a dissector height of 9 μm with 2 μm guard zones. The DCX and DARPP-32-positive neurons were counted at 40X using a grid size of 175 μm × 175 μm with asf of 50, 30, or 20 and dissector height of 10 μm with 2 μm guard zones. Additionally, we counted the three different neuronal populations stained for DCX, namely, multipolar, fusiform, and spherical. For all fractionator counts, every 6th section was sampled. An estimated count using mean section thickness for Arc, DCX, and DARPP-32 was used to compare the neuronal population across various training groups. To accommodate size differences across brain regions between different birds, we normalized the estimated counts by the number of bins from each area, which is an indicator of the size of the area.

### 2.7 Fluorescence imaging and neuron counting

Fluorescence imaging for sections stained for Arc and DARPP-32 were performed using an Apotome microscope (Carl Zeiss1, AxioImager.z1). We imaged z-stacks (magnification: 40X for counting and 63X for imaging) at an interval of 1.5 μm from different subdivisions of NC from all crows used in our experiments on visual discrimination. Manual neuronal counts for Arc and DARPP-32 double-positive neurons were performed from these images using ImageJ software.

### 2.8 Morphometric analysis of DARPP-32 and DCX multipolar neurons

We traced 15 DARPP or DCX positive neurons from each subdivision of the caudal nidopallium from each bird using the Neurolucida software (version:11, MBF Bioscience, USA) linked to a microscope (Olympus BX51) at a magnification of 100X. After a careful visual inspection, only those neurons which possessed intact processes were selected for tracing. Since we could not discriminate between the axons and dendrites of neurons based on staining for DARPP-32 or DCX, we have considered all processes as neurites for our study. Traces were exported to the Neurolucida Explorer software and subjected to three morphometric analyses, including (1) Neuron summary analysis for overall changes in the neurite branching and length, (2) Convex hull analysis to quantify changes in the neurite field, and (3) Sholl analysis for quantifying neuronal complexity, wherein the traced neuronal soma was placed at the center of a set of concentric circles at a fixed distance (10 μm). The number of intersections that the neurite tree made with these circles was counted and for each Sholl radius, the total neurite length was analyzed. Statistical analysis was performed on changes observed between the radii from 20 to 70 μm. However, data from changes within the 10 μm Sholl radius were not analyzed since we observed an increase in the size of somata of DARRP-32 and DCX positive neurons in Trained, Undertrained, and No-Association birds compared to Baseline controls and the inclusion of these data might introduce a false estimation of the analyzed parameters. The initial Sholl radius was reduced to 8 μm for DCX neurons from the striatum due to the smaller size of their somata.

Similarly, five Arc and DARPP-32/DCX double-positive neurons each from MSt, mNCL and lNCL regions were traced using the Neurolucida software (version 2020.1.3; MBF Bioscience, USA) linked to a fluorescence microscope (Olympus BX53). The three-dimensional tracings of these neurons were analyzed in the same manner as described above for individually labeled neurons using the Neurolucida Explorer software.

### 2.9 Statistical analysis

The R (version 4.1.1) software was used to perform statistical tests and visualize the data. Data pertaining to counts (Arc, DARPP-32, DCX and TH) were tested first for normality and homoscedasticity using the Shapiro-Wilk and Levene tests. If the data did not pass these tests, a Kruskal-Wallis test was performed otherwise a one-way analysis of variance (ANOVA) was performed for judging statistical significance. For pairwise multiple comparisons, either Tukey’s (multcomp library; adjusted *P*-values are generated by the single-step method) or Dunn’s *post-hoc* test (FSA library using the Holm method for *P*-value adjustment) were performed.

In order to check the interaction effects between area and experimental conditions for neuronal morphometry data, we performed a two-way ANOVA. For non-normal data, an aligned rank transform was performed, followed by two-way ANOVA (Wobbrock et al., 2011). For group comparisons, data was initially checked for normality and equal variance as described above followed by either one-way ANOVA or the Kruskal-Wallis test and *post-hoc* tests as described above. For comparisons involving only two groups in some cases, a Welch’s two-tailed t-test or Wilcoxon rank sum test was performed. Details of the statistical analyses for neuronal structural changes (F and/or χ^2^ values, degrees of freedom and exact *P*-values) are provided in separate Supplementary Tables 1–18 due to the large number of comparisons.

## 3 Results

### 3.1 Activation of house crow brain regions associated with visual discrimination

**FIGURE 1:**
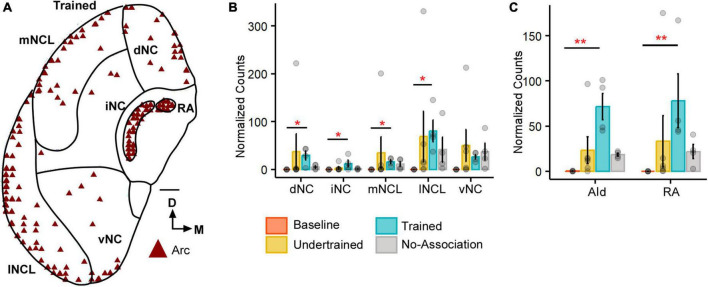
The expression of Arc in the caudal nidopallium (NC) and arcopallium across different sets of experimental birds. **(A)** A coronal schematic of the posterior part of the house crow telencephalon demonstrating label for Arc in the caudal nidopallium and areas of the arcopallium, that is, AId and RA, from one of the Trained birds. Scale bar, 1 mm. A comparison of **(B)** darkly stained Arc-positive neuronal population across different subdivisions of the caudal nidopallium revealed that the highest levels of activity were observed in lNCL (mean ± SEM). The number of Arc-positive neurons was the highest in the Trained category followed by that in the No-Association group and the Baseline group had negligible numbers of Arc-labeled neurons. Levels of neural activity were highly variable in the Undertrained group in all divisions of the caudal nidopallium. A comparison of **(C)** Arc counts across different behavioral groups in AId and RA. The highest number of Arc-labeled neurons were present in AId and RA of the Trained group, followed by that in the No-Association and Undertrained group. The lowest number of Arc-positive neurons were present in the Baseline group in both AId and RA. Asterisks indicate significant differences; *P < 0.05; **P < 0.01. *N* = 4 data points for Baseline, Trained and No-Association; *N* = 6 for Undertrained.

Besides NC (Figure 1A), areas including LMAN, Area X, RA, and AId, generally associated with song control (Brainard and Doupe, 2002) expressed the Arc protein in different groups of house crows (Supplementary Figures 2A, B, 3C). Intensely stained neurons were also present in components of the basal ganglia including LSt, GP (Supplementary Figures 3A, 4A) and MSt, and the midbrain dopaminergic areas, VTA and SN (Supplementary Figure 4C; see Supplementary Figure 3B for a negative control devoid of label at the level of GP and LSt).

Details of the statistical analyses for neuron morphometry data are provided in the Supplementary Tables.

#### 3.1.1 Caudal telencephalon

All five subdivisions of the caudal nidopallium (dNC, iNC, lNCL, mNCL, and vNC) in house crows (Sen et al., 2019; Figure 1A) contained Arc-positive neurons following performance on the visual discrimination task. There were significantly fewer Arc-positive neurons in Baseline controls vs. those in Trained birds in dNC, iNC, lNCL, and mNCL [Figure 1B; (dNC: Kruskal-Wallis test; χ^2^ = 8.801, *P* = 0.032, Dunn’s *post-hoc* test; *P*_(Bl vs. Tr)_ = 0.021); (iNC: χ^2^ = 8.7548, *P* = 0.033, *P*_(Bl vs. Tr)_ = 0.022); (lNCL: χ^2^ = 9.8047, *P* = 0.02, *P*_(Bl vs. Tr)_ = 0.012); mNCL: χ^2^ = 9.8673, *P* = 0.019, *P*_(Bl vs. Tr)_ = 0.0156]. We also observed that the number of Arc-labeled neurons was highly variable across NC in Undertrained house crows (Figure 1B).

Besides activation in NC, there were significantly more Arc-positive neurons in the arcopallial region AId of Trained birds compared to those in Baseline controls (*P* < 0.01) (Figure 1C; Kruskal-Wallis test, χ^2^ = 11.987, *P* = 0.007, Dunn’s *post-hoc* test; *P*_(Bl vs. Tr)_ = 0.005). Furthermore, there were significantly more Arc-positive neurons in RA of Trained vs. Baseline birds (Figure 1C; Kruskal-Wallis test, χ^2^ = 12.282, *P* = 0.006, Dunn’s *post-hoc* test; *P*_(Bl vs. Tr)_ = 0.0032), which plays an important role in vocalization and breathing (Schmidt and Martin Wild, 2014).

#### 3.1.2 Regions adjacent to the anterior commissure

There were significant differences in the number of Arc-labeled neurons at the level of GP and LSt in Undertrained, and No-Association birds vs. those in Baseline controls (Supplementary Figures 4A, B; GP: Kruskal-Wallis test, χ^2^ = 9.633, *P* = 0.022, Dunn’s *post-hoc* test; *P*_(Bl vs. Na)_ = 0.026; LSt: χ^2^ = 9.043, *P* = 0.029; *P*_(Bl vs. Ut)_ = 0.029). We did not find significant differences in Arc counts across Trained, No-Association and the Undertrained groups. Furthermore, there were very few Arc-positive neurons in these regions in the Baseline group. A non-significant difference was observed when other categories were compared with the Baseline group possibly due to behavioral variability and the small sample size used for this study. Similar results were also obtained for the midbrain regions VTA and SN (Supplementary Figures 4C, D; SN: Kruskal-Wallis test, χ^2^ = 8.33, *P* = 0.039, Dunn’s *post-hoc* test; *P*_(Bl vs. Tr)_ = 0.0397), that is, a greater number of Arc-labeled neurons in SN and VTA in the Trained, No-Association and Undertrained groups vs. the Baseline controls. However, these differences did not achieve statistical significance.

#### 3.1.3 Anterior forebrain

**FIGURE 2:**
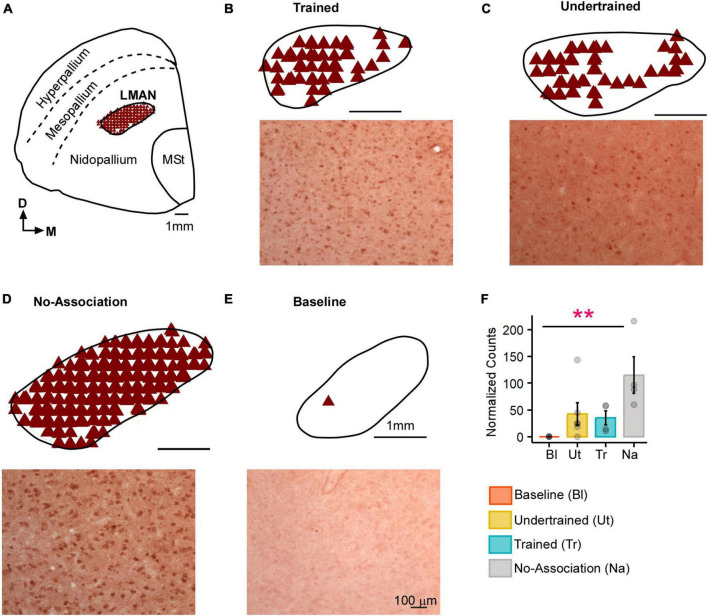
Task-related changes in Arc expression in LMAN. **(A)** A schematic of the anterior forebrain at the level of LMAN showing Arc expression from a house crow from the No-Association group. Counts of Arc-positive neurons are shown in the schematics at the top and high magnification images of LMAN are shown below in each panel for **(B)** Trained, **(C)** Undertrained, **(D)** No-Association, and **(E)** Baseline groups of experimental birds. The highest expression of Arc was present in the No-Association group **(D)**, whereas there was minimal expression in the **(E)** Baseline group. **(F)** Comparisons of normalized Arc counts (mean ± SEM) in LMAN demonstrated that levels of neural activity were significantly higher in the No-Association group vs. the Baseline group. Scale bar, 1 mm for the section and LMAN schematic and 100 μm for Arc-labeled magnified images. Asterisks indicate significant differences; **P < 0.01. *N* = 4 data points for Baseline, Trained and No-Association birds; *N* = 6 for Undertrained.

There was an increase in neural activity in LMAN of No-Association birds compared to that in Baseline, Trained and Undertrained groups. However, a *post hoc* analysis revealed that these differences were statistically significant only for comparisons of Arc-positive neuronal counts between Baseline and No-Association birds (Figures 2A–F; Kruskal-Wallis test, χ^2^ = 11.01, *P* = 0.012, Dunn’s *post-hoc* test; *P*_(Bl vs. Tr)_ = 0.005).

**FIGURE 3:**
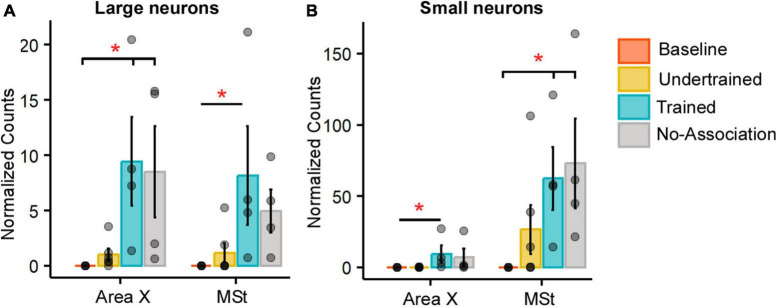
Quantification of Arc-positive neurons in Area X and MSt following the visual discrimination task. Bar graphs representing normalized counts (mean ± SEM) for Arc positive **(A)** large neurons **(B)** small neurons in Area X and MSt across various behavioral groups. The Baseline group has the lowest number of Arc-positive neurons in both Area X and MSt regardless of the cell type, followed by the Undertrained group, whereas there were a large number of Arc-labeled neurons in the Trained and No-Association groups. Asterisks indicate significant differences; *P < 0.05. *N* = 4 data points for Baseline, Trained and No-Association; *N* = 6 for Undertrained birds.

In the striatum at this level, Arc-labeled neurons were observed in Area X and the surrounding MSt in both large parvalbumin-positive (interneurons/pallidal neurons) or smaller parvalbumin-negative neurons (Supplementary Figures 5A–E). When the number of Arc-positive neurons of different sizes in Area X and MSt were compared across different experimental groups, the largest number of these neurons was observed in Trained and No-Association birds, whereas the smallest number were present in Baseline controls [Figures 3A, B; (Area X:Large neurons: Kruskal-Wallis test, χ^2^ = 12.136, *P* = 0.0069, Dunn’s *post-hoc* test; *P*_(Bl vs. Tr)_ = 0.012; *P*_(Bl vs. Na)_ = 0.03; Area X: Small neurons: χ^2^ = 11.878, *P* = 0.008; *P*_(Bl vs. Tr)_ = 0.027); (MSt: Large neurons: χ^2^ = 10.725, *P* = 0.013; *P*_(Bl vs. Tr)_ = 0.041; MSt; Small neurons: χ^2^ = 10.253, *P* = 0.014; *P*_(Bl vs. Tr)_ = 0.037, *P*_(Bl vs. Na)_ = 0.024)].

### 3.2 Circuitry involved in motivation and reward

#### 3.2.1 Tyrosine hydroxylase (TH-positive) fibers

Different subdivisions of NC were demarcated in all house crows, based on TH expression (a marker for catecholaminergic synthesis; Supplementary Figures 6A–C) as previously described (Sen et al., 2019; Supplementary Figure 6A). The only difference across various groups of birds was a greater density of TH-positive fibers in Baseline birds vs. that in Undertrained birds in lNCL (Supplementary Figure 6B; ANOVA, *P* = 0.0126, *F*_(3,14)_ = 14.271; Tukey’s *post-hoc* test, *P* = 0.0084).

#### 3.2.2 Dopaminoceptive (DARPP-32-positive) neurons in NC and the medial striatum

We quantified dopaminoceptive neurons in MSt, Area X (Supplementary Figure 7A) and NC of all experimental birds (Supplementary Figure 7C). The boundaries of Area X were decided based on staining patterns of DARPP-32 and Nissl. Whereas the density of DARPP-32 neurons was lower in Area X compared to that in MSt (Supplementary Figure 7B), there were no significant differences in the number of dopaminoceptive neurons in the striatum or NC (Supplementary Figure 7D) in various experimental groups.

##### 3.2.2.1 Learning-induced changes in dopaminoceptive neurons

There was a statistically significant interaction between the subdivision of NC and experimental condition for the number of nodes (*P* = 7.2935e-10), neurite length (*P* < 2.22e-16), and neurite surface area (*P* < 2.22e-16). The complexity of DARPP-32-labeled neurons (based on neurite length, nodes and number of intersections from a Sholl analysis; Supplementary Figures 8A–J and Supplementary Table 1) increased in all pallial subdivisions in Trained birds vs. those in other groups. The neurite length and number of nodes of DARPP-32-labeled neurons located in mNCL and lNCL of the No-Association group increased significantly compared to those in Baseline birds (Supplementary Figures 8D–J and Supplementary Table 1) and only at one Sholl radius, that is, at 50 μm, we found a significantly greater number of intersections compared to the Undertrained category in lNCL (Supplementary Figure 8I and Supplementary Table 1). The Trained group demonstrated significantly greater changes in neurite length, number of nodes and number of intersections in mNCL and lNCL compared with that in the Undertrained and No-Association groups (Supplementary Figures 8D, E, G–J and Supplementary Table 1). The three-dimensional surface area of DARPP-32-labeled neurons and their projections was significantly greater in Trained, Undertrained and No-Association groups vs. that in Baseline controls (Supplementary Figure 8F and Supplementary Table 1). We also found that amongst these three experimental groups, the neurite field of DARPP-32-positive neurons was significantly greater in all divisions of NC of the Trained group compared to that in others. Lastly, there was an increase in the size of neuronal somata in the Trained, No-Association and Undertrained groups compared to that in the Baseline group (Supplementary Table 1).

The other NC subdivisions (dNC, iNC and vNC) also demonstrated changes in the complexity of dopaminoceptive neurons. The number of intersections and neurite length increased significantly in dNC and iNC in Trained, No-Association and Undertrained birds vs. Baseline controls and in Trained vs. Undertrained crows (Supplementary Figures 9A, B, D, E and Supplementary Table 2). However, these parameters were similar in Trained and No-Association birds except at one Sholl radius (60 μm) in dNC (Supplementary Figure 9A and Supplementary Table 2). At the 20 μm radius in iNC, differences were also observed between the No-Association and Undertrained groups (Supplementary Figures 9B, E and Supplementary Table 2). In vNC, the differences in the neurite length and number of intersections for DARPP-32-positive neurons were only observed between Trained and Undertrained vs. Baseline and Trained vs. No-Association and Undertrained groups (Supplementary Figures 9C, F and Supplementary Table 2) Overall, learning appeared to lead to an increase in the number and length of neurites of dopaminoceptive neurons in all divisions of the house crow NC.

##### 3.2.2.2 Counts and structural complexity of active DARPP-32-labeled neurons in NC

**FIGURE 4:**
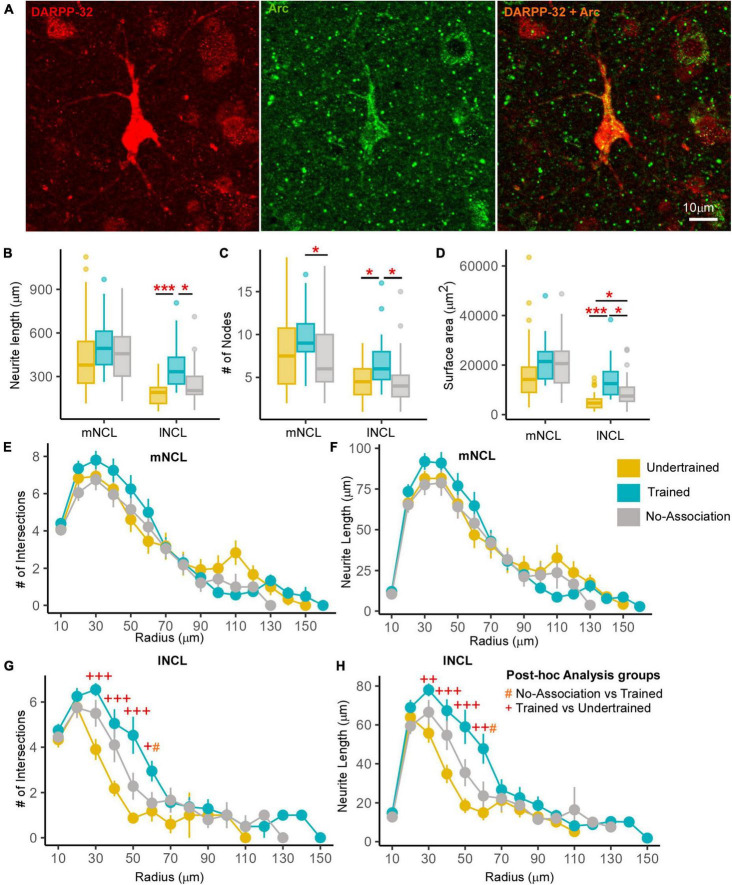
Differences in the structure of neurons double-labeled for Arc and DARPP-32 in NCL. **(A)** High power image of a DARPP-32-labeled neuron (red) from the lNCL region which was also labeled for Arc (green). The third image in this panel shows the co-localization of DARPP-32 and Arc in this neuron. Scale bar: 10 μm. Box and whisker plots demonstrate a statistically significant increase in **(B)** neurite length and the **(C)** number of nodes in Trained birds compared to that in the No-Association and Undertrained categories in lNCL and in Trained vs. No-Association birds in mNCL. **(D)** An increase in the surface area of neurites was seen in lNCL of Trained birds vs. that in Undertrained and No-Association birds. A Sholl analysis demonstrated non-significant increases in the **(E)** number of intersections and **(F)** neurite length (mean ± SEM) in mNCL of the Trained group compared to that in others. A significant difference was observed in **(G)** the number of intersections and **(H)** neurite length in Trained vs. Undertrained birds and No-Association vs. Trained birds in lNCL. */^#^/^+^*P* < 0.05; ^++^*P* < 0.01; ***/^+++^*P* < 0.001. *N* = 20 data points for Trained and No-Association; *N* = 30 for Undertrained, wherein *, #, and + represent significant differences.

Neurons double-labeled for Arc and DARPP-32 (active dopaminoceptive neurons) were present in NC in all groups of experimental birds except Baseline controls, which were not analyzed further (Figure 4A). Active (Arc-positive) dopaminoceptive neurons were reconstructed only in mNCL and lNCL, since the greatest changes in the number of Arc-positive neurons and in the structural complexity of DARPP-32-labeled neurons occurred in these subdivisions. A One-way ANOVA revealed that the number of active dopaminoceptive neurons was significantly greater in Undertrained vs. Trained and No-Association birds only in lNCL (Supplementary Figures 10A, B; ANOVA, *P* = 0.0138; *F*_(2,11)_; Tukey’s *post hoc*, Ut vs. Na: *P* = 0.0219; Ut vs. Tr: *P* = 0.0414). A non-significant increase in this measure was observed in Undertrained birds vs. other experimental groups of house crows in all other subdivisions of NC.

##### 3.2.2.3 Morphometric analysis of active and inactive dopaminoceptive neurons

Active dopaminoceptive neurons were observed throughout mNCL and lNCL (Figure 4A). A One-Way ANOVA revealed that neurite length of these neurons was significantly greater in lNCL of Trained vs. Undertrained and No-Association birds (Figure 4B; *P* < 0.05; Supplementary Table 3). The number of nodes increased significantly with training in both mNCL and lNCL (Figure 4C; *P* < 0.05; Supplementary Table 3). Furthermore, the three-dimensional surface area of these neurons increased significantly in lNCL in Trained vs. Undertrained and No-Association groups and in No-Association vs. Undertrained crows (Figure 4D; *P* < 0.05 and *P* < 0.001; Supplementary Table 3).

Based on Sholl analysis, there were no significant differences in the complexity of active dopaminoceptive neurons in mNCL, except for a non-significant increase in the number of intersections and neurite length in Trained birds vs. other groups (Figures 4E, F and Supplementary Table 4). Both the number of intersections and neurite length were significantly higher in lNCL in Trained vs. Undertrained and No-Association birds between Sholl radii of 30–60 μm (*P* < 0.05, *P* < 0.01, and *P* < 0.001; Figures 4G, H and Supplementary Table 4). Taken together, our findings suggest that active dopaminoceptive neurons in lNCL increased in complexity as a result of training.

**FIGURE 5:**
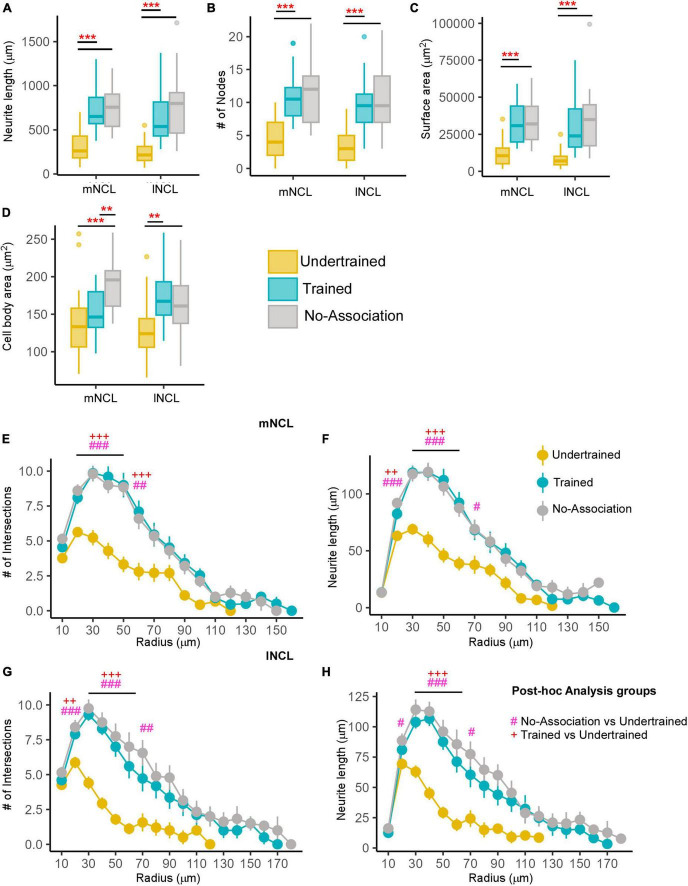
Differences in the morphology of inactive DARPP-32 (Arc-negative) neurons in NCL. Box and whisker plots demonstrating changes in **(A)** neurite length, **(B)** number of nodes, **(C)** neurite field area, and **(D)** cell body area in mNCL and lNCL. In both regions, inactive DARPP-32 neurons demonstrated greater complexity and cell body expansion in the Trained and No-Association groups compared to that in Undertrained birds. A Sholl analysis in mNCL demonstrated an increase in **(E)** the number of intersections and **(F)** neurite length. Similar results were obtained for the **(G)** number of intersections and **(H)** neurite length in lNCL. ^#^*P* < 0.05; **/^##^/^++^*P* < 0.01; ***/^###/+++^*P* < 0.001. *N* = 20 data points for Trained and No-Association; *N* = 30 for Undertrained, wherein *, #, and + represent significant differences.

The complexity of inactive (Arc-negative) DARPP-labeled neurons in mNCL and lNCL (based on neurite length, number of nodes, and surface area) was significantly greater in Trained and No-Association birds compared to those in Undertrained birds (Figures 5A–C; *P* < 0.001; Supplementary Table 5). Their soma area was also significantly greater in Trained and No-Association birds vs. that in Undertrained birds (Figure 5D; *P* < 0.01 and *P* < 0.001; Supplementary Table 5). Similarly, the number of intersections and neurite length increased significantly in both regions between Sholl radii 20–70 μm in Trained and No-Association crows vs. Undertrained birds (Figures 5E–H and Supplementary Table 6).

##### 3.2.2.4 Differences between active and inactive dopaminoceptive neurons

All measures of complexity of neurites for active DARPP-32-positive neurons in lNCL were significantly lower than the inactive dopaminoceptive neurons in Trained and No-Association birds (Supplementary Figures 11A–D; *P* < 0.05, *P* < 0.01, and *P* < 0.001; Supplementary Table 7). However, the soma area of active and inactive dopaminoceptive neurons did not vary across groups (Supplementary Figure 11E and Supplementary Table 7).

As for mNCL, active DARPP-32-labeled neurons were less complex, with lower neurite length, number of branches and neurite surface area compared to inactive ones in No-Association birds (Supplementary Figures 11F–I; *P* < 0.01 and *P* < 0.001; Supplementary Table 7). Whereas active neurons of Trained crows demonstrated similar trends, significant differences were only observed for neurite length and neurite field area. In contrast to lNCL, active dopaminoceptive neurons in mNCL of Undertrained birds demonstrated a significant increase in branching and soma area vs. inactive ones (Supplementary Figures 11F–J; *P* < 0.05 and *P* < 0.001; Supplementary Table 7).

### 3.3 Changes in adult neurogenesis: Immature (DCX-labeled) neurons in house crows

Based on their morphology, DCX-positive neurons in house crows could be categorized as (i) spherical neurons which were devoid of processes, (ii) spindle-shaped unipolar or bipolar fusiform neurons, and (iii) multipolar neurons whose somata were rounded (Brown et al., 2003; Balthazart et al., 2008; Taufique et al., 2018) or triangular (Mehlhorn et al., 2022; Supplementary Figures 12A–C; see Supplementary Figure 12G for the negative control).

#### 3.3.1 Stereological counts of DCX-positive neurons

##### 3.3.1.1 Medial and lateral striatum

**FIGURE 6:**
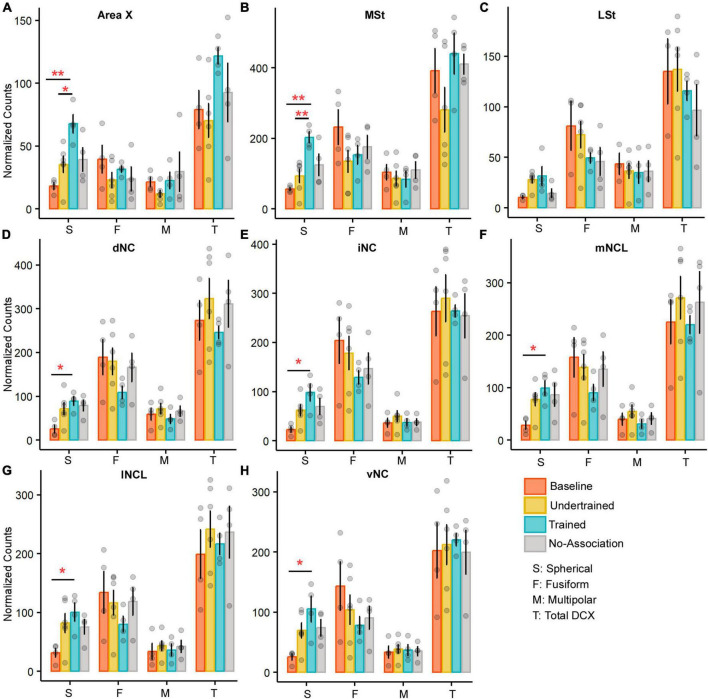
A comparison of the number of various types of DCX neurons in the medial striatum and NC of the four experimental groups of house crows. Bar graphs (mean ± SEM) were plotted to compare the number of DCX-positive neurons across the four experimental groups in **(A)** Area X and **(B)** MSt. The number of spherical DCX-positive neurons was significantly higher in the Trained group vs. Baseline and Undertrained groups in both areas, whereas there were no significant differences in **(C)** LSt. The only change observed in all five subdivisions, **(D)** dNC, **(E)** iNC, **(F)** mNCL, **(G)** lNCL, and **(H)** vNC was a significant increase in spherical DCX-positive neurons in the Trained vs. Baseline control groups. *P < 0.05; **P < 0.01. *N* = 4 data points for Baseline, Trained and No-Association; *N* = 6 for Undertrained.

The boundaries of Area X could be clearly delineated from the surrounding MSt since it was more myelinated and contained comparatively fewer DCX-positive neurons (Supplementary Figure 12D). An interaction effect was observed for spherical cells (*P* < 0.05) between the area and experimental condition. Furthermore, a one-way ANOVA/ Kruskal-Wallis Rank Sum test revealed significantly greater numbers of spherical neurons in Area X (*P* < 0.05 and *P* < 0.01; Figure 6A and Supplementary Table 8) and MSt (*P* < 0.01; Figure 6B and Supplementary Table 8) of Trained birds vs. those in Baseline and Undertrained groups, whereas there were no differences in the number of fusiform, multipolar and total numbers of DCX-labeled neurons in these striatal regions across different experimental groups. Whereas DCX-labeled neurons were present in LSt (Supplementary Figure 12E), there were no significant differences in their number across different groups of house crows (Figure 6C). Our results, therefore, suggest that training on the visual discrimination task may induce an increase in spherical DCX-positive neurons in the medial striatum of Trained birds.

##### 3.3.1.2 Caudal nidopallium

There were no interactions for any DCX-positive cell type in NC across any of the experimental groups of house crows (see Supplementary Figure 12F for staining patterns). The only difference was a significant increase in spherical cells in Trained vs. Baseline birds in all subdivisions of NC (*P* < 0.05; Figures 6D–H and Supplementary Table 8).

#### 3.3.2 Morphometric analysis of multipolar DCX-positive neurons in MSt

There was no effect of interactions between area and experimental condition for any parameter analyzed. A one-way ANOVA/Kruskal-Wallis test revealed a significant increase in neurite length of DCX-positive multipolar neurons in No-Association and Trained birds compared to Baseline and Undertrained birds in Area X and MSt (Supplementary Figures 13A, B; *P* < 0.001; Supplementary Table 9). Furthermore, neurite length was significantly greater in DCX-positive neurons of No-Association vs. Trained crows in both areas (*P* < 0.05 and *P* < 0.01; Supplementary Figure 13B and Supplementary Table 9). Neurite branching was greater in DCX-labeled neurons of No-Association birds vs. that in Trained, Baseline and Undertrained groups in Area X and MSt (*P* < 0.05 and *P* < 0.001; Supplementary Figure 13C and Supplementary Table 9). The Dunn’s *post-hoc* test demonstrated that neurite field area of these neurons in Area X and MSt of No-Association and Trained crows was higher than that in Baseline and Undertrained birds (*P* < 0.001; Supplementary Figure 13D and Supplementary Table 9). Furthermore, neurite field area of DCX-labeled neurons in No-Association birds was greater than that of Trained birds (*P* < 0.05 and *P* < 0.01) for both areas. We also found an expansion of the neurite field of these neurons in Undertrained vs. Baseline birds (*P* < 0.01; Supplementary Figure 13D and Supplementary Table 9). Additionally, the area of DCX-positive neuronal somata was significantly greater in Undertrained, Trained, and No-Association groups vs. that in Baseline controls in Area X and MSt (*P* < 0.01 and *P* < 0.001; Supplementary Figure 13E and Supplementary Table 9).

These findings were reflected in Sholl analysis, demonstrating significant differences in the number of intersections and neurite length between Sholl radii 28–68 μm in DCX-positive neurons in Area X. *Post-hoc* tests (Dunn’s or Tukey’s) demonstrated that neurites of DCX-positive neurons in No-Association and Trained birds had significantly more intersections and were longer compared to those of Baseline and Undertrained birds (*P* < 0.05, *P* < 0.01, and *P* < 0.001; Supplementary Figures 13F, G and Supplementary Table 10). Both parameters were found to be even greater for neurites of DCX-labeled neurons in No-Association vs. Trained birds (*P* < 0.05 and *P* < 0.01). Furthermore, there were a greater number of intersections and an increase in neurite length between Sholl radii 38–58 μm in neurons of Undertrained vs. Baseline birds (*P* < 0.05 and *P* < 0.01; Supplementary Figures 13F, G and Supplementary Table 10). Similar differences were observed in MSt for all groups between Sholl radii 18–68 μm except for No-Association vs. Trained birds (*P* < 0.05, *P* < 0.01, and *P* < 0.001; Supplementary Figures 13H, I and Supplementary Table 10).

##### 3.3.2.1 Morphometric analysis of active and inactive DCX-positive neurons

**FIGURE 7:**
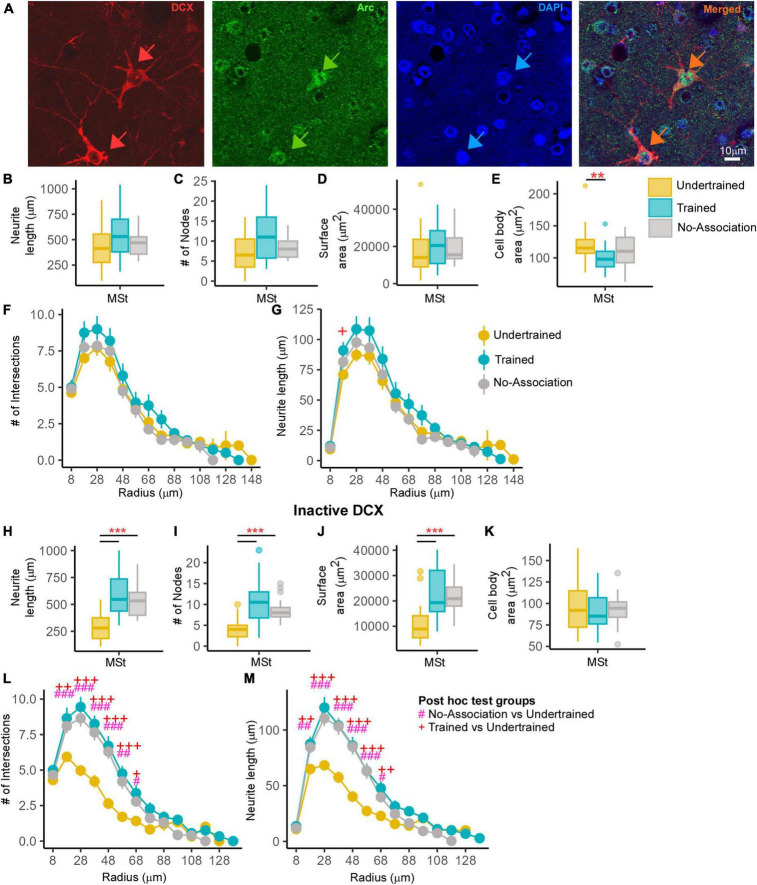
An analysis of changes in active and inactive DCX neurons in MSt. **(A)** A representative image from a Trained bird showing two DCX-labeled neurons (red; red arrows). The same section was labeled for Arc (green) and the nuclear label, DAPI (blue). The merged image demonstrates that these DCX-labeled neurons were positive for Arc and “active.” Very few double-labeled multipolar DCX neurons were found in MSt and the distribution of Arc was observed in the cytosol as well as in the nucleus of these neurons. Scale bar, 10 μm. An analysis of these DCX and Arc-labeled neurons in MSt demonstrated no significant differences across Undertrained, Trained, and No-Association groups in terms of their **(B)** neurite length, **(C)** number of nodes, and (**D**) neurite field area. There was a significant decrease in the **(E)** area of somata in Trained vs. Undertrained birds. Using Sholl analysis, we found that there were no changes in the **(F)** number of intersections and **(G)** and that the neurite length was significantly higher at only one Sholl radius in the Trained vs. that in the Undertrained group. In the inactive DCX neuronal population in MSt, we observed significant increases in the **(H)** neurite length, **(I)** number of nodes, and **(J)** neurite field area in the Trained and No-Association groups compared to that in the Undertrained category. **(K)** There were no differences in the size of somata in any of the experimental groups. A Sholl analysis demonstrated an increase in the **(L)** number of intersections and **(M)** neurite length in the Trained and No-Association groups. */^#^/^+^*P* < 0.05; **/^##^/^++^*P* < 0.01; ***/^###^/^+++^*P* < 0.001. *N* = 20 data points for Trained and No-Association; *N* = 30 for Undertrained, wherein *, #, and + represent significant differences.

Whereas fusiform DCX-positive neurons were not double-labeled for Arc (Supplementary Figure 14A), spherical or multipolar double-labeled neurons were very sparsely distributed in the house crow brain (Figure 7A and Supplementary Figures 14A, B). As for DARPP-32-labeled neurons, active (Arc- and DCX-labeled) and inactive (only DCX-positive) multipolar neurons in MSt, mNCL and lNCL were reconstructed for further analysis. Since none of the DCX-positive neurons in Area X were double-labeled, this area was excluded from further analysis.

##### 3.3.2.2 Structural changes in Arc- and DCX-positive neurons within MSt

Whereas there were no differences in neurite length, number of nodes or neurite field area (Figures 7B–D), neuronal somata of Arc- and DCX-double-labeled neurons in MSt were significantly larger in Undertrained vs. Trained crows (*P* < 0.01; Figure 7E and Supplementary Table 11). We also found no significant differences in the number of intersections (Figure 7F). However, neurite length had increased significantly at one Sholl radius (18 μm) in these neurons in Trained vs. Undertrained birds (*P* < 0.05; Figure 7G and Supplementary Table 12).

In contrast, neurite length (*P* < 0.001; Figure 7H and Supplementary Table 11), number of nodes (*P* < 0.001; Figure 7I and Supplementary Table 11) and neurite field area (*P* < 0.001; Figure 7J and Supplementary Table 11) were significantly greater in inactive DCX-labeled neurons within MSt of Trained and No-Association birds vs. that in Undertrained birds. Unlike activated neurons, there were no changes in the area of cell bodies of inactive DCX neurons across different groups (Figure 7K). Besides these measures, Sholl analysis revealed significantly greater differences in the number of intersections and neurite length of DCX-positive neurons between Sholl radii 18–68 μm of Trained and No-Association groups vs. that in Undertrained birds (*P* < 0.05, *P* < 0.01 and *P* < 0.001; Figures 7L, M and Supplementary Table 12).

Comparisons between active and inactive DCX-labeled multipolar neurons in MSt revealed no significant differences in complexity or soma size in Trained crows (Supplementary Figures 15A–E). Furthermore, the area of inactive DCX-positive somata was significantly lower than that of active neurons in No-Association birds (*P* < 0.05; Supplementary Figure 15E and Supplementary Table 13). Interestingly, neurite length (*P* < 0.01), number of endings (*P* < 0.001), number of nodes (*P* < 0.01), neurite field area (*P* < 0.01; Supplementary Figures 15A–D and Supplementary Table 13) and cell body area (*P* < 0.01; Supplementary Figure 15E and Supplementary Table 13) were significantly higher in active vs. inactive DCX-labeled neurons of Undertrained birds. These findings suggest that active DCX-positive multipolar neurons in MSt increase in complexity specifically in Undertrained birds.

#### 3.3.3 Complexity of DCX-positive neurons in NC

We found that neurite length, number of nodes, and neurite field area of multipolar DCX-labeled neurons in NC of Undertrained, Trained and No-Association crows were significantly greater than in Baseline controls (*P* < 0.05, *P* < 0.01 and *P* < 0.001; Supplementary Figures 16A–C and Supplementary Table 14). Both Trained and No-Association group had significantly greater neurite length, number of nodes, and neurite field area compared to Undertrained birds in dNC (*P* < 0.05, *P* < 0.01, and *P* < 0.001) whereas in iNC, these parameters were significantly greater only for No-Association vs. Undertrained birds (*P* < 0.05, *P* < 0.01, and *P* < 0.001; Supplementary Figures 16A–C and Supplementary Table 14). Neurite length and number of nodes of DCX-labeled neurons in mNCL were significantly greater in Trained vs. Undertrained birds, whereas neurite field area was higher in both Trained and No-Association vs. Undertrained birds in both mNCL and lNCL (*P* < 0.05, *P* < 0.01 and *P* < 0.001; Supplementary Table 14). In lNCL, branching was significantly greater in No-Association vs. Undertrained birds whereas neurite length differences were observed for both Trained and No-Association vs. Undertrained birds (*P* < 0.05, *P* < 0.01, and *P* < 0.001; Supplementary Figures 16A, B and Supplementary Table 14). Similarly, neurite length and neurite field area of DCX-labeled neurons in vNC were significantly greater in No-Association vs. Undertrained birds (*P* < 0.05 and *P* < 0.01; Supplementary Figures 16A, C and Supplementary Table 14). The area of DCX-positive neuronal somata was significantly greater in No-Association vs. Baseline birds in dNC (*P* < 0.05; Supplementary Table 14) and also Trained vs. Baseline birds in iNC (*P* < 0.01; Supplementary Figure 16D and Supplementary Table 14). Sholl analysis demonstrated an increase in the number of intersections and neurite length at most of the Sholl radii in Undertrained, Trained and No-Association vs. Baseline birds in all subdivisions of NC (*P* < 0.05, *P* < 0.01 and *P* < 0.001; Supplementary Figures 16E–H, 17A–F and Supplementary Table 15).

##### 3.3.3.1 Morphometric analysis of active and inactive DCX-positive neurons in NCL

**FIGURE 8:**
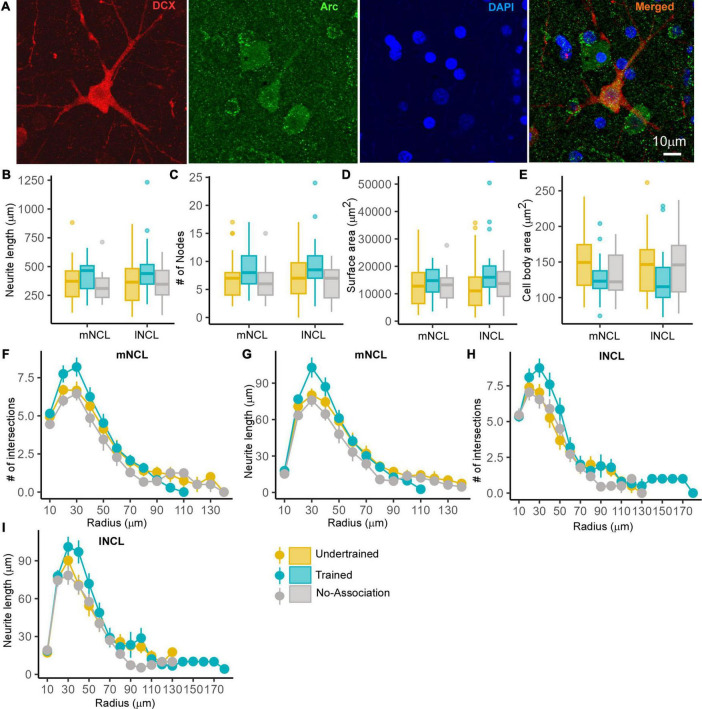
The structure of DCX and Arc double-labeled neurons in NCL. A representative neuron from the lNCL region of the Undertrained group showing colocalization of **(A)** DCX (red) with Arc (green), also labeled for DAPI (blue). Scale bar, 10 μm. An analysis of changes in the structure of these active DCX neurons demonstrates that there were no significant differences in the **(B)** neurite length, **(C)** number of nodes and area **(D)** the neurite field, or **(E)** somata across different experimental groups. Whereas a Sholl analysis demonstrated that the **(F)** number of intersections in mNCL were greater in Trained birds vs. those in other groups, these differences were not significant. There was a non-significant increase in the **(G)** neurite length of double-labeled mNCL neurons in the Trained vs. the Undertrained and No-Association groups. A similar analysis of DCX and Arc double-labeled neurons in lNCL revealed that there were no significant differences in the **(H)** number of intersections or **(I)** neurite length. As seen for such neurons in mNCL, there were non-significant increases in DCX and Arc double-labeled neurons in the lNCL of the Trained group vs. other groups. *N* = 20 data points for Trained and No-Association; *N* = 30 for Undertrained.

Since there was greater activation in mNCL and lNCL following performance on the visual discrimination task, we decided to analyze Arc- and DCX-double labeled neurons specifically in these regions. Neurons positive for Arc and DCX were sparsely distributed in NCL (Figure 8A). There were no changes in neurite length, number of nodes, neurite field area and area of the somata of these neurons in mNCL and lNCL (Figures 8B–E) or the number of intersections and neurite length measured by Sholl analysis based on experimental condition (Figures 8F–I).

**FIGURE 9:**
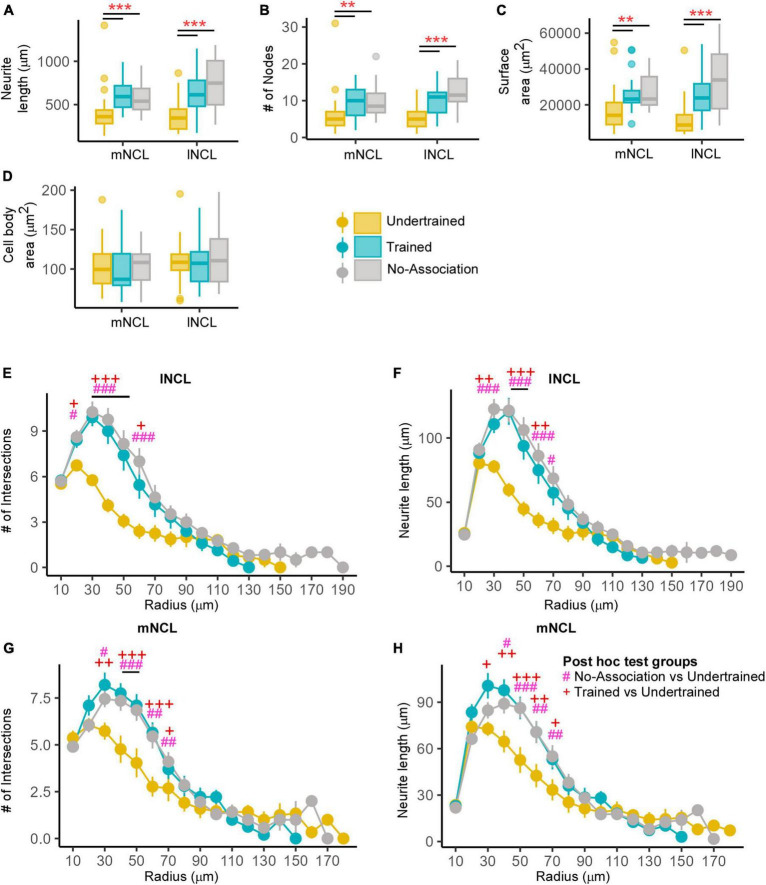
Structural changes in inactive DCX-positive neurons in the NCL. The **(A)** neurite length and **(B)** number of nodes were significantly greater in Trained and No-Association birds compared to those in the Undertrained category. **(C)** The neurite field of inactive DCX-labeled neurons in the Trained and No-Association groups was significantly larger in both mNCL and lNCL compared to that of Undertrained birds. **(D)** The area of the somata of these neurons was similar in all three groups. A Sholl analysis performed on reconstructed lNCL neurons demonstrated an increase in the **(E)** number of intersections and **(F)** neurite length in Trained and No-Association birds compared to that of Undertrained birds. Similar results for these parameters were observed for mNCL neurons, that is, an increase in the **(G)** number of intersections and **(H)** neurite length in Trained and No-Association birds vs. that in the Undertrained group. ^#^*P* < 0.05; **/^##^*P* < 0.01; ***/^###^*P* < 0.001. *N* = 20 data points for Trained and No-Association; *N* = 30 for Undertrained, wherein *, #, and + represent significant differences.

In contrast, the complexity of inactive (Arc-negative) DCX-labeled multipolar neurons in lNCL and mNCL varied across different groups of experimental birds. The Dunn’s *post-hoc* test demonstrated that neurite length (*P* < 0.001; Figure 9A and Supplementary Table 16), number of nodes (*P* < 0.01 and *P* < 0.001; Figure 9B and Supplementary Table 16), and neurite field area (*P* < 0.01 and *P* < 0.001; Figure 9C and Supplementary Table 16) were significantly greater in Trained and No-Association vs. Undertrained birds. There were no differences in the size of somata of inactive DCX-positive neurons in lNCL and mNCL in any of the groups (Figure 9D). Lastly, Sholl analysis demonstrated that the number of intersections and neurite length of inactive DCX neurons was higher in Trained and No-Association crows vs. that in Undertrained birds in both lNCL and mNCL (*P* < 0.05, *P* < 0.01 and *P* < 0.001; Figures 9E–H and Supplementary Table 17).

A comparison of active and inactive DCX-labeled neurons across various experimental groups revealed that there were no significant differences in complexity based on neurite length, number of endings and nodes, and neurite field area in mNCL and lNCL of Undertrained birds (Supplementary Figures 18A–I). However, in both these regions, the area of the somata of active DCX-positive neurons was greater than that of inactive DCX-positive neurons (*P* < 0.001; Supplementary Figures 18E, J and Supplementary Table 18). In lNCL of Trained birds, the only significant change was an increase in neurite length of inactive vs. active DCX-labeled neurons (*P* < 0.05; Supplementary Figure 18A and Supplementary Table 18). In contrast, neurite length (*P* < 0.01; Supplementary Figure 18F and Supplementary Table 18) and neurite field area (*P* < 0.001; Supplementary Figure 18I and Supplementary Table 18) were significantly greater in inactive vs. active DCX-labeled neurons in mNCL of Trained birds. However, the somata of inactive vs. active neurons were significantly smaller in mNCL (*P* < 0.01; Supplementary Figure 18J and Supplementary Table 18). All measures of complexity of neurites for inactive DCX-positive neurons were significantly higher in No-Association birds in both lNCL and mNCL (*P* < 0.05 and *P* < 0.001; Supplementary Figures 18A–I and Supplementary Table 18), although their cell bodies were smaller than those of active neurons in these regions (*P* < 0.05 and *P* < 0.001; Supplementary Figures 18E, J and Supplementary Table 18). These results suggest that active DCX-positive neuronal somata increase in size in all three groups, but the complexity of neurites increases significantly in inactive vs. active DCX-positive neurons only in Trained and No-Association birds.

## 4 Discussion

Whereas training on the visual discrimination task led to an increase in activation throughout NC in house crows based on Arc expression, we found that dNC, mNCL, lNCL, and iNC of Trained and No-Association birds were significantly more activated vs. that in Baseline birds. The highest levels of neural activity overall were present in lNCL of Trained crows, which was similar to results in carrion crows (*Corvus corone*) demonstrating that NCL is involved in predicting behavioral rules (Nieder, 2017), working memory (Diekamp et al., 2002), reversal learning (Hartmann and Gunturkun, 1998), reward valuation (Dykes et al., 2018), and performance on multicomponent behavioral tasks (Rook et al., 2020). Interestingly, training on visual discrimination leads to activation of other parts of NC including dNC and iNC in house crows, which needs further investigation. Besides NC, we found that MSNs and larger neurons (likely pallidal neurons and interneurons) were activated in different parts of the basal ganglia. Levels of activation were significantly higher in LSt and GP [important for motor functions in birds (Veenman et al., 1995; Reiner et al., 2004)] of Trained, No-Association, and Undertrained birds which attempted to obtain the reward vs. Baseline controls. Furthermore, Area X and MSt, which are components of the anterior forebrain pathway in zebra finches (Brainard and Doupe, 2002) were activated in crows after training on the visual discrimination task. Whereas Area X is important for song crystallization (Scharff and Nottebohm, 1991; Brainard and Doupe, 2002) and context-dependent singing (Hessler and Doupe, 1999), MSt is activated when male zebra finches perform dance-like movements while courting females (Feenders et al., 2008), in the selection of multicomponent behavior in pigeons (Rook et al., 2020), spatial and color-cued learning (Watanabe, 2001) and aversive learning (Freeman and Rose, 1999). Interestingly, MSt receives projections from the parvicellular “shell” of LMAN, which receives input from dNCL in zebra finches. Therefore, MSt in different species of songbirds may receive information about learning and decision-making processed in NC (Paterson and Bottjer, 2017).

Both PFC and the striatum are involved in reinforcement learning (Pan et al., 2014; Tanaka et al., 2015) and are extensively connected in mammals and birds (Gale and Perkel, 2010; Wood, 2021). Furthermore, local field potentials become more synchronized in these regions with learning, suggesting the strengthening of connections in the cortico-basal ganglia circuit (Antzoulatos and Miller, 2014). These findings suggest that similar mechanisms may underlie learning visual discrimination in crows.

### 4.1 Song control areas are activated following visual discrimination in house crows

Surprisingly, brain regions generally associated with song control (Brainard and Doupe, 2002) were activated after house crows performed the visual discrimination task. The song control area LMAN was significantly activated in No-Association birds vs. the Baseline group of birds. In songbirds such as zebra finches, LMAN is important for generating variability in vocalizations during the sensitive period for song learning (Scharff and Nottebohm, 1991; Ölveczky et al., 2005). Recent studies further demonstrated that LMAN lesions prevented somatosensory-based non-auditory learning which affected vocal output in zebra finches (Mcgregor et al., 2022) and an LMAN-like region in pigeons (NIML) was involved in the execution of sequence learning (Helduser et al., 2013) and serial processing (Rook et al., 2021), but not in generating variability. In our study, trial-by-trial variability would be the highest in No-Association birds since the reward was randomly associated with either of the shapes presented during the task. These findings suggest that besides vocal learning, LMAN is involved in modulating variability associated with learning the visual discrimination task in house crows.

We also observed higher activation in RA and AId of Trained crows vs. other groups. Besides projecting to syringeal musculature for controlling vocalization, RA projects to the ventral respiratory column for controlling respiration (Schmidt and Martin Wild, 2014). Since experimental crows never vocalized during or immediately after training, it is possible that this heightened activity in RA may be important for synchronizing breathing with performing the correct sequence of actions during the task. Another arcopallial region, AId, is involved in motor functions and song learning (Bottjer and Altenau, 2010). It receives topographically organized projections from NCL (Kroner and Gunturkun, 1999; Paterson and Bottjer, 2017) and projects to the optic tectum (Fernández et al., 2020). Increased activation of AId consequent to training on visual discrimination may involve a goal-directed visuomotor pathway beginning in mNCL (unpublished data) which is connected to AId and the optic tectum (Kroner and Gunturkun, 1999; Fernández et al., 2020).

For our study, we were careful not to include neural activity induced due to vocalization. Firstly, all experimental birds were in visual and auditory isolation from other birds starting 2 days prior to the behavioral experiment and also during the experiment. Secondly, we kept all experimental birds in the dark for 90 min before sacrifice, as a result of which there is minimal motor activity and no vocalization. In addition to these factors, the experimental crows never vocalized during the experiment. In earlier studies on zebra finches, period of silence when birds do not sing are associated with a lack of Arc expression in the song control nuclei (Hayase et al., 2018; Hayase and Wada, 2018). Therefore, it is likely that the induction of Arc protein in song control regions is not associated with vocalization but is more likely to be task-driven.

### 4.2 Dopaminoceptive neurons are associated with learning in house crows

Dopamine plays an important role in motivation, learning, cognition, reward and pleasure, and motor learning and the firing rates of VTA-SNc increase to signal the physical salience of the reward and reward-predicting stimuli (Schultz, 2016). As expected, dopaminergic neurons in VTA-SNc were positive for Arc in all groups of birds other than the unrewarded Baseline controls. Furthermore, NCL contained DARPP-32-labeled dopaminoceptive neurons of which some were positive for Arc, showing that they participated in visual discrimination. Although there were no changes in their number, for the first time, we have demonstrated that the complexity of their neurites and soma size increased significantly across NC, especially in mNCL and lNCL of Trained house crows. These findings suggest that learning to associate specific types of behavior with a reward leads to an increase in the plasticity of dopaminoceptive neurons.

The discovery of experience-induced neurite plasticity began with visual deprivation experiments performed on kittens by Hubel and Weasel (Kandel, 2009). Since then, plasticity in cortical and subcortical circuits was mainly explored in the context of injuries (Chen and Zheng, 2014), experience (Shansky et al., 2009; Bose et al., 2010) and learning (d’Aquin et al., 2022). In adult cats, binocular retinal lesions lead to sprouting of long-range fibers into the reorganized visual cortex (Darian-Smith and Gilbert, 1994). Besides injury, GABAergic non-pyramidal cells demonstrate an expansion and retraction of dendritic tips in layer 2/3 of the visual cortex in mice daily (Lee et al., 2006), suggesting a role for a cell-type specific population in adult neural plasticity. Additionally, in the pyramidal neurons of adult rats, deafness led to a decrease in apical dendrite length but no change in basal dendrites, whereas exposure to an enriched environment led to an increase in the length of basal dendrites but no change in that of apical dendrites in the primary auditory cortex (Bose et al., 2010). Besides sensory deprivation changes in the external environment and learning leads to changes in neuronal plasticity (Comeau et al., 2010). In male rats, complex housing leads to a decrease in the dendritic field of pyramidal neurons in the cingulate cortex (region 3), mPFC (CG3) layer 5 (L5) and an increase in this parameter in layer 3 (L3) of the orbitofrontal cortex (OFC). Furthermore, Comeau et al. demonstrated that training on the T-maze leads to an increase in the dendritic field and spine density of L5 neurons in the CG3 and a decrease in these parameters in L3 neurons in the OFC. They also found that spatial reversal learning in a parallel alley maze causes a decrease in the dendritic field in both areas. This study suggests that the plasticity induced by different kinds of experience varies in different areas and layers of the cortex (Comeau et al., 2010). Additionally, in a forelimb-reaching task, neurons in layer 2/3 of the motor cortex undergo dendritic remodeling. There was a transient increase in dendritic complexity in the distal parts of apical dendrites, whereas there was a decrease in the complexity of basal dendrites (Streffing-Hellhake et al., 2010). These studies suggest that mature neurons in the adult brain are capable of exhibiting plasticity in the active neural circuits.

**FIGURE 10:**
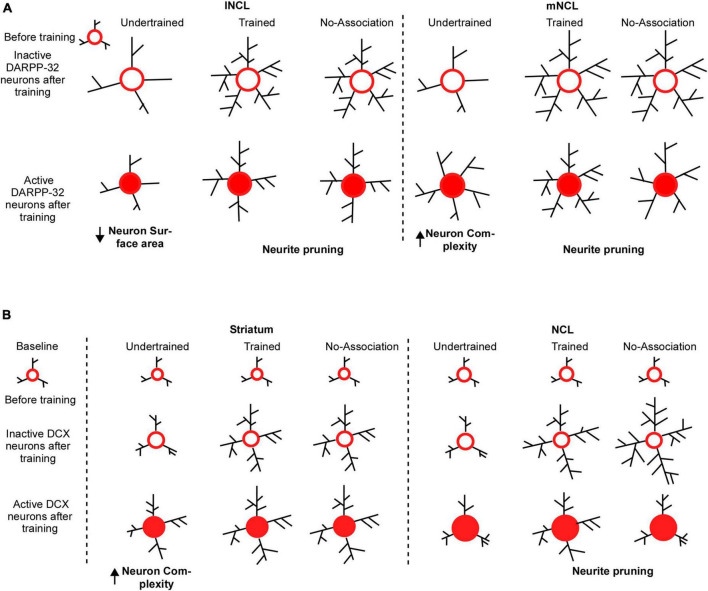
Possible mechanisms underlying changes in the structure of DARPP-32 and DCX neurons in the striatum and NCL for visual discrimination task. **(A)** We assume that dopaminoceptive (DARPP-32-positive) neurons in lNCL and mNCL regions before training (in the Undertrained, Trained, and No-Association groups) are similar to those in the Baseline group. After training, there is an increase in the neurite field in inactive dopaminoceptive neurons in the lNCL of the Undertrained group, which is greater than that of active Arc- and DARPP-32-positive neurons. In contrast, active neurons demonstrate an increase in neuronal complexity in mNCL. Inactive (Arc-negative) DARPP-32 neurons in both lNCL and mNCL from Trained and No-Association groups are more complex than active (Arc-positive) dopaminoceptive neurons in these regions. Since in each case, active neurons have fewer neurites, it is possible that they are pruned as a result of learning the visual discrimination task. In the striatum (MSt), **(B)** the structure of immature DCX-positive neurons in all experimental groups before training on the visual discrimination task is likely similar to that in the Baseline group. With training (in the Trained and No-Association groups), there is an increase in the complexity and size of somata of DCX-positive neurons which are not part of active neural circuits. Whereas immature neurons which appear to be incorporated into active neural circuitry underlying the task in the Undertrained group become more complex, there was no difference in their structure in the Trained and No-Association groups. Similar changes were observed in DCX-labeled neurons in NCL. However, unlike MSt neurons, there is a decrease in complexity of these neurons suggestive of neurite pruning in Trained and No-Association birds. Furthermore, despite the fact that neurons double-labeled for DCX and Arc (a part of the active circuit) underwent changes in their structure in Undertrained birds, these changes were not significant and were not as pronounced as those in MSt.

The findings from our study also demonstrated learning induced plasticity in the mature neurons, these changes were more pronounced in regions such as NCL compared to other NC subdivisions which may suggest that a greater number of neurons in these areas get recruited in the learning process. We found that both active and inactive dopaminoceptive neurons in mNCL and lNCL were more complex in Trained and No-Association birds vs. Undertrained birds, suggesting that training on visual discrimination led to an increase in branching and likely, the number of synapses. Comparisons between these sets of neurons revealed that inactive dopaminoceptive neurons were more complex than active ones in lNCL and mNCL of Trained and No-Association birds. However, active neurons in mNCL were more complex than inactive ones in Undertrained birds (Figure 10A). These findings suggest that the initial phase of learning leads to an increase in complexity and/or synapses of DARPP-32-labeled neurons in mNCL, whereas extensively branched neurites of dopaminoceptive neurons in lNCL and mNCL are pruned to retain only task-specific connections (Hawes et al., 2015) with training. Whereas our results strongly suggest the possible involvement of other NC subdivisions in learning, further studies are needed to address the extent of involvement of other subdivisions and what part of learning and decision-making process are modulated by these regions.

### 4.3 Learning-induced adult neurogenesis and structural changes in adult-born neurons in the house crow brain

Learning may lead to neurogenesis in house crows (Taufique et al., 2018) and other species (Urakawa et al., 2007; Tronel et al., 2010; Lemaire et al., 2012) even in adulthood. For example, the transient depletion of immature neurons in the mouse hippocampus causes deficits in learning the active place avoidance task (Vukovic et al., 2013) and they are important for reconsolidation of task-induced memories (Lods et al., 2021). Amongst avian species, both fusiform and multipolar DCX-labeled neurons increase in number in the nidopallium of adult house crows (Taufique et al., 2018) and pigeons (Mehlhorn et al., 2022), which was correlated with stress and homing behavior, respectively. In our study, the increase in immature spherical DCX-labeled neurons in the striatum and NC of Trained vs. Undertrained and Baseline categories of house crows (indicative of adult neurogenesis) may be necessary or permissive for learning and decision-making. Furthermore, the significant increase in spherical DCX-labeled neurons in different compartments of the avian basal ganglia (Area X, MSt and LSt) in Trained birds vs. other groups may contribute to habitual learning and habit formation (Malvaez and Wassum, 2018), learning extinction (Goodman et al., 2016; Goodman and Packard, 2018), reinforcement learning (Tanaka et al., 2015) and decision-making (Balleine et al., 2007) besides vocal learning (Scharff and Nottebohm, 1991), as seen in the mammalian dorsal striatum. Interestingly, striatal lesions and exposure to an enriched environment have been linked to an increase in migrating DCX-positive neurons in the dorsal striatum of rodents as well (Urakawa et al., 2007). However, to actually prove that the increases observed in DCX neurons were due to changes in the number of newly generated neurons in adult birds (La Rosa et al., 2020) further studies are required, involving the administration of external S-phase markers such as BrdU (Bromodeoxyuridine) or EdU (5-ethynyl-2′-deoxyuridine) in conjunction with internal markers such as DCX, at specific time points during the training period.

Our results also demonstrated that DCX-labeled multipolar neurons were significantly more complex in different divisions of NC, Area X and MSt of Trained and No-Association birds vs. those of other groups which is suggestive of increased neural plasticity in these circuits. In NCL, inactive DCX-positive neurons were more complex and appeared to be pruned when they became parts of active circuits. Whereas active DCX-labeled neurons in MSt demonstrated fewer changes in complexity across different experimental groups, they were more complex compared to inactive DCX-labeled neurons in MSt of Undertrained birds (Figure 10B). In rodents, the dorsomedial part of the striatum is involved in attentive decision-making, whereas its dorsolateral component underlies automatizing responses during the latter part of the learning process (Whishaw et al., 1987; Yin et al., 2004; Clouse et al., 2009) and neurons in these regions undergo changes in dendritic complexity with learning (Hawes et al., 2015). Changes in the No-Association group were similar to those in the Trained group probably due to the trial-and-error learning method employed by the crows. Overall, these results suggest that inactive neurons become more complex in MSt in Undertrained crows during the initial part of learning the visual discrimination task.

From our results, we cannot provide direct evidence of the differences observed due to learning-induced changes in neuronal complexity between inactive and active neurons. This is because we did not tag neurons which were initially activated during the task and looked for the changes in neurite plasticity over time, as learning progressed. We used the immediate early gene Arc since it is reported to be better correlated to the behavioral task demands as reported in a study involving hippocampal dependent and hippocampal independent behavioral tasks in rats, compared to the expression of other IEGs, zif268 and c-fos (Guzowski et al., 2001). Our results cannot rule out the possibility that immediate early genes other than Arc are expressed in inactive neurons. Moreover, since we did not perform experiments to study the time-course of activation of different sets of neurons and the changes in their complexity in the Trained group, we cannot prove whether inactive neurons had expressed Arc at some earlier time point or if they never expressed Arc. Future studies addressing the time course of these changes and tagging the active neurons are needed to address these questions.

### 4.4 Why are morphometric changes in DARPP-32- or DCX-positive neurons in No-Association crows similar to those in Trained birds?

An interesting conundrum is provided by No-Association birds. Despite being exposed only to two blocks of trials (similar to Undertrained birds) of the visual discrimination task, structural changes undergone by DARPP-32- or DCX-labeled neurons in the No-Association group are comparable to those observed in Trained birds. It is possible that employing two extreme strategies for obtaining the food reward, that is, goal-oriented behavior in case of Trained birds and trial-and-error learning in No-Association birds results in similar changes in the complexity of neurons involved in the visual discrimination task. Alternatively, the strengthening of functional connections coding for the correct strategy to obtain rewards may vary across different experimental groups. For example, neural circuits in Undertrained birds, which are at an initial phase of learning may not have been strengthened leading to low and variable neural activity and fewer changes in the complexity of mature and immature neurons in the striatum and NCL. Following extensive training on the task, it is likely that neural circuits responsible for obtaining the food reward may have been strengthened, leading to higher levels of neural activity and more elaborate neurites in neurons within NCL. Finally, in the No-Association group, since rewards are associated randomly with the shapes, the cognitive load may be the highest. Hence, high levels of neural activity as well as morphometric changes in DCX-labeled neurons of No-Association birds are similar to those in Trained birds.

### 4.5 Differences between different NCL subdivisions

The number of Arc positive neurons was higher in lNCL than mNCL although this difference was not significant. Overall, even under normal conditions with no training, DARPP-32 neurons in mNCL have larger neurite fields compared to those in other subdivisions of NC (Sen et al., 2019). With training, we observed subtle differences between the two NCL subdivisions in different experimental groups. The differences observed in DARPP-32-labeled neurons between inactive and active neurons in the Trained group vs. other groups were more pronounced in case of lNCL than mNCL. However, these differences were not observed in immature DCX-labeled multipolar neurons present in mNCL and lNCL. Hence, it is likely that the mature neurons might be more attuned to learning-related differences than the immature neurons. Alternatively, the dopaminoceptive neuronal population may be more sensitive to task-related differences.

## 5 Conclusion

Our results suggest that diverse brain areas such as the striatum and caudolateral nidopallium are involved in learning visual discrimination in corvids. Furthermore, as demonstrated by earlier studies on corvids which have performed electrophysiological recordings from NCL, our results suggest that differences in activation and neuronal complexity in medial and lateral NCL are linked to learning. Additionally, increased adult neurogenesis and structural changes in dopaminoceptive and immature neurons may be correlated with learning in the house crow brain.

## Data availability statement

The original contributions presented in the study are included in the article/Supplementary material, further inquiries can be directed to the corresponding author.

## Ethics statement

The animal study was approved by the Institutional Animal Ethics Committee, NBRC, Manesar (under CCSEA). The study was conducted in accordance with the local legislation and institutional requirements.

## Author contributions

PP: Conceptualization, Data curation, Formal analysis, Investigation, Methodology, Validation, Visualization, Writing – original draft. MR: Data curation, Investigation, Writing – original draft. SI: Conceptualization, Funding acquisition, Investigation, Project administration, Resources, Supervision, Validation, Writing – review and editing.
