# Supplementary material for: Changes in the dopaminergic circuitry and adult neurogenesis linked to reinforcement learning in corvids
Source: Front Neurosci. 2024 May 14;18:1359874. doi: 10.3389/fnins.2024.1359874 (PMC11130420; doi:10.3389/fnins.2024.1359874)
Supplement: Supplementary file 1 [file Data_Sheet_1.pdf]

SUPPLEMENTARY FIGURES AND FIGURE LEGENDS

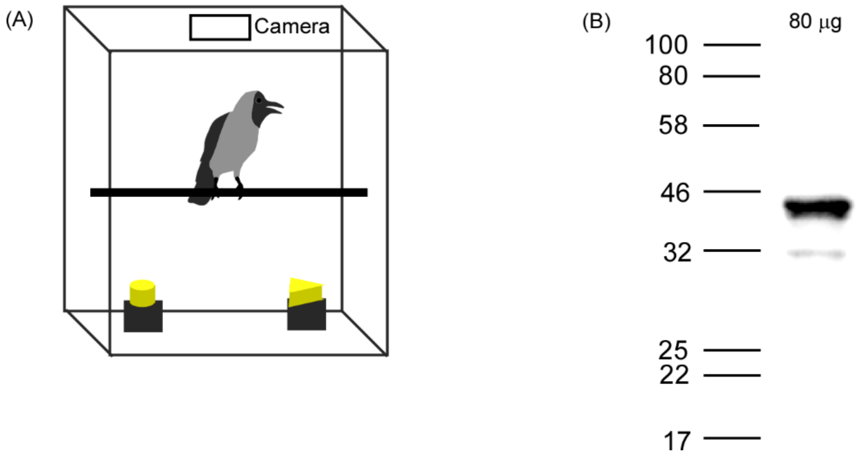

Supplementary Figure 1. A schematic of the behavioral setup and the validation of DCX antibody.

(**A**) A schematic of the behavioral setup, demonstrating the position of the camera, perch, and the two shapes positioned inside the cage. (**B**) An intense band was obtained at ~40kD by performing a western blot on house crow brain tissue, demonstrating the specificity of the DCX antibody (sc-271390, Anti-Doublecortin Antibody (E-6); RRID: AB_10610966, Santa Cruz Biotechnology).

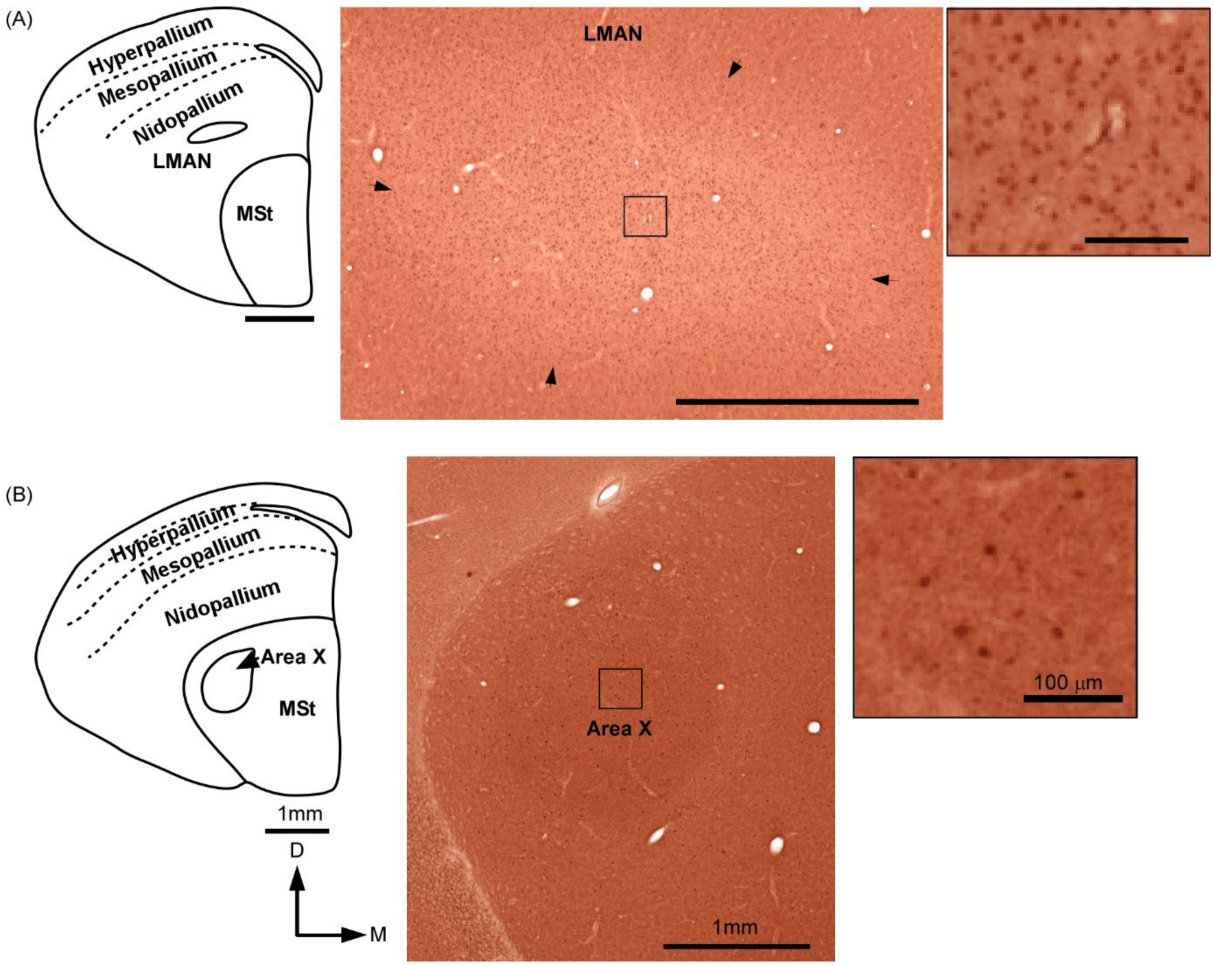

Supplementary Figure 2. Arc staining patterns in the anterior forebrain.

(**A**) The schematic shows the location of LMAN in the anterior forebrain. The photomicrograph (center) demonstrates Arc staining in LMAN. The boundaries of LMAN (*arrows*) can be clearly discerned based on Arc expression since it is heavily myelinated and contains Arc-positive neurons (visible in the inset). (**B**) The schematic on the left demonstrates the location of Area X in the anterior forebrain, which can be clearly delineated based on Arc expression and appears slightly darker than the surrounding striatum (center). The inset on the right demonstrates Arc positive neurons in Area X. Scale bar, 1mm and 100 μm for insets. D, Dorsal; M, Medial.

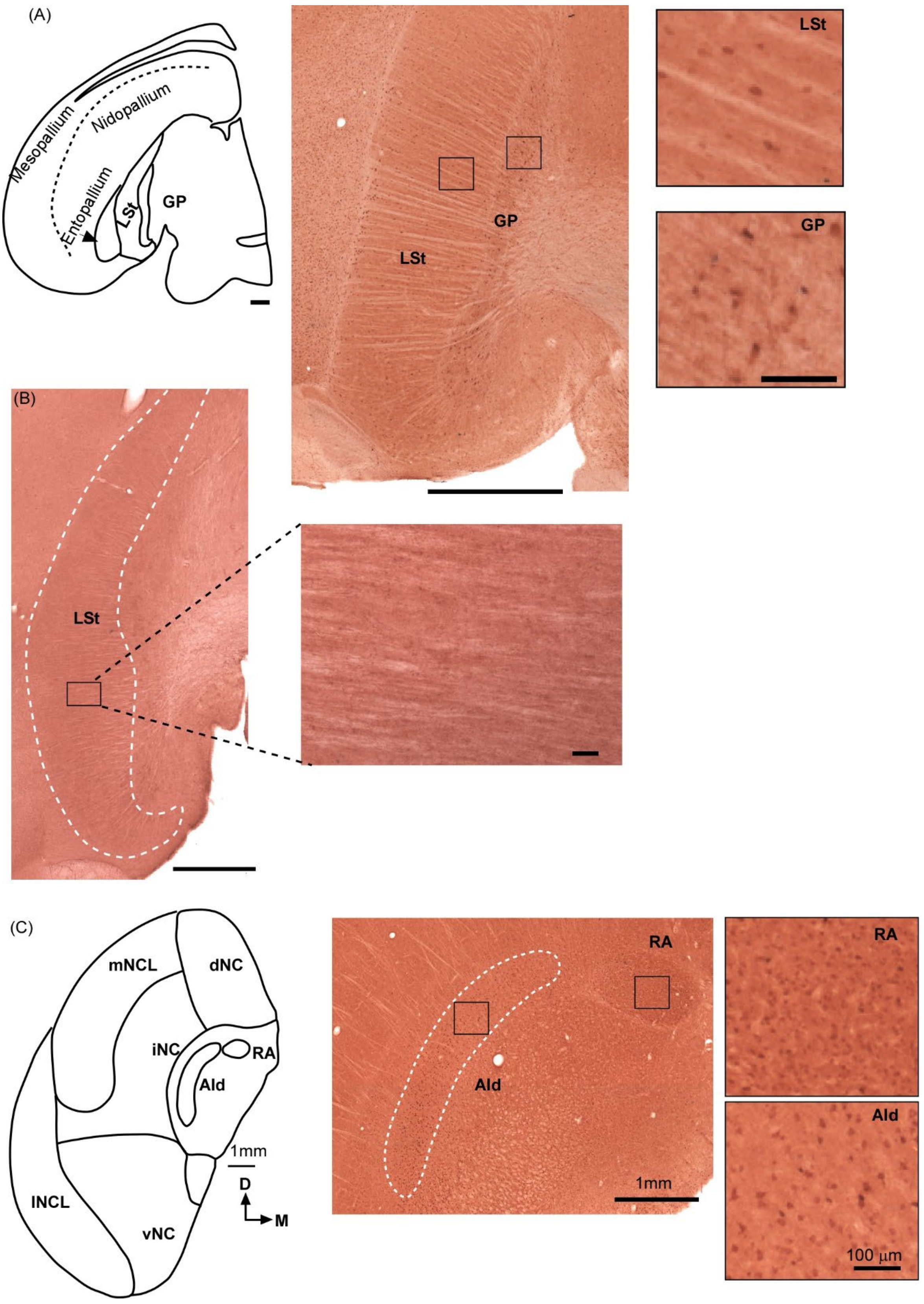

Supplementary Figure 3. Arc expression in GP, LSt and caudal nidopallial regions.

(**A**) The schematic (left) shows the locations of GP and LSt in the intermediate part of the brain, along its rostro-caudal axis. Arc-positive neurons are observed in striatal areas GP and LSt (inset on the

right). (**B**) A negative control at this level (performed by omitting the primary antibody) from the Trained group and a high-power image on the right [of the rectangular outline] demonstrates the lack of label in LSt. Scale bar for *inset*, 200 µm. (**C**) The schematic on the left depicts a section of the telencephalon in the caudal part of the brain, wherein subdivisions of the caudal nidopallium and arcopallium containing the song control nucleus RA and the adjacent dorsal intermediate arcopallium (AId). Both RA and AId can be clearly demarcated based on patterns of Arc staining across different groups of house crows (center). Both the regions contained intensely stained Arc neurons as seen in the magnified insets. Scale bar, 1mm and 100 µm for insets. D, Dorsal; M, Medial.

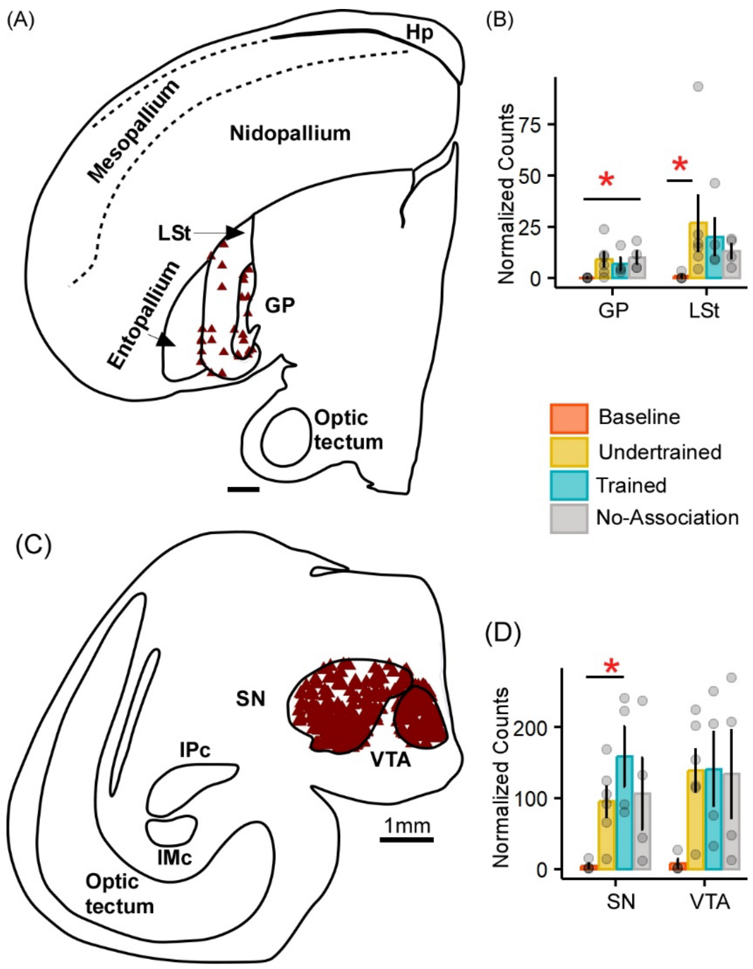

Supplementary Figure 4. Expression of Arc in GP, LSt, SN, and VTA.

(**A**) A schematic showing neural activity in GP and LSt in a house crow from the Trained group. (**B**) There were no significant differences in the number of Arc-positive (mean ± SEM) in GP and LSt across the three training groups (Undertrained, Trained, and No-Association). However, the lowest number of Arc-labeled neurons was present in the Baseline group. (**C**) A schematic showing Arc expression in SN and VTA in the house crow midbrain from the Undertrained group. (**D**) The number

of Arc-positive neurons (mean ± SEM) in SN and VTA were similar in Undertrained, Trained, and No-Association groups following the visual discrimination task, suggesting that these groups were equally motivated to obtain the food reward. There were significantly fewer Arc-positive neurons in the Baseline group since they were not trained to associate shapes used for visual discrimination with a reward. Scale bar, 1mm. *, $P < 0.05$. N = 4 data points for Baseline, Trained and No-Association birds; N = 6 for Undertrained birds.

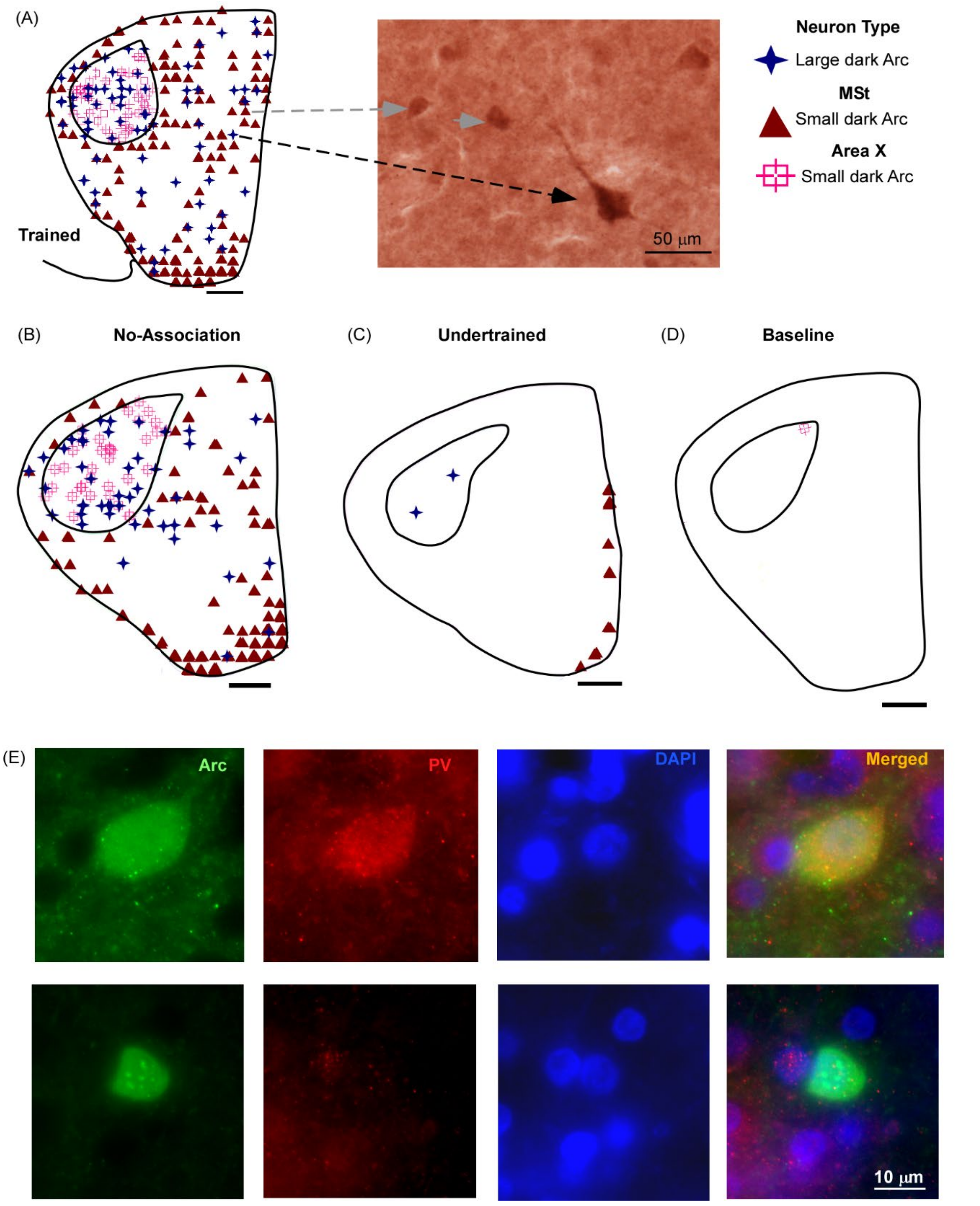

Supplementary Figure 5. Arc expression in the anterior striatum.

(**A**) A schematic of Area X and MSt showing Arc expression in a Trained crow and a high magnification image in the right panel showing large (*black arrow*) and small (*gray arrows*) Arc-positive neurons. A comparison of Arc expression in MSt and Area X in the (**B**) No-Association, (**C**) Undertrained, and (**D**) Baseline groups are shown in schematics, Scale bars for **A-D**, 1mm. (**E**) Both large and small neurons were labeled for Arc. Double-labeling for Arc (first column) and parvalbumin

(PV, second column, shown here for a house crow from the No-Association group) was performed to determine the identity of Arc-labeled neurons. The upper row demonstrates a large PV-labeled neuron which is also positive for Arc, suggesting that it may be a striatal interneuron or a pallidal neuron (Reiner et al., 2004a). The lower row demonstrates an Arc-positive neuron not labeled with PV, which is typical of non-GABAergic interneurons. Scale bar, 1mm, and 10μm.

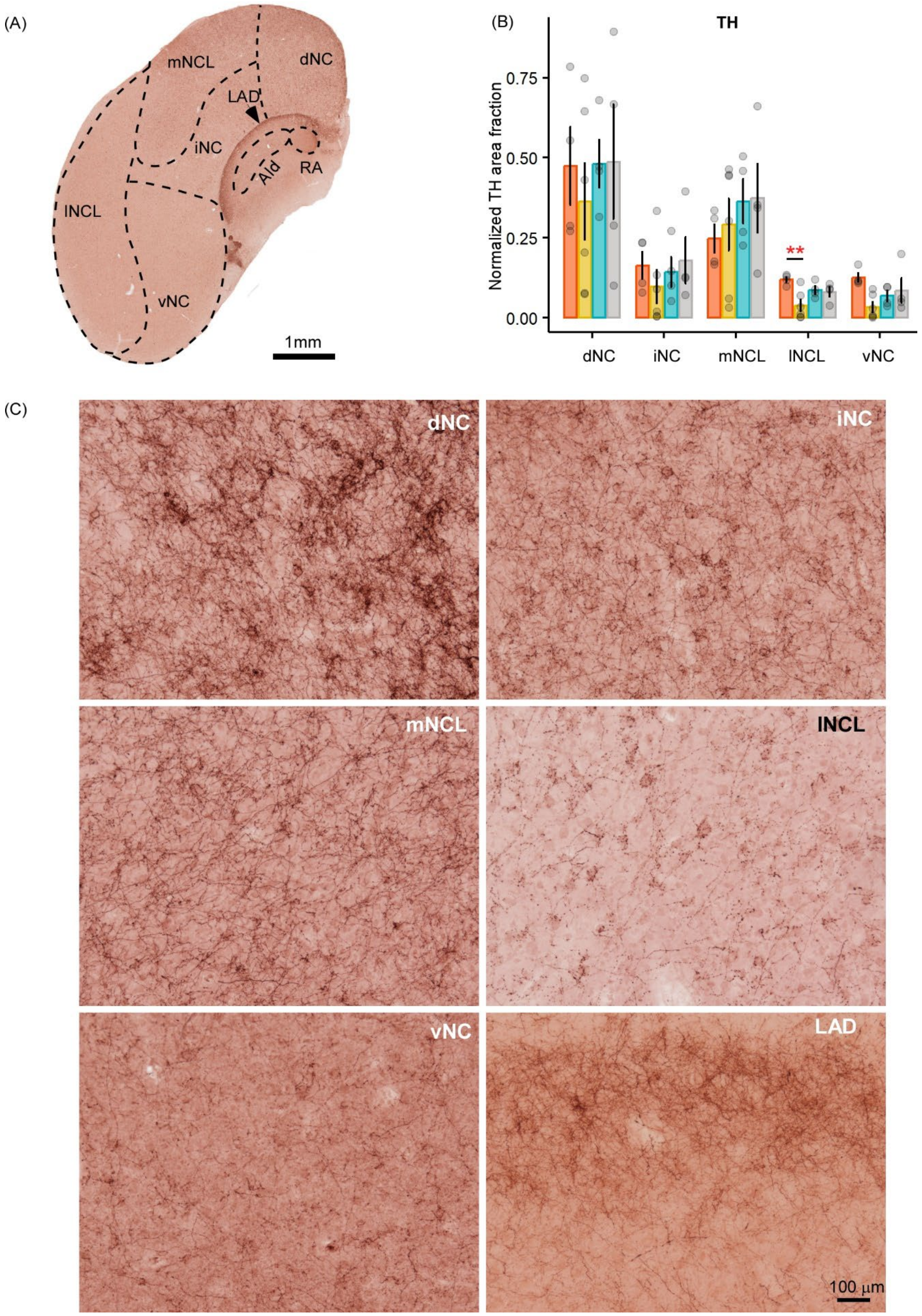

Supplementary Figure 6. Staining for Tyrosine hydroxylase in the caudal nidopallium of different experimental groups.

(**A**) The locations of different subdivisions of the caudal nidopallium in house crows are demonstrated in a coronal section from a Trained bird stained for TH (Sen, Parishar et al., 2019). Scale bar, 1mm. (**B**) Quantification of the area covered by TH profiles revealed that there were no significant differences in staining for TH in dNC, iNC, mNCL, and vNC across different groups of house crow. In lNCL, TH expression was higher in Baseline controls compared to that in Undertrained birds. (**C**) Representative images from different TH-positive regions of NC. In the dNC and mNCL, a dense plexus of intensely stained TH-positive profiles is observed with a few baskets. The iNC comprises of numerous TH positive baskets and can be easily distinguished from other divisions of NC due to the presence of fibers connecting AId to NCL. The lNCL is characterized by very prominent TH-positive baskets but the distribution of TH fibers is not as dense as that in mNCL. The ventral part of NC (vNC) can be delineated from the adjacent lNCL and iNC by the presence of sparsely distributed TH-labelled fibers and TH-positive baskets **, P < 0.01. N = 4 data points for Baseline, Trained and No-Association birds; N = 6 for Undertrained birds.

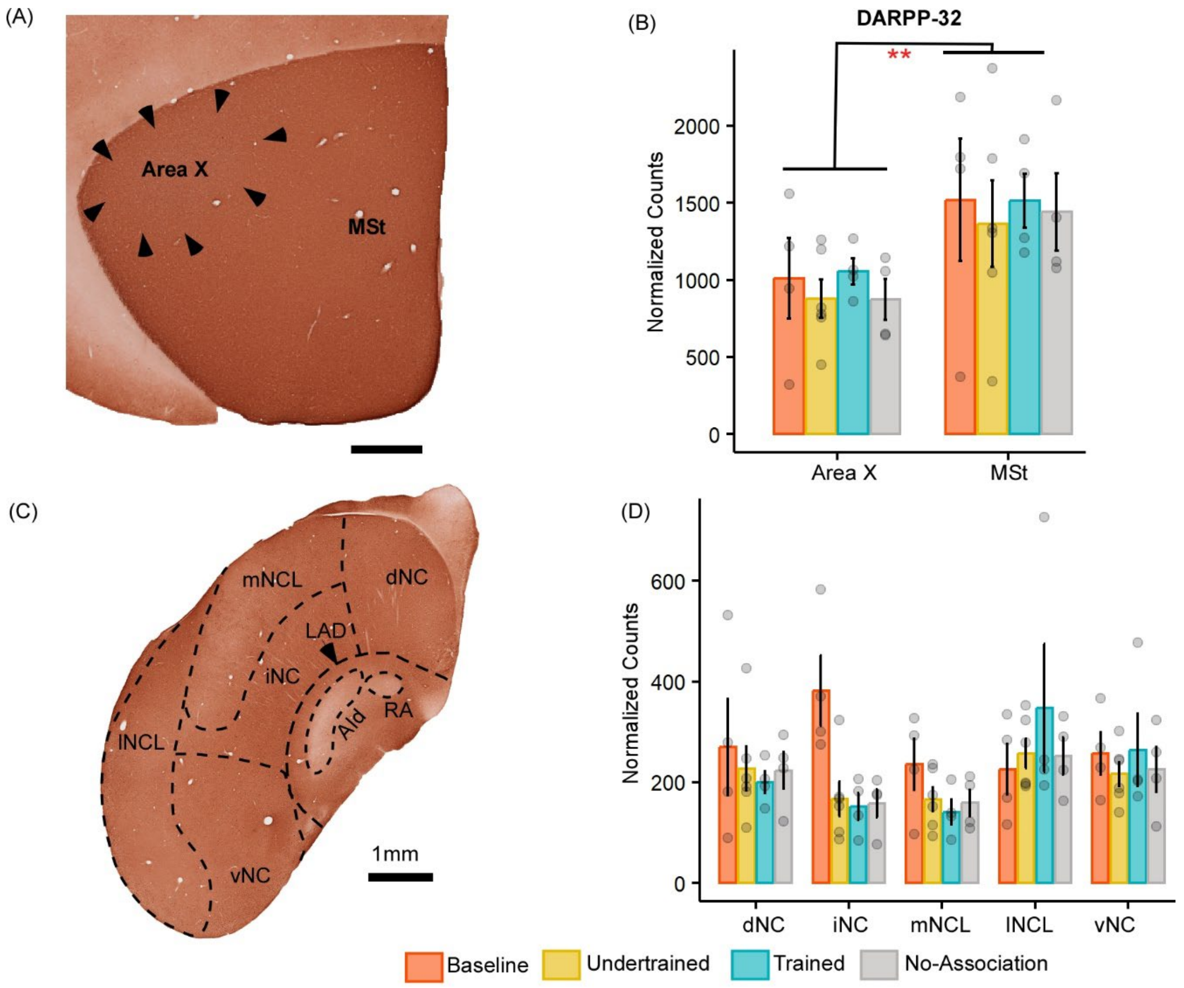

Supplementary Figure 7. DARPP-32 expression in different groups of house crows.

(**A**) A DARPP-32-stained coronal section demonstrates that Area X can be discerned from the surrounding MSt, since staining intensity is slightly lower and it has fewer DARPP-positive cells. (**B**) There were no significant differences in overall counts (mean ± SEM) of DARPP-positive neurons in Area X or MSt in any of the experimental groups. (**C**) A DARPP-32-stained section at the level of the caudal nidopallium demonstrating that RA and AId were characteristically devoid of label (cf. Sen, Parishar et al., 2019). (**D**) There were no significant differences in the number of DARPP-32 neurons (mean ± SEM) in any of the subdivisions of NC in the four experimental groups of house crows. Scale bar, 1mm; **, $P < 0.01$. N = 4 data points for Baseline, Trained and No-Association birds; N = 6 for Undertrained birds.

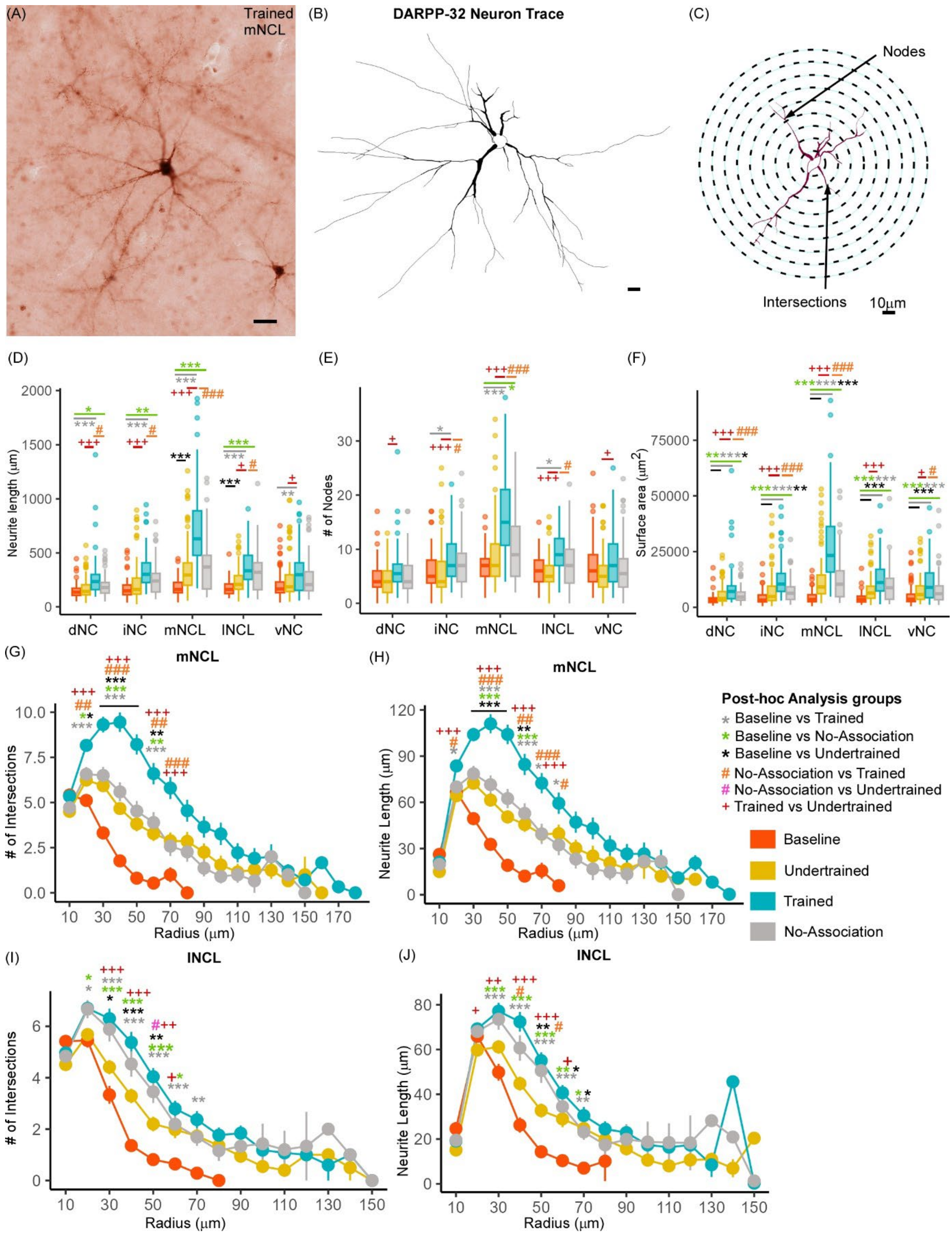

Supplementary Figure 8. A comparison of DARPP-32 neurons reconstructed in three dimensions.

(**A**) An example of a DARPP-32-stained neuron from the mNCL region of the Trained group and its (**B**) three-dimensional reconstruction. (**C**) The contour of a DARPP-32 neuron superimposed on concentric Sholl shells. Arrow indicates the nodes and point of intersections with the Sholl radii. Scale bar, 10µm. We observed a statistically significant increase in the (**D**) neurite length, and (**E**) number

of nodes, which indicate an increase in neurite branching in Trained house crows compared to those in Undertrained, No-Association and Baseline birds in all five subdivisions of NC. (**F**) An increase in the neurite field (measured by 3D surface area) was seen in the Trained group compared to the Undertrained, No-Association and Baseline groups in different divisions of NC. A Sholl analysis demonstrated an increase in the number of intersections and neurite length (mean ± SEM) between the Sholl radii 20-70 μm in the Trained group compared to that in Baseline, Undertrained and No-Association groups in (**G** and **H**) mNCL, (**I** and **J**) lNCL. */#/+ $P<0.05$; **/##/++ $P<0.01$; ***/###/+++ $P<0.001$. N = 60 data points for Baseline, Trained and No-Association birds; N = 90 for Undertrained birds.

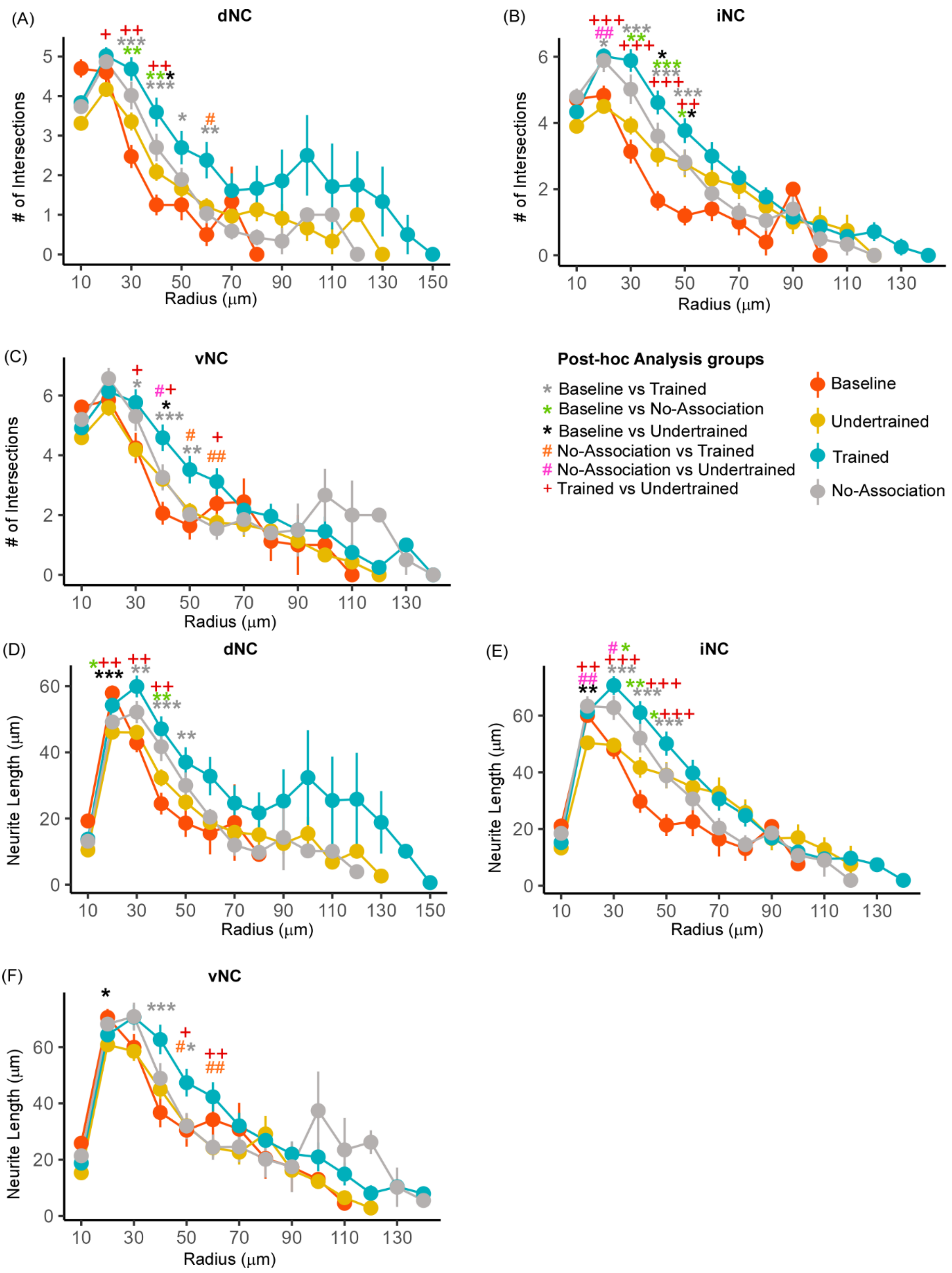

Supplementary Figure 9. Sholl analysis of neurite branching and neurite length in DARPP-32 neurons of NC.

An increase in the number of intersections (mean ± SEM) was observed between the Sholl radii 20-70 µm in the Trained group in (**A**) dNC, (**B**) iNC, and (**C**) vNC. In the three subdivisions of NC, the

highest number of intersections made by DARPP-32-labeled neurites with Sholl radii were in Trained birds versus that in Undertrained, No-Association and Baseline house crows. At a few points, the number of intersections was significantly higher in the No-Association and Undertrained groups versus that in Baseline controls. Similar changes were observed for neurite lengths in (**D**) dNC, (**E**) iNC, and (**F**) vNC. */#/+ $P<0.05$; **/##/++ $P<0.01$; ***/###/+++ $P<0.001$. N = 60 data points for Baseline, Trained and No-Association birds; N = 90 for Undertrained birds.

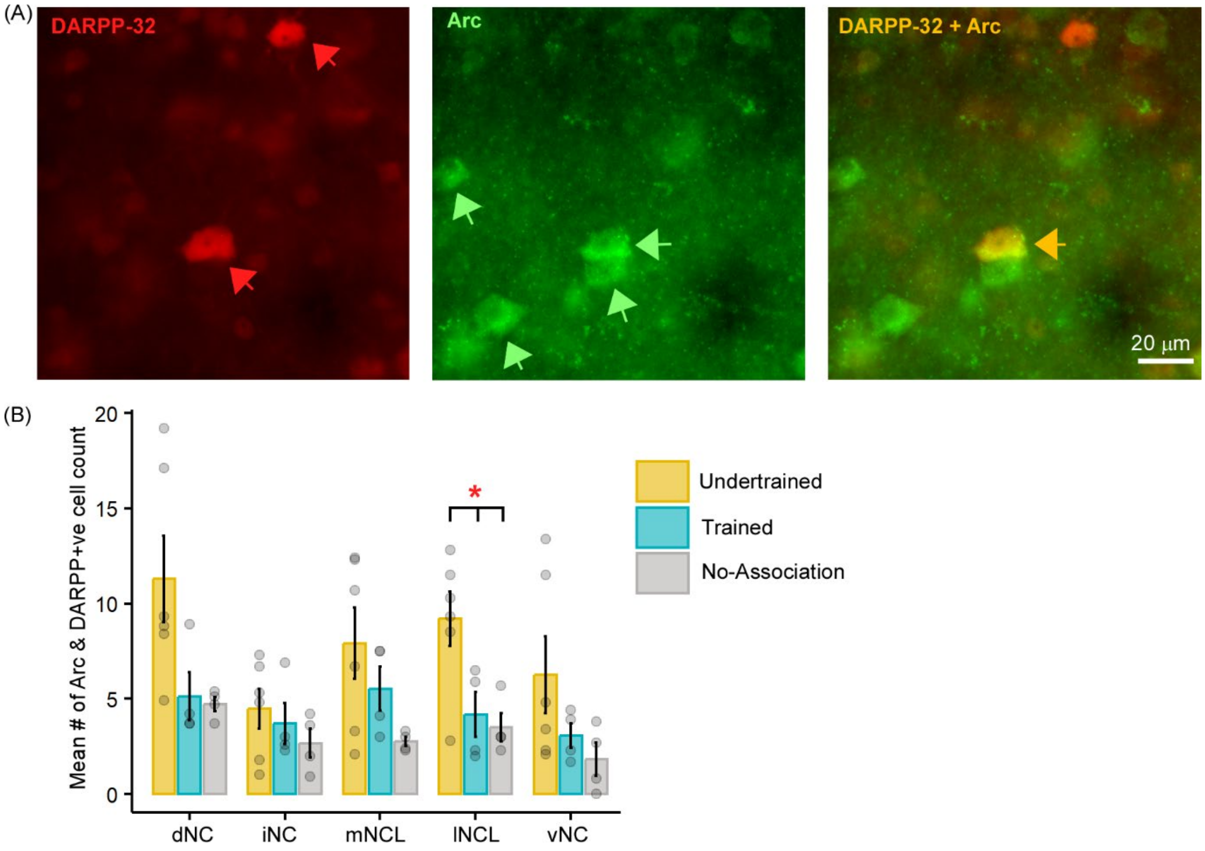

Supplementary Figure 10. Double label for Arc and DARPP-32 in NC.

(**A**) Representative images of Arc and DARPP-32 double-label from the lNCL region of an Undertrained bird demonstrating neurons positive for DARPP-32 (*red arrows*), Arc (*green arrows*), and for both (*orange arrows*). (**B**) Counts of neurons double-labeled for Arc and DARPP-32 (mean ± SEM) in NC demonstrated a significantly high number of double-labeled neurons in lNCL of Undertrained birds versus those in the Trained and No-Association groups. Scale bar, 20µm. *, $P < 0.05$. N = 4 data points for Trained and No-Association birds; N = 6 for Undertrained birds.

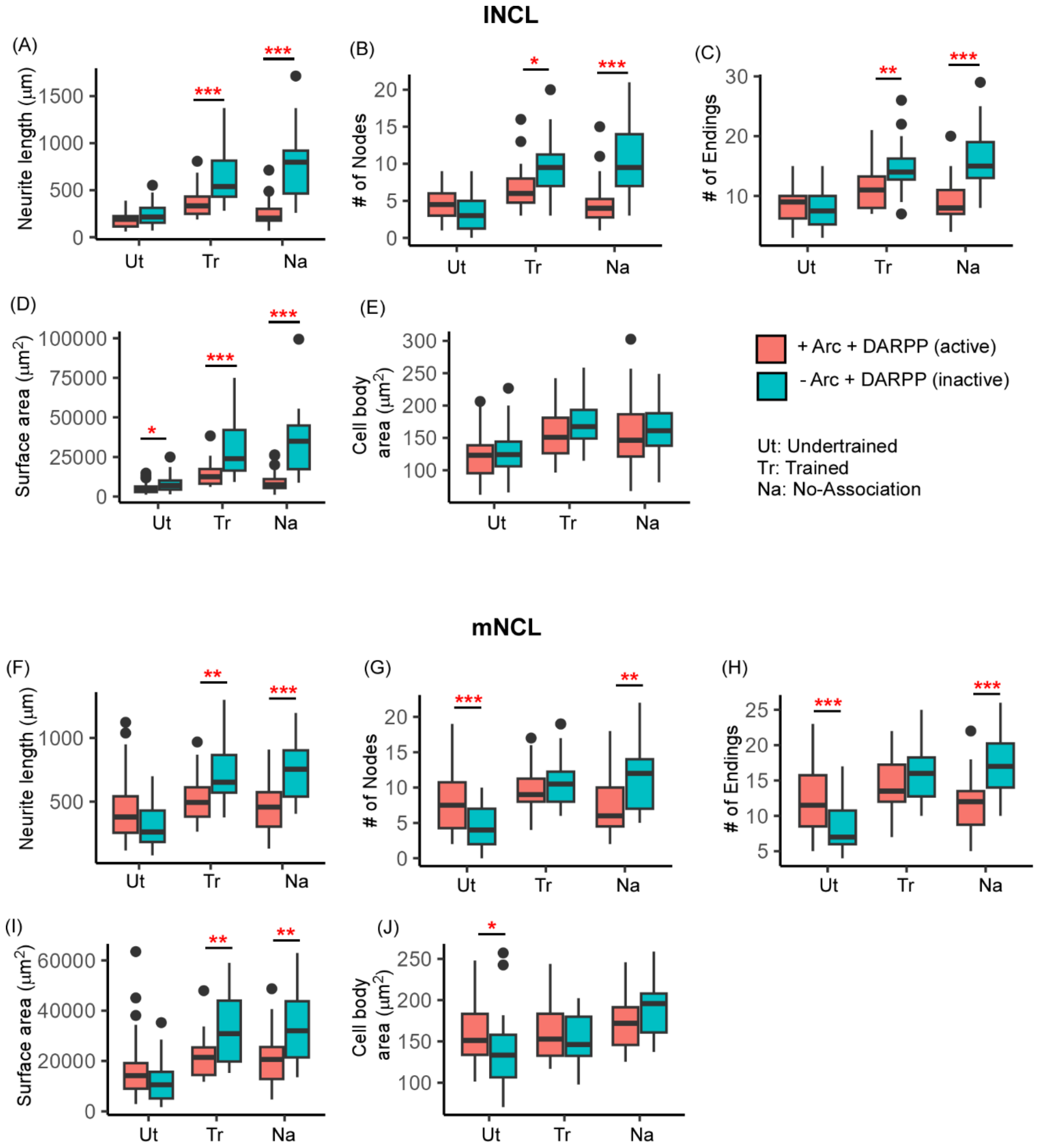

Supplementary Figure 11. A comparison of active and inactive DARPP-32-positive neurons in NCL.

In lNCL, the (**A**) neurite length, the number of (**B**) nodes and (**C**) endings were significantly greater in inactive DARPP-32-positive neurons (blue boxplots) compared to active neurons (orange boxplots) of Trained and No-Association birds. (**D**) In all three groups, the area of the neurite field was also higher in inactive versus active neurons. However, (**E**) the area of somata was similar across both sets of neurons in all groups. (**F**) The neurite length of the inactive neurons was significantly greater than that of active neurons in mNCL of the Trained and No-Association groups. However, active neurons in the Undertrained group had significantly more (**G**) nodes and (**H**) endings. In the No-Association group, inactive mNCL neurons were significantly more branched versus active ones. (**I**) The area of

neurite fields was significantly greater in inactive mNCL neurons in Trained and No-Association birds versus that in active neurons, which was similar to that in lNCL. (**J**) In mNCL, active neurons of the Undertrained group had larger somata compared to inactive ones. * $P<0.05$; ** $P<0.01$; *** $P<0.001$. N = 20 data points for Trained and No-Association birds; N = 30 for Undertrained birds.

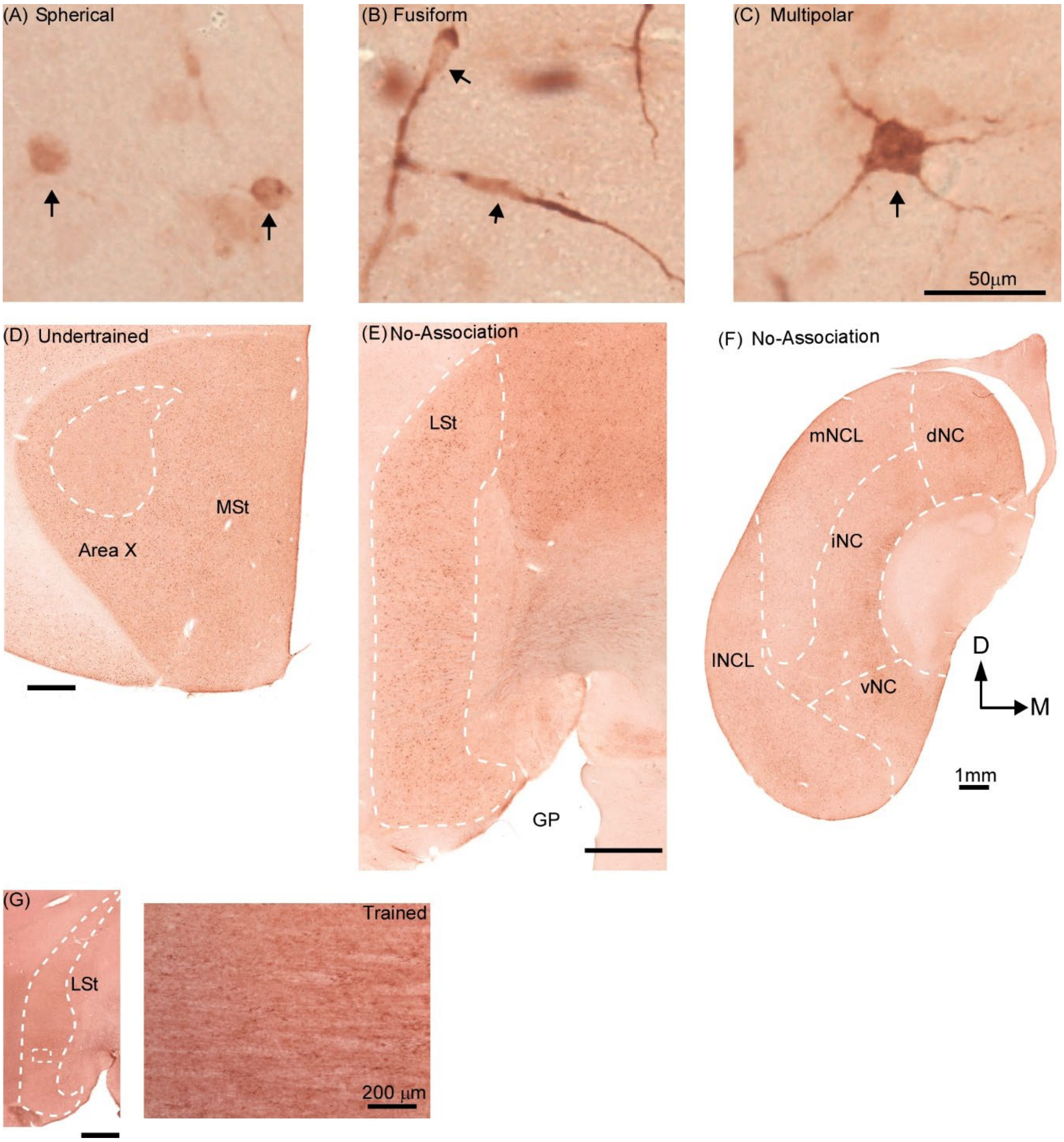

Supplementary Figure 12. Doublecortin (DCX) expression in different regions of the house crow brain.

High magnification images of DCX neurons demonstrating differences in their morphology including (**A**) spherical, (**B**) fusiform and (**C**) multipolar neurons (*arrows*). Scale bar, 50 μm. (**D**) In the coronal plane, Area X can be delineated from the surrounding MSt due to lower levels of staining for DCX. (**E**) The boundaries of LSt were clearly demarcated due to the presence of a larger number of DCX-positive neurons in this region compared to that in the adjoining GP. (**F**) Staining for DCX demonstrated that there was a higher density of immature neurons in NC compared to that in the arcopallium. Scale bar, 1mm. (**G**) An image of a negative control was acquired by staining a section at the level of LSt from a Trained bird by following all steps for immunohistochemistry but omitting

incubation in the primary antibody solution against DCX. The *inset* on the right demonstrates the lack of staining within LSt at high power. Scale bars, 1mm; 200µm.

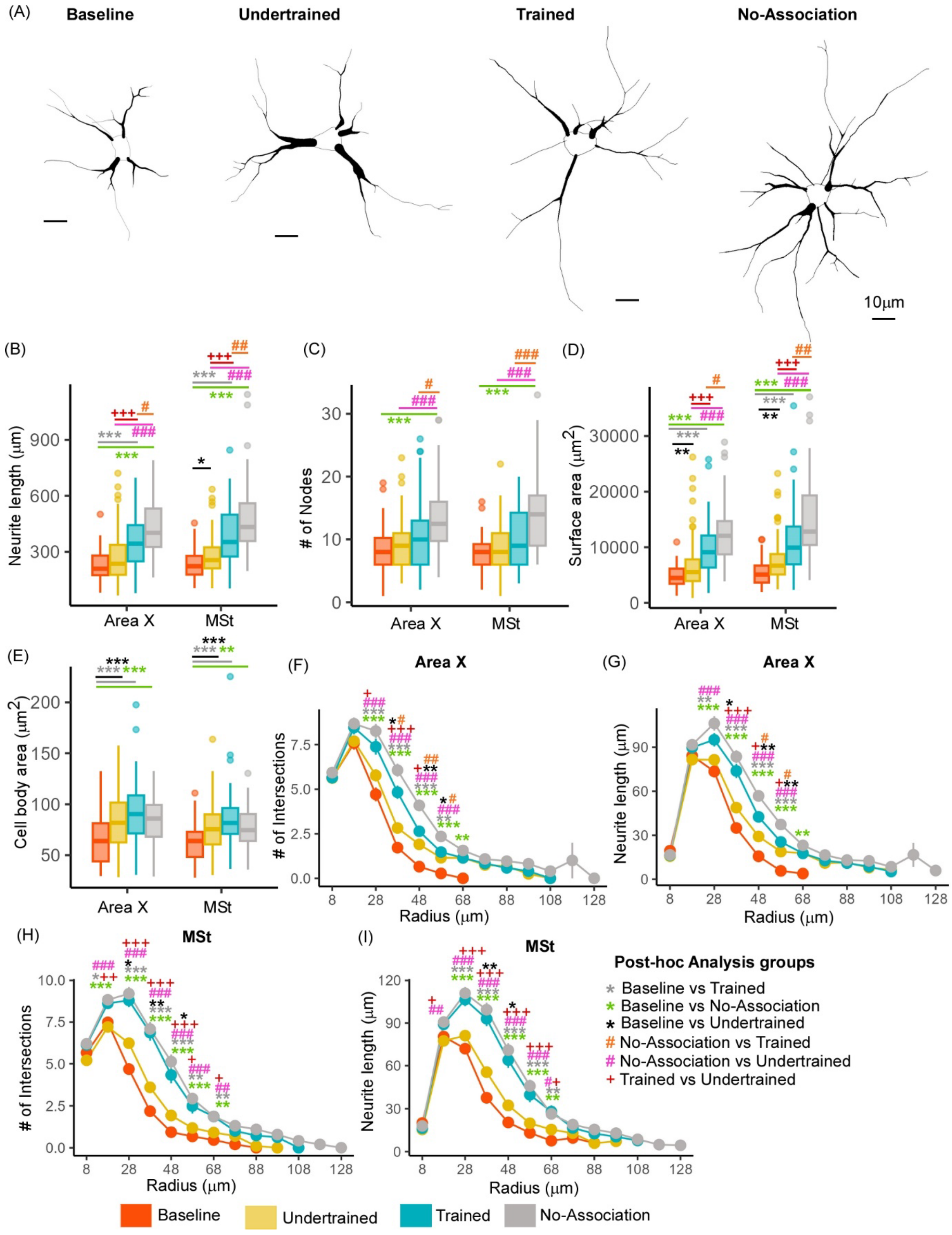

Supplementary Figure 13. A comparison of structural changes in DCX-positive neurons in Area X and MSt.

(**A**) Reconstructions of the three-dimensional structure of DCX-positive neurons demonstrating that they were the most complex in terms of the number and branching of dendrites in the No-Association group. Scale bar, 10µm. Comparisons of (**B**) neurite length, (**C**) number of nodes, (**D**) neurite field

area measured by performing a convex hull analysis, and (**E**) area of somata were analyzed to study changes in the structure of DCX-positive neurons induced by learning and decision-making. The neurite length of these neurons was significantly greater in Trained and No-Association birds compared to that in Baseline and Undertrained birds. Neurite length of DCX-labeled neurons was the greatest in the No-Association group, followed by that in the Trained group. Similar results were observed for the area of the neurite field. For the number of nodes, significant differences were only observed when comparisons were made between the No-Association versus Trained, Baseline, and Undertrained groups. Somata of DCX-positive neurons in the Trained, Undertrained, and No-Association groups were larger in area compared to those in the Baseline group. The (**F**) number of intersections and (**G**) neurite length of DCX-positive neurons was compared across different behavioral groups in Area X. We found that neurite branching of DCX-positive neurons in No-Association and Trained birds was significantly greater compared to that in Undertrained and Baseline control groups. The largest changes were observed in these parameters in the No-Association group followed by those in the Trained and Undertrained groups. (**H**) The number of intersections and (**I**) neurite length in DCX-positive neurons traced from MSt were significantly higher in the No-Association and Trained groups versus other groups. However, there were no differences in these parameters when the Trained and No-Association groups were compared. */+/#, P<0.05; **/++/##, P<0.01; ***/+++/###, P<0.001. N = 60 data points for Baseline, Trained and No-Association birds; N = 90 for Undertrained birds.

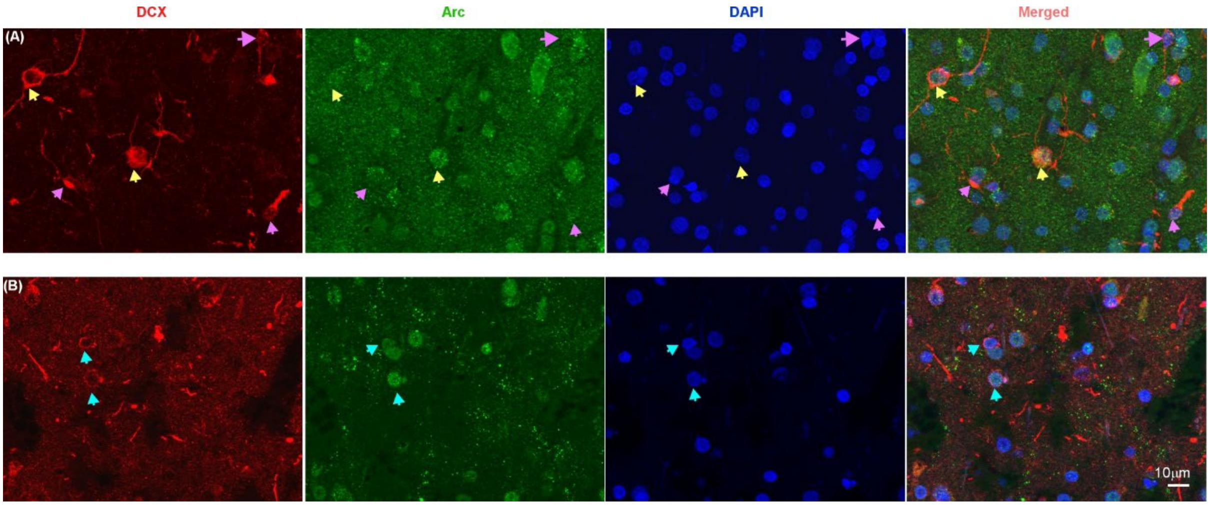

Supplementary Figure 14. Double-labeled Arc and DCX neurons in MSt.

Selected images are shown from the same z-stack but at different planes from MSt in an Undertrained bird. (**A**) In the upper panel, multipolar and fusiform neurons are labeled with DCX (red), Arc (green), the nuclear marker DAPI (blue) and the merged image (last column). Neurons labeled with DCX are indicated by *yellow arrows*, whereas Arc-positive neurons are indicated by *pink arrows*. The *yellow arrow* in the center of the image demonstrates a DCX- and Arc- co-labeled multipolar neuron, whereas in the image on the top left, a *yellow arrow* indicates an inactive (Arc-negative) DCX-positive multipolar neuron. The merged image clearly demonstrates that fusiform cells (*pink arrows*) are devoid of the Arc label. (**B**) In the bottom panel, *blue arrows* in images from left to right showing staining for DCX (red), Arc (green), and DAPI (blue), and in the merged image (last column) indicate the presence of active spherical neurons which are devoid of processes in MSt. Scale bar: 10 μm.

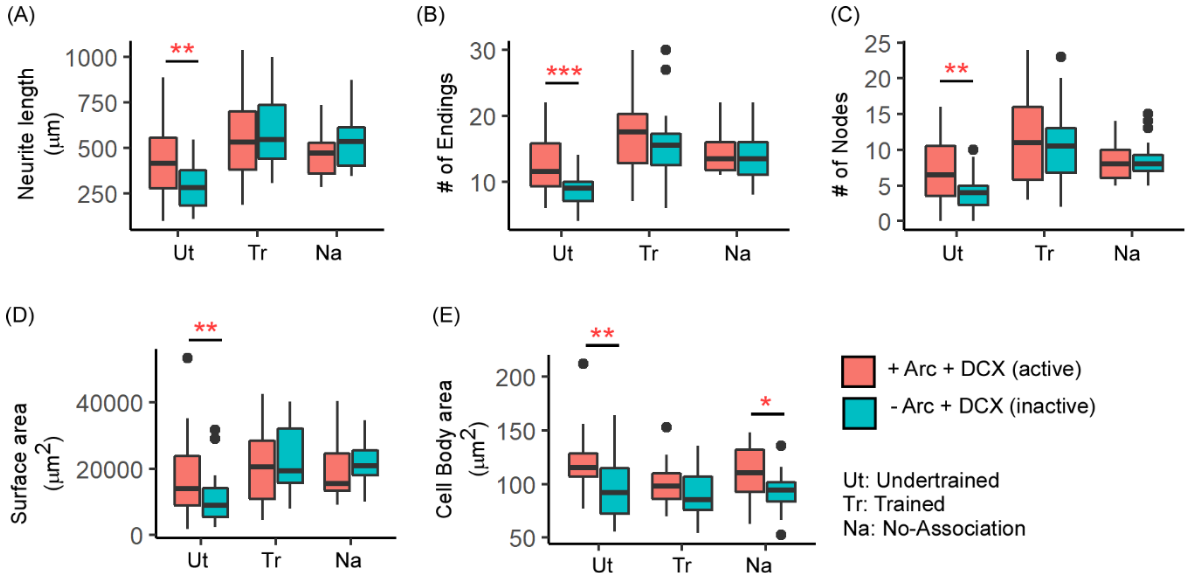

Supplementary Figure 15. A comparison of the complexity of active and inactive DCX neurons in MSt.

The (**A**) length of neurites was significantly greater in active DCX-positive neurons versus inactive ones in the Undertrained group in MSt. In the same group, the (**B**) number of endings and (**C**) number of nodes was also greater in active DCX-labeled neurons compared to those which were inactive. (**D**) Active DCX-positive neurons in Undertrained birds additionally demonstrated an expansion in the area of neurites versus inactive Arc-negative DCX-positive neurons. (**E**) The area of somata of active DCX-labeled neurons in the Undertrained and No-Association birds, compared to this measure for inactive neurons. *, $P < 0.05$; **, $P < 0.01$; ***, $P < 0.001$. N = 20 data points for Trained and No-Association birds; N= 30 for Undertrained birds.

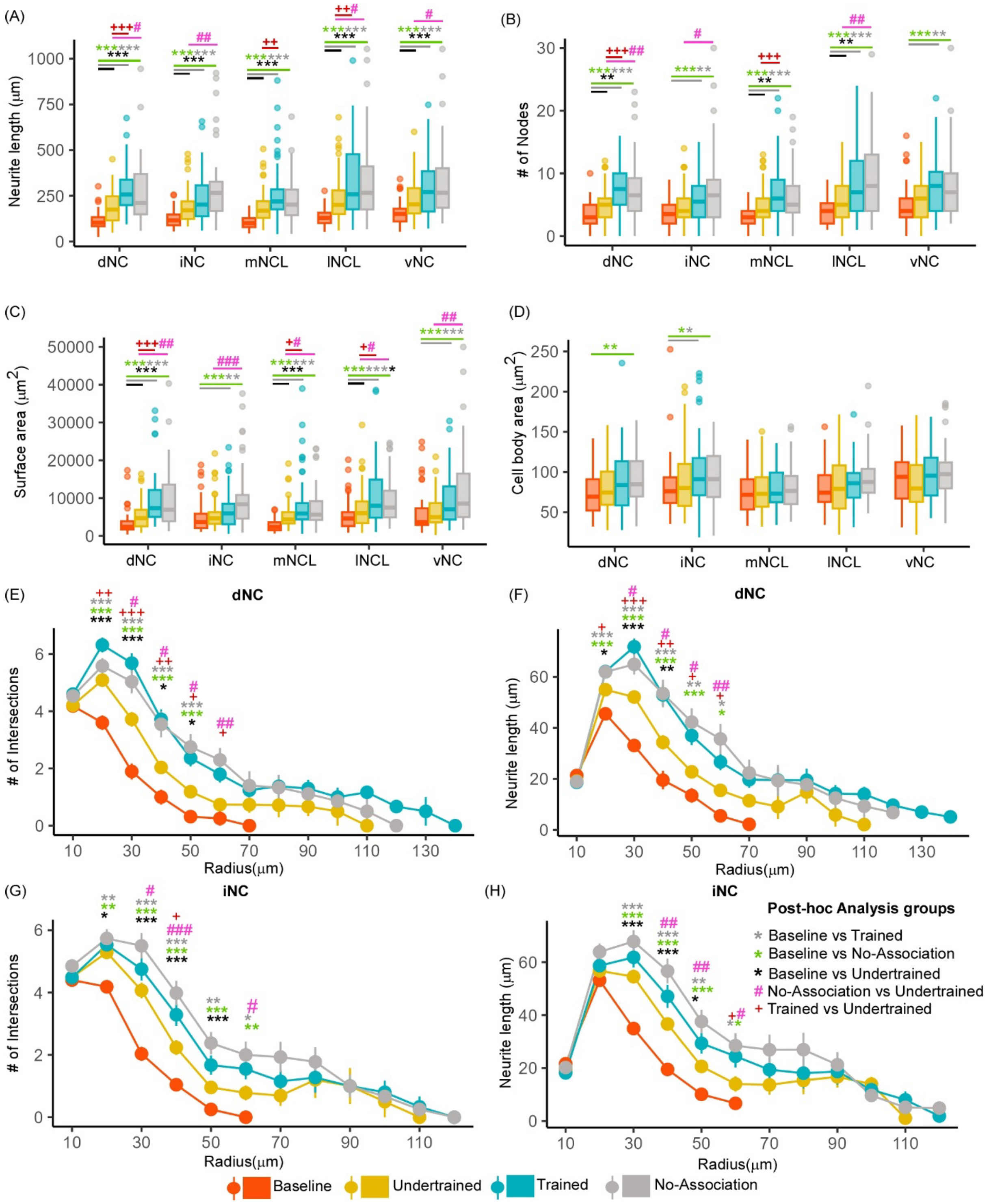

Supplementary Figure 16. Structural changes in multipolar DCX-positive neurons in NC.

The (**A**) neurite length, (**B**) number of nodes and (**C**) neurite field area was significantly greater in Undertrained, Trained and No-Association groups compared to that in Baseline controls in all subdivisions of NC. Furthermore, these parameters were significantly greater in NC of Trained and No-Association birds versus that in Undertrained birds. (**D**) The area of somata was significantly greater in dNC and iNC of No-Association birds versus that in Baseline controls. A Sholl analysis

demonstrated that the number of intersections and neurite length were significantly greater in Trained and No-Association birds versus that in Baseline and Undertrained crows in (**E** and **F**) dNC and (**G** and **H**) iNC. */+/#, $P<0.05$; **/++/##, $P<0.01$; ***/+++/###, $P<0.001$. N = 60 data points for Baseline, Trained and No-Association birds; N= 90 for Undertrained birds.

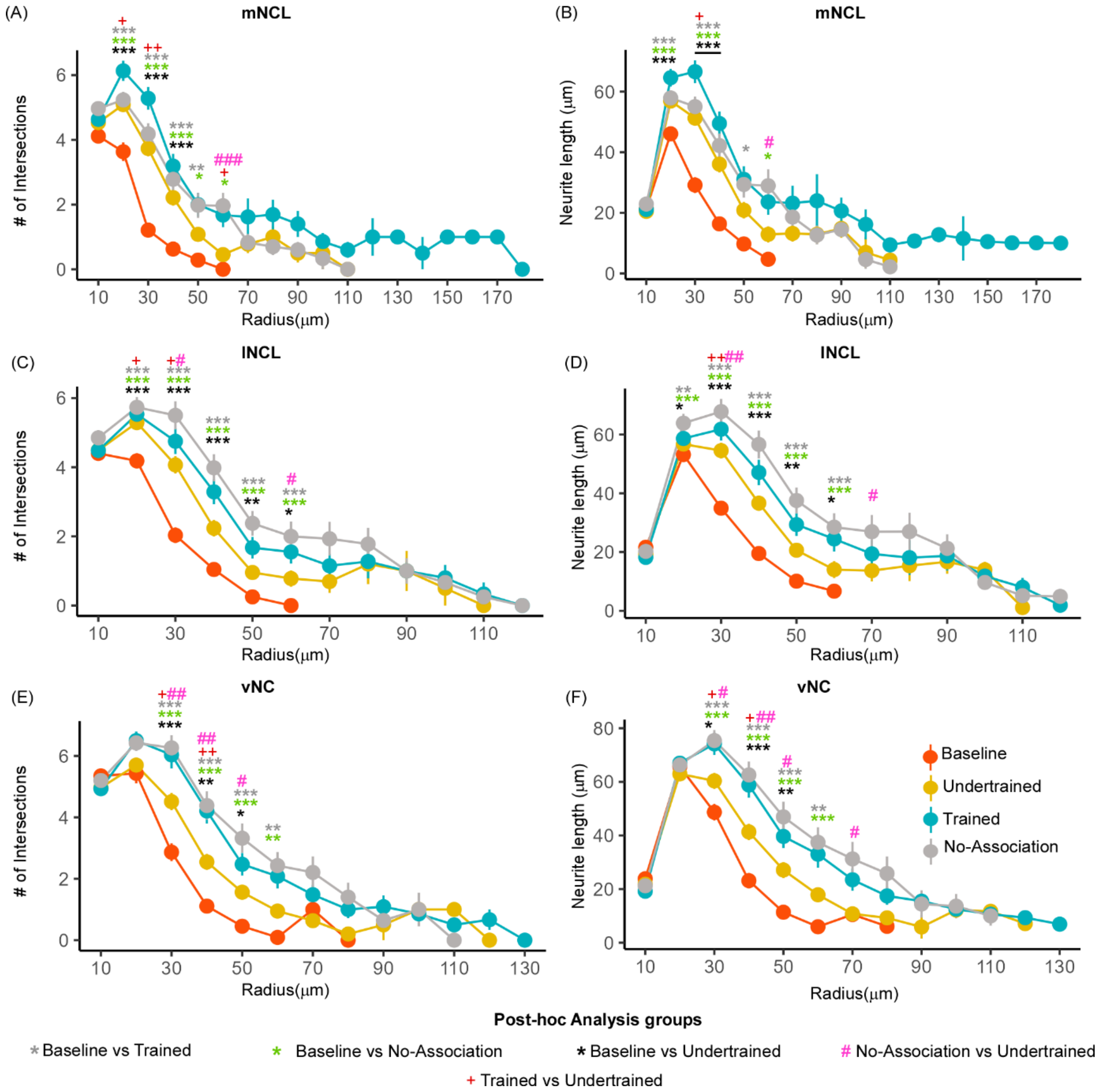

Supplementary Figure 17. A Sholl analysis of DCX-positive multipolar neurons in different subdivisions of NC.

The number of intersections and neurite length were significantly greater in DCX-labeled neurons in the Trained and No-Association groups versus Baseline and Undertrained groups in (**A** and **B**) mNCL, (**C** and **D**) lNCL, and (**E** and **F**) vNC. */+/#, P<0.05; **/++/##, P<0.01; ***/+++/###, P<0.001. N = 60 data points for Baseline, Trained and No-Association birds; N= 90 for Undertrained birds.

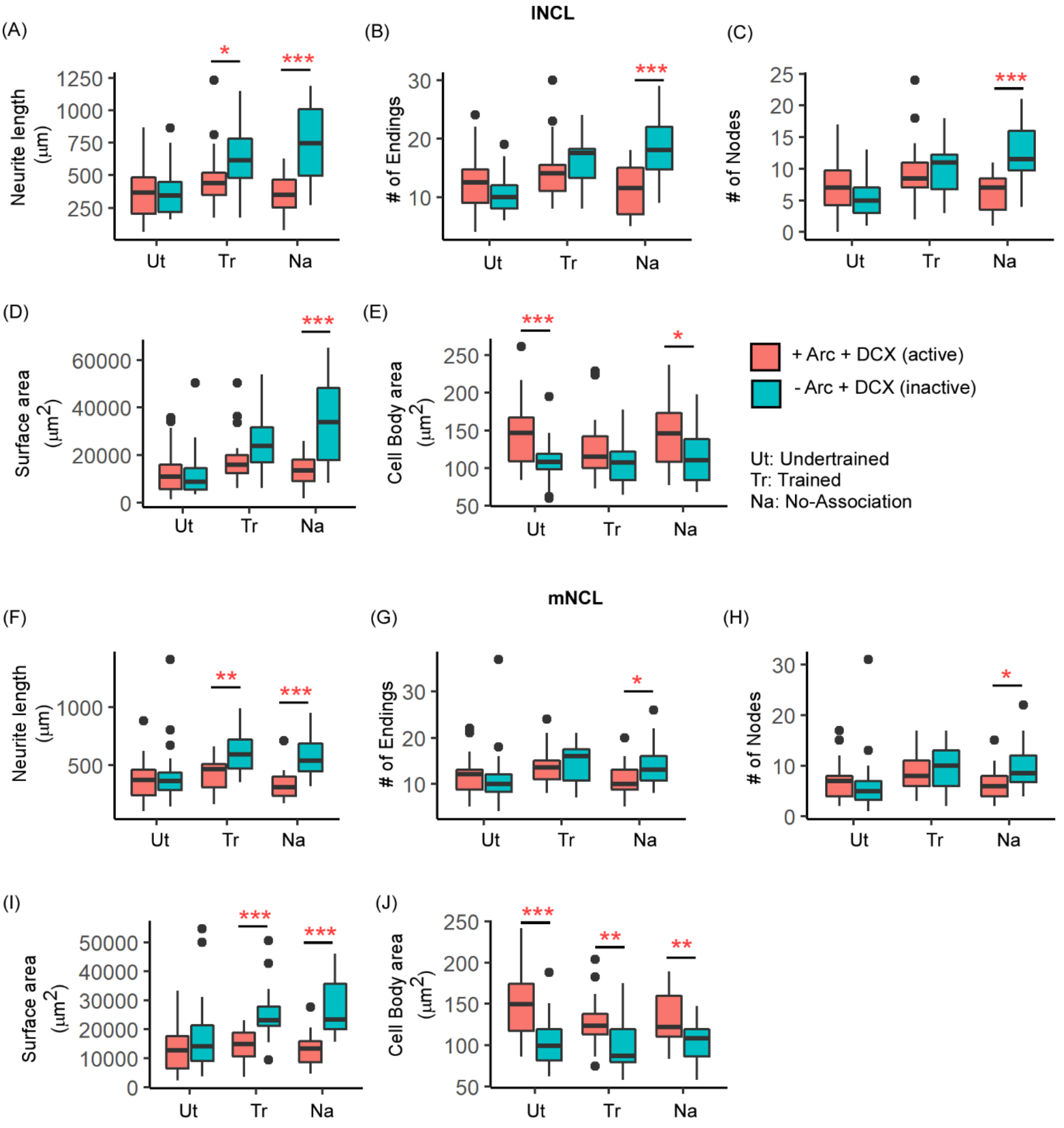

Supplementary Figure 18. Comparison of active and inactive DCX-positive neurons in mNCL and lNCL.

The (**A**) length of neurites of inactive DCX-positive neurons was significantly greater in the Trained and No-Association groups compared to active DCX-positive neurons in lNCL. The (**B**) number of endings, (**C**) number of nodes and (**D**) the area of the neurite field was significantly greater only in inactive DCX-positive neurons of the No-Association group. The (**E**) area of the somata was significantly greater in the active versus inactive population of DCX-labeled neurons in the Undertrained and No-Association groups. Similar changes in (**F**) neurite length, (**G**) number of endings and (**H**) number of nodes were observed in the reconstructed active and inactive DCX neurons in mNCL. (**I**) The area of the neurite field was greater in inactive DCX-labeled neurons in

mNCL of the Trained and No-Association groups compared to active DCX-positive neurons. (**J**) In all three experimental groups, the size of the somata was greater in active DCX-labeled neurons compared to inactive neurons. *, $P < 0.05$; **, $P < 0.01$; ***, $P < 0.001$. N = 20 data points for Trained and No-Association birds; N = 30 for Undertrained birds.
