# Supplementary material for: Changes in the dopaminergic circuitry and adult neurogenesis linked to reinforcement learning in corvids
Source: Front Neurosci. 2024 May 14;18:1359874. doi: 10.3389/fnins.2024.1359874 (PMC11130420; doi:10.3389/fnins.2024.1359874)
Supplement: Supplementary file 2 [file Data_Sheet_2.docx]

**Table S1.** Statistical details of DARPP-32 neuron summary analysis.

| **Area** | **Neuron Morphometry parameters** | **Kruskal-Wallis P value** | **χ2 value** | **df** | **Post hoc P value (Dunn’s)** |
| --- | --- | --- | --- | --- | --- |
| dNC | Neurite length | 2.73E-07 | 33.339 | 3 | Bl vs Na: 2.355e-02 |
|  |  |  |  |  | Bl vs Tr: 4.9580e-07 |
|  |  |  |  |  | Na vs Tr: 2.7498e-02 |
|  |  |  |  |  | Tr vs Ut: 2.0628e-05 |
|  | Endings | 0.0001449 | 20.331 | 3 | Tr vs Ut: 5.4015e-05 |
|  | Node | 3.55E-03 | 13.572 | 3 | Tr vs Ut: 0.0014 |
|  | Cell body area | 0.0001712 | 19.982 | 3 | Bl vs Na: 0.0013 |
|  |  |  |  |  | Bl vs Tr: 0.0242 |
|  |  |  |  |  | Bl vs Ut: 0.0001 |
|  | Neurite field area | 6.49E-08 | 36.295 | 3 | Bl vs Na: 2.5422e-03 |
|  |  |  |  |  | Bl vs Tr: 1.6625e-08 |
|  |  |  |  |  | Bl vs Ut: 1.6684e-02 |
|  |  |  |  |  | Na vs Tr: 2.2899e-02 |
|  |  |  |  |  | Tr vs Ut: 9.2214e-04 |
| iNC | Neurite length | 2.35E-09 | 43.097 | 3 | Bl vs Na: 4.8596e-03 |
|  |  |  |  |  | Bl vs Tr: 5.7186e-09 |
|  |  |  |  |  | Na vs Tr: 1.1865e-02 |
|  |  |  |  |  | Tr vs Ut: 1.4886e-06 |
|  | Endings | 0.0006685 | 17.117 | 3 | Tr vs Ut: 0.00038 |
|  | Nodes | 0.0001785 | 19.895 | 3 | Bl vs Tr: 0.0260 |
|  |  |  |  |  | Na vs Ut: 0.0405 |
|  |  |  |  |  | Tr vs Ut: 0.0001 |
|  | Cell body area | 1.48E-06 | 29.855 | 3 | Bl vs Na: 6.906e-03 |
|  |  |  |  |  | Bl vs Tr: 8.2909e-04 |
|  |  |  |  |  | Bl vs Ut: 4.6052e-07 |
|  | Neurite field area | 1.97E-12 | 57.537 | 3 | Bl vs Na: 7.4356e-04 |
|  |  |  |  |  | Bl vs Tr: 5.0211e-13 |
|  |  |  |  |  | Bl vs Ut: 4.6483e-03 |
|  |  |  |  |  | Na vs Tr: 5.8146e-04 |
|  |  |  |  |  | Tr vs Ut: 1.4372e-06 |
| mNCL | Neurite length | <2.2e-16 | 101.58 | 3 | Bl vs Na: 2.9169e-06 |
|  |  |  |  |  | Bl vs Tr: 5.6585e-23 |
|  |  |  |  |  | Bl vs Ut: 2.4537e-06 |
|  |  |  |  |  | Na vs Tr: 1.0405e-06 |
|  |  |  |  |  | Tr vs Ut: 3.7504e-09 |
|  | Endings | 6.98E-13 | 59.65 | 3 | Bl vs Na: 3.7367e-02 |
|  |  |  |  |  | Bl vs Tr: 1.2931e-12 |
|  |  |  |  |  | Na vs Tr: 5.1978e-06 |
|  |  |  |  |  | Tr vs Ut: 1.0520e-08 |
|  | Nodes | 9.40E-13 | 59.045 | 3 | Bl vs Na: 1.6916e-02 |
|  |  |  |  |  | Bl vs Tr: 2.8474e-12 |
|  |  |  |  |  | Na vs Tr: 3.2131e-05 |
|  |  |  |  |  | Tr vs Ut: 4.3118e-09 |
|  | Cell body area | 1.12E-11 | 54.004 | 3 | Bl vs Na: 4.2205e-04 |
|  |  |  |  |  | Bl vs Tr: 1.0789e-03 |
|  |  |  |  |  | Bl vs Ut: 1.3300e-12 |
|  |  |  |  |  | Na vs Ut: 4.9192e-03 |
|  |  |  |  |  | Tr vs Ut: 2.4721e-03 |
|  | Neurite field area | <2.2e-16 | 115.18 | 3 | Bl vs Na: 1.9548e-07 |
|  |  |  |  |  | Bl vs Tr: 4.3815e-26 |
|  |  |  |  |  | Bl vs Ut: 9.8288e-09 |
|  |  |  |  |  | Na vs Tr: 2.0009e-07 |
|  |  |  |  |  | Tr vs Ut: 3.4799e-08 |
| lNCL | Neurite length | 5.80E-11 | 50.652 | 3 | Bl vs Na: 2.5589e-05 |
|  |  |  |  |  | Bl vs Tr: 5.1715e-11 |
|  |  |  |  |  | Bl vs Ut: 8.4143e-03 |
|  |  |  |  |  | Na vs Tr: 4.6662e-02 |
|  |  |  |  |  | Na vs Ut: 4.4775e-02 |
|  |  |  |  |  | Tr vs Ut: 2.8331e-05 |
|  | Endings | 3.11E-07 | 33.071 | 3 | Bl vs Tr: 4.8049e-05 |
|  |  |  |  |  | Na vs Tr: 2.73350e-02 |
|  |  |  |  |  | Na vs Ut: 4.2754e-02 |
|  |  |  |  |  | Tr vs Ut: 3.7009e-07 |
|  | Nodes | 6.00E-08 | 36.457 | 3 | Bl vs Tr: 1.129886e-04 |
|  |  |  |  |  | Na vs Tr: 1.9903e-02 |
|  |  |  |  |  | Na vs Ut: 1.6804e-02 |
|  |  |  |  |  | Tr vs Ut: 3.0047e-08 |
|  | Cell body area | 6.24E-05 | 22.094 | 3 | Bl vs Na: 2.7019e-02 |
|  |  |  |  |  | Bl vs Tr: 1.3456e-02 |
|  |  |  |  |  | Bl vs Ut: 1.8031e-05 |
|  | Neurite field area | 1.11E-12 | 58.715 | 3 | Bl vs Na: 6.8181e-07 |
|  |  |  |  |  | Bl vs Tr: 5.7865e-13 |
|  |  |  |  |  | Bl vs Ut: 3.9893e-05 |
|  |  |  |  |  | Tr vs Ut: 5.5516e-04 |
| vNC | Neurite length | 0.005778 | 12.528 | 3 | Bl vs Tr: 0.0055 |
|  |  |  |  |  | Tr vs Ut: 0.0356 |
|  | Endings | 0.0233 | 9.5029 | 3 | Tr vs Ut: 0.0240 |
|  | Node | 0.01441 | 10.552 | 3 | Tr vs Ut: 0.0150 |
|  | Cell body area | 1.48E-06 | 29.855 | 3 | Bl vs Na: 4.1177e-02 |
|  |  |  |  |  | Bl vs Tr: 1.5321e-02 |
|  |  |  |  |  | Bl vs Ut: 5.4914e-07 |
|  |  |  |  |  | Na vs Ut: 3.2848e-02 |
|  | Neurite field area | 3.09E-05 | 23.556 |  | Bl vs Na: 4.9593e-02 |
|  |  |  |  |  | Bl vs Tr: 7.4806e-06 |
|  |  |  |  |  | Bl vs Ut: 2.2047e-02 |
|  |  |  |  |  | Na vs Tr: 3.7804e-02 |
|  |  |  |  |  | Tr vs Ut: 4.1275e-02 |

**Table S2.** Statistical details of Sholl analysis of DARPP-32 positive neurons.

| **Area** | **Radius (µm)** | **Intersections (KW P-Value)** | **χ2 value** | **df** | **Intersections (Dunn’s value)** | **Neurite length (KW P-Value)** | **χ2 value** | **df** | **Neurite length (Dunn’s P value)** |
| --- | --- | --- | --- | --- | --- | --- | --- | --- | --- |
| dNC | 20 | 0.0269 | 9.18 | 3 | Tr vs Ut: 0.0322 | 0.00038 | 18.26 | 3 | Bl vs Na: 0.0287 |
|  |  |  |  |  |  |  |  |  | Bl vs Ut: 0.0004 |
|  |  |  |  |  |  |  |  |  | Tr vs Ut: 0.0357 |
|  | 30 | 1.84E-06 | 29.40 | 3 | Bl vs Na: 3.0374e-03 | 0.00165 | 15.20 | 3 | Bl vs Tr: 0.0032 |
|  |  |  |  |  | Bl vs Tr: 9.4381e-07 |  |  |  | Tr vs Ut: 0.0063 |
|  |  |  |  |  | Tr vs Ut: 2.7109e-03 |  |  |  |  |
|  | 40 | 4.75E-07 | 32.20 | 3 | Bl vs Na: 2.2596e-03 | 3.38E-05 | 23.37 | 3 | Bl vs Na: 7.0483e-03 |
|  |  |  |  |  | Bl vs Tr: 2.1773e-07 |  |  |  | Bl vs Tr: 3.2721e-05 |
|  |  |  |  |  | Bl vs Ut: 3.0638e-02 |  |  |  | Tr vs Ut: 5.7953e-03 |
|  |  |  |  |  | Tr vs Ut: 1.1945e-03 |  |  |  |  |
|  | 50 | 0.02413 | 9.43 | 3 | Bl vs Tr: 0.0236 | 0.01042 | 11.26 | 3 | Bl vs Tr: 0.0092 |
|  | 60 | 0.002 | 14.76 | 3 | Bl vs Tr: 0.0056 | 0.02791 | 9.11 | 3 | - |
|  |  |  |  |  | Na vs Tr: 0.0337 |  |  |  |  |
| iNC | 20 | 2.58E-05 | 23.93 | 3 | Bl vs Tr: 1.1176e-02 | 0.00026 | 19.04 | 3 | Bl vs Ut: 0.0043 |
|  |  |  |  |  | Na vs Ut: 8.6244e-03 |  |  |  | Na vs Ut: 0.0070 |
|  |  |  |  |  | Tr vs Ut: 3.0370e-05 |  |  |  | Tr vs Ut: 0.0017 |
|  | 30 | 2.34E-07 | 33.66 | 3 | Bl vs Na: 1.7962e-03 | 1.46E-06 | 29.89 | 3 | Bl vs Na: 2.9255e-02 |
|  |  |  |  |  | Bl vs Tr: 3.1773e-07 |  |  |  | Bl vs Tr: 4.5964e-05 |
|  |  |  |  |  | Tr vs Ut: 2.1951e-04 |  |  |  | Na vs Ut: 2.6668e-02 |
|  |  |  |  |  |  |  |  |  | Tr vs Ut: 1.2260e-05 |
|  | 40 | 1.93E-08 | 38.78 | 3 | Bl vs Na: 5.0004e-04 | 2.25E-07 | 33.74 | 3 | Bl vs Na: 1.4663e-03 |
|  |  |  |  |  | Bl vs Tr: 1.1133e-08 |  |  |  | Bl vs Tr: 2.0916e-07 |
|  |  |  |  |  | Bl vs Ut: 2.2282e-02 |  |  |  | Tr vs Ut: 4.0450e-04 |
|  |  |  |  |  | Tr vs Ut: 2.8742e-04 |  |  |  |  |
|  | 50 | 1.78E-05 | 24.70 | 3 | Bl vs Na: 1.1158e-02 | 6.16E-05 | 22.12 | 3 | Bl vs Na: 2.5730e-02 |
|  |  |  |  |  | Bl vs Tr: 8.8748e-06 |  |  |  | Bl vs Tr: 2.9233e-05 |
|  |  |  |  |  | Bl vs Ut: 4.9909e-02 |  |  |  | Tr vs Ut: 1.4129e-02 |
|  |  |  |  |  | Tr vs Ut: 7.8737e-03 |  |  |  |  |
| vNC | 20 |  |  |  | - | 0.03128 | 8.86 | 3 | Bl vs Ut: 0.0249 |
|  | 30 | 0.0108 | 11.17 | 3 | Tr vs Ut: 0.0455 | 0.03872 | 8.38 | 3 | - |
|  | 40 | 2.96E-05 | 23.65 | 3 | Bl vs Tr: 7.7687e-06 | 0.00051 | 17.69 | 3 | Bl vs Tr: 0.0001 |
|  |  |  |  |  | Bl vs Ut: 1.4937e-02 |  |  |  |  |
|  |  |  |  |  | Na vs Tr: 3.9034e-02 |  |  |  |  |
|  |  |  |  |  | Tr vs Ut: 3.9634e-02 |  |  |  |  |
|  | 50 | 0.001999 | 14.80 | 3 | Bl vs Tr: 0.0022 | 0.007796 | 11.88 | 3 | Bl vs Tr: 0.0280 |
|  |  |  |  |  | Na vs Tr: 0.0297 |  |  |  | Na vs Tr: 0.0239 |
|  |  |  |  |  |  |  |  |  | Tr vs Ut: 0.0403 |
|  | 60 | 0.004441 | 13.09 | 3 | Na vs Tr: 0.0077 | 0.002419 | 14.39 | 3 | Na vs Tr: 0.0059 |
|  |  |  |  |  | Tr vs Ut: 0.0148 |  |  |  | Tr vs Ut: 0.0052 |
|  |  |  |  |  |  |  |  |  |  |
| mNCL | 20 | 2.81E-08 | 38.01 | 3 | Bl vs Na: 4.1674e-02 | 0.000534 | 17.59 | 3 | Bl vs Tr: 0.0151 |
|  |  |  |  |  | Bl vs Tr: 7.8858e-09 |  |  |  | Na vs Tr: 0.0154 |
|  |  |  |  |  | Bl vs Ut: 3.0420e-02 |  |  |  | Tr vs Ut: 0.0002 |
|  |  |  |  |  | Na vs Tr: 1.2458e-03 |  |  |  |  |
|  |  |  |  |  | Tr vs Ut: 1.2365e-04 |  |  |  |  |
|  | 30 | < 2.2e-16 | 79.83 | 3 | Bl vs Na: 2.6309e-06 | 4.03E-14 | 65.45 | 3 | Bl vs Na: 1.0602e-04 |
|  |  |  |  |  | Bl vs Tr: 3.8931e-18 |  |  |  | Bl vs Tr: 4.9156e-15 |
|  |  |  |  |  | Bl vs Ut: 2.1389e-05 |  |  |  | Bl vs Ut: 3.0858e-04 |
|  |  |  |  |  | Na vs Tr: 1.8467e-04 |  |  |  | Na vs Tr: 2.3652e-04 |
|  |  |  |  |  | Tr vs Ut: 7.9668e-07 |  |  |  | Tr vs Ut: 3.9870e-06 |
|  | 40 | < 2.2e-16 | 96.31 | 3 | Bl vs Na: 2.2412e-07 | < 2.2e-16 | 82.25 | 3 | Bl vs Na: 4.2816e-06 |
|  |  |  |  |  | Bl vs Tr: 1.1804e-21 |  |  |  | Bl vs Tr: 1.5906e-18 |
|  |  |  |  |  | Bl vs Ut: 2.7063e-06 |  |  |  | Bl vs Ut: 4.3885e-05 |
|  |  |  |  |  | Na vs Tr: 1.8470e-05 |  |  |  | Na vs Tr: 4.8581e-05 |
|  |  |  |  |  | Tr vs Ut: 1.4012e-08 |  |  |  | Tr vs Ut: 6.6858e-08 |
|  | 50 | < 2.2e-16 | 80.26 | 3 | Bl vs Na: 5.5715e-06 | < 2.2e-16 | 78.56 | 3 | Bl vs Na: 6.0695e-06 |
|  |  |  |  |  | Bl vs Tr: 2.4181e-17 |  |  |  | Bl vs Tr: 1.3745e-16 |
|  |  |  |  |  | Bl vs Ut: 2.4742e-05 |  |  |  | Bl vs Ut: 1.3537e-04 |
|  |  |  |  |  | Na vs Tr: 2.8534e-05 |  |  |  | Na vs Tr: 7.5297e-05 |
|  |  |  |  |  | Tr vs Ut: 1.6873e-08 |  |  |  | Tr vs Ut: 4.4473e-09 |
|  | 60 | 5.20E-09 | 41.47 | 3 | Bl vs Na: 1.6285e-03 | 4.44E-09 | 41.79 | 3 | Bl vs Na: 8.1200e-04 |
|  |  |  |  |  | Bl vs Tr: 6.4245e-08 |  |  |  | Bl vs Tr: 3.1963e-08 |
|  |  |  |  |  | Bl vs Ut: 3.1659e-03 |  |  |  | Bl vs Ut: 2.4197e-03 |
|  |  |  |  |  | Na vs Tr: 2.9580e-03 |  |  |  | Na vs Tr: 2.8485e-03 |
|  |  |  |  |  | Tr vs Ut: 2.7759e-05 |  |  |  | Tr vs Ut: 4.9739e-05 |
|  | 70 | 9.16E-06 | 26.08 | 3 | Na vs Tr: 0.0001 | 6.84E-06 | 26.69 | 3 | Bl vs Tr: 3.2846e-02 |
|  |  |  |  |  | Tr vs Ut: 0.0001 |  |  |  | Na vs Tr: 5.1357e-04 |
|  |  |  |  |  |  |  |  |  | Tr vs Ut: 5.3509e-05 |
| lNCL | 20 | 0.002 | 14.79 | 3 | Bl vs Na: 0.0462 | 0.01482 | 10.49 | 3 | Tr vs Ut: 0.01731569 |
|  |  |  |  |  | Bl vs Tr: 0.0149 |  |  |  |  |
|  |  |  |  |  | Tr vs Ut: 0.0194 |  |  |  |  |
|  | 30 | 6.74E-08 | 36.22 | 3 | Bl vs Na: 8.4806e-05 | 2.47E-06 | 28.79 | 3 | Bl vs Na: 4.3451e-04 |
|  |  |  |  |  | Bl vs Tr: 2.0187e-07 |  |  |  | Bl vs Tr: 7.5684e-06 |
|  |  |  |  |  | Bl vs Ut: 3.7966e-02 |  |  |  | Tr vs Ut: 4.1220e-03 |
|  |  |  |  |  | Tr vs Ut: 8.5413e-04 |  |  |  |  |
|  | 40 | 5.88E-12 | 55.32 | 3 | Bl vs Na: 6.2025e-07 | 1.09E-11 | 54.06 | 3 | Bl vs Na: 4.1766e-06 |
|  |  |  |  |  | Bl vs Tr: 3.5984e-12 |  |  |  | Bl vs Tr: 7.9563e-12 |
|  |  |  |  |  | Bl vs Ut: 8.3372e-05 |  |  |  | Bl vs Ut: 1.5311e-03 |
|  |  |  |  |  | Tr vs Ut: 6.2643e-04 |  |  |  | Na vs Tr: 4.1158e-02 |
|  |  |  |  |  |  |  |  |  | Tr vs Ut: 4.7434e-05 |
|  | 50 | 7.96E-10 | 45.31 | 3 | Bl vs Na: 8.2402e-06 | 5.62E-11 | 50.72 | 3 | Bl vs Na: 8.5389e-07 |
|  |  |  |  |  | Bl vs Tr: 1.9344e-09 |  |  |  | Bl vs Tr: 1.3496e-10 |
|  |  |  |  |  | Bl vs Ut: 6.2895e-03 |  |  |  | Bl vs Ut: 1.4748e-03 |
|  |  |  |  |  | Tr vs Ut: 9.3860e-05 |  |  |  | Na vs Ut: 2.9203e-02 |
|  |  |  |  |  |  |  |  |  | Tr vs Ut: 8.5504e-05 |
|  | 60 | 0.0009078 | 16.47 | 3 | Bl vs Na: 0.0279 | 2.85E-05 | 23.73 | 3 | Bl vs Na: 2.0767e-03 |
|  |  |  |  |  | Bl vs Tr: 0.0007 |  |  |  | Bl vs Tr: 1.8713e-05 |
|  |  |  |  |  | Tr vs Ut: 0.0473 |  |  |  | Bl vs Ut: 1.7150e-02 |
|  |  |  |  |  |  |  |  |  | Tr vs Ut: 1.4338e-02 |
|  | 70 | 0.0073 | 12.01 | 3 | Bl vs Tr: 0.0047 | 0.007757 | 11.89 | 3 | Bl vs Na: 0.0340 |
|  |  |  |  |  |  |  |  |  | Bl vs Tr: 0.0037 |
|  |  |  |  |  |  |  |  |  | Bl vs Ut: 0.0291 |

KW – Kruskal-Wallis test

**Table S3.** Statistical details of structural analysis of Arc and DARPP-32 (active) colabeled neurons.

| **Area** | **Neuron Morphometry parameters** | **Kruskal-Wallis P value** | **χ2 value** | **df** | **Post hoc P value (Dunn’s test)** |
| --- | --- | --- | --- | --- | --- |
| mNCL | Nodes | 2.52E-02 | 7.36 | 2 | Na vs Tr: 0.0271 |
| lNCL | Neurite length | 6.52E-05 | 19.275 | 2 | Na vs Tr: 2.4718e-02 |
|  |  |  |  |  | Tr vs Ut: 3.4199e-05 |
|  | Nodes | 1.15E-02 | 8.9323 | 2 | Na vs Tr: 0.0231 |
|  |  |  |  |  | Tr vs Ut: 0.0191 |
|  | Cell body area | 2.49E-02 | 7.3861 | 2 | Na vs Ut: 0.0464 |
|  | Neurite field area | 5.88E-06 | 24.089 | 2 | Na vs Tr: 3.2e-02 |
|  |  |  |  |  | Tr vs Ut: 3.3784e-06 |
|  |  |  |  |  | Na vs Ut: 2.3523e-02 |

**Table S4.** Statistical details of Sholl analysis of Arc and DARPP-32 (active) colabeled neurons.

| **Area** | **Radius (µm)** | **Intersections (P values)** | **χ2 value** | **df** | **Intersections (Dunn’s P value)** | **Neurite length (P values)** | **χ2 value** | **df** | **Neurite length (Dunn’s/Tukey’s test P value)** |
| --- | --- | --- | --- | --- | --- | --- | --- | --- | --- |
| lNCL | 30 | 0.0003 | 16.12 | 2 | Tr vs Ut: 0.0001 | 0.0091 | 5.05 | 2, 67 | Tr vs Ut: 0.0066 |
|  | 40 | 0.001 | 13.68 | 2 | Tr vs Ut: 0.0008 | 0.000711 | 14.50 | 2 | Tr vs Ut: 0.0004 |
|  | 50 | 0.0001 | 17.31 | 2 | Tr vs Ut: 9.8013e-05 | 0.0002046 | 16.99 | 2 | Tr vs Ut: 0.0001 |
|  | 60 | 0.0186 | 7.96 | 2 | Na vs Tr: 0.0466 | 0.003978 | 11.05 | 2 | Na vs Tr: 0.0220 |
|  |  |  |  |  | Tr vs Ut: 0.0383 |  |  |  | Tr vs Ut: 0.0074 |

**Table S5.** Statistical details of structural analysis of inactive DARPP-32 neurons in NCL.

| **Area** | **Neuron Morphometry parameters** | **Kruskal-Wallis/ ANOVA P value** | **χ2 / F value** | **df** | **Post hoc P value (Dunn’s)** |
| --- | --- | --- | --- | --- | --- |
| mNCL | Neurite length | 6.14E-09 | 37.816 | 2 | Na vs Ut: 5.126717e-07 |
|  |  |  |  |  | Tr vs Ut: 8.460955e-07 |
|  | Endings | 2.68E-11 | 35.78 | 2, 67 | Na vs Ut: <1e-05 |
|  |  |  |  |  | Tr vs Ut: <1e-05 |
|  | Nodes | 2.74E-08 | 34.827 | 2 | Na vs Ut: 9.844824e-07 |
|  |  |  |  |  | Tr vs Ut: 1.894894e-06 |
|  | Cell body area | 4.83E-05 | 11.57 | 2 | Na vs Ut: <1e-04 |
|  |  |  |  |  | Tr vs Na: 0.0075 |
|  | Neurite field area | 1.67E-08 | 35.812 | 2 | Na vs Ut: 9.844824e-07 |
|  |  |  |  |  | Tr vs Ut: 1.894894e-06 |
| lNCL | Neurite length | 1.85E-09 | 40.213 | 2 | Na vs Ut: 4.548690e-08 |
|  |  |  |  |  | Tr vs Ut: 2.066172e-06 |
|  | Endings | 2.42E-08 | 35.075 | 2 | Na vs Ut: 4.414069e-07 |
|  |  |  |  |  | Tr vs Ut: 8.300441e-06 |
|  | Nodes | 1.17E-08 | 36.526 | 2 | Na vs Ut: 2.144890e-07 |
|  |  |  |  |  | Tr vs Ut: 9.387586e-07 |
|  | Cell body area | 6.69E-04 | 54.004 | 2, 67 | Na vs Ut: 0.00924 |
|  |  |  |  |  | Tr vs Ut: 0.00148 |
|  | Neurite field area | 3.53E-09 | 38.922 | 2 | Na vs Ut: 2.144890e-07 |
|  |  |  |  |  | Tr vs Ut: 9.387586e-07 |

**Table S6.** Statistical details of Sholl analysis of inactive DARPP-32 neurons in NCL.

| **Area** | **Radius (µm)** | **Intersections (P-Value)** | **χ2 / F value** | **df** | **Intersections (Dunn’s / Tukey’s P value)** | **Neurite length ( P-Value)** | **χ2 / F value** | **df** | **Neurite length (Dunn’s / Tukey’s P value)** |
| --- | --- | --- | --- | --- | --- | --- | --- | --- | --- |
| mNCL | 20 | 3.25E-07 | 18.83 | 2, 67 | Na vs Ut: <1e-05 | 6.91E-06 | 14.26 | 2, 67 | Na vs Ut: <1e-04 |
|  |  |  |  |  | Tr vs Ut: 4.94e-05 |  |  |  | Tr vs Ut: 0.00281 |
|  | 30 | 7.47E-09 | 25.06 | 2, 67 | Na vs Ut: <1e-06 | 3.17E-09 | 26.58 | 2, 67 | Na vs Ut: <1e-05 |
|  |  |  |  |  | Tr vs Ut: <1e-06 |  |  |  | Tr vs Ut: <1e-05 |
|  | 40 | 3.22E-08 | 22.76 | 2, 65 | Na vs Ut: <1e-04 | 2.73E-08 | 23.04 | 2, 65 | Na vs Ut: <1e-05 |
|  |  |  |  |  | Tr vs Ut: <1e-04 |  |  |  | Tr vs Ut: <1e-05 |
|  | 50 | 2.06E-08 | 23.87 | 2, 62 | Na vs Ut: <1e-05 | 3.75E-08 | 22.81 | 2, 62 | Na vs Ut: <1e-04 |
|  |  |  |  |  | Tr vs Ut: <1e-05 |  |  |  | Tr vs Ut: <1e-04 |
|  | 60 | 0.000144 | 10.37 | 2, 57 | Na vs Ut: 0.00147 | 9.90E-06 | 14.2 | 2, 57 | Na vs Ut: 0.00016 |
|  |  |  |  |  | Tr vs Ut: 0.00033 |  |  |  | Tr vs Ut: <1e-04 |
|  | 70 | 0.0374 | 6.5721 | 2 | - | 0.0301 | 3.756 | 2, 51 | Na vs Ut: 0.0429 |
| lNCL | 20 | 0.000152 | 10.06 | 2, 67 | Na vs Ut: 0.00032 | 0.0169 | 4.339 | 2, 67 | Na vs Ut: 0.0147 |
|  |  |  |  |  | Tr vs Ut: 0.00439 |  |  |  |  |
|  | 30 | 2.07E-11 | 36.32 | 2, 67 | Na vs Ut: <1e-05 | 2.67E-07 | 30.27 | 2 | Na vs Ut: 2.9256e-06 |
|  |  |  |  |  | Tr vs Ut: <1e-05 |  |  |  | Tr vs Ut: 4.1204e-05 |
|  | 40 | 5.67E-09 | 37.975 | 2 | Na vs Ut: 3.143e-07 | 1.37E-11 | 37.41 | 2, 66 | Na vs Ut: <1e-05 |
|  |  |  |  |  | Tr vs Ut: 1.0734e-06 |  |  |  | Tr vs Ut: <1e-05 |
|  | 50 | 2.44E-07 | 30.453 | 2 | Na vs Ut: 2.534947e-06 | 1.31E-07 | 31.69 | 2 | Na vs Ut: 1.8189e-06 |
|  |  |  |  |  | Tr vs Ut: 1.666073e-05 |  |  |  | Tr vs Ut: 8.8622e-06 |
|  | 60 | 6.19E-05 | 19.379 | 2 | Na vs Ut: 0.0001 | 3.93E-05 | 20.289 | 2 | Na vs Ut: 7.9315e-05 |
|  |  |  |  |  | Tr vs Ut: 0.0009 |  |  |  | Tr vs Ut: 5.5381e-04 |
|  | 70 | 0.01246 | 8.7698 | 2 | Na vs Ut: 0.0099 | 0.0137 | 8.5801 | 2 | Na vs Ut: 0.0102 |

**Table S7.** Statistical information of comparisons between active and inactive DARPP-32 neurons structural features in NCL.

| **Area** | **Experimental group** | **Parameter** | **Test** | **n** | **df** | **t/W** | **P** |
| --- | --- | --- | --- | --- | --- | --- | --- |
| lNCL | Undertrained | Neurite field area | Wilcoxon rank sum test | 30, 30 |  | 302 | 0.02847 |
|  | Trained | Neurite length | Wilcoxon rank sum test | 20, 20 |  | 73 | 0.00037 |
|  | Trained | Nodes | Wilcoxon rank sum test | 20, 20 |  | 113 | 0.01864 |
|  | Trained | Endings | Wilcoxon rank sum test | 20, 20 |  | 103 | 0.0088 |
|  | Trained | Neurite field area | Wilcoxon rank sum test | 20, 20 |  | 79 | 0.00075 |
|  | No-Association | Neurite length | Wilcoxon rank sum test | 20, 20 |  | 33 | 7.57E-07 |
|  | No-Association | Nodes | Wilcoxon rank sum test | 20, 20 |  | 60.5 | 0.00016 |
|  | No-Association | Endings | Wilcoxon rank sum test | 20, 20 |  | 52 | 6.35E-05 |
|  | No-Association | Neurite field area | Wilcoxon rank sum test | 20, 20 |  | 33 | 7.57E-07 |
| mNCL | Undertrained | Nodes | Wilcoxon rank sum test | 30, 30 |  | 681.5 | 0.0006 |
|  |  | Endings | Wilcoxon rank sum test | 30, 30 |  | 679.5 | 0.00068 |
|  |  | Cell body area | Welch's t test | 30, 30 | 57.007 | 2.2074 | 0.03133 |
|  | Trained | Neurite length | Wilcoxon rank sum test | 20, 20 |  | 95 | 0.00389 |
|  | Trained | Neurite field area | Wilcoxon rank sum test | 20, 20 |  | 104 | 0.00871 |
|  | No-Association | Neurite length | Welch's t test | 20, 20 | 36.602 | -4.5857 | 5.13E-05 |
|  | No-Association | Nodes | Welch's t test | 20, 20 | 37.714 | -3.1189 | 0.00347 |
|  | No-Association | Endings | Welch's t test | 20, 20 | 37.832 | -4.0088 | 2.77E-04 |
|  | No-Association | Neurite field area | Welch's t test | 20, 20 | 36.844 | -3.445 | 1.44E-03 |

**Table S8.** Statistical details of DCX positive neuron numbers across various regions.

| **Area** | **Neuron Morphometry parameters** | **ANOVA P value** | **χ2 value** | **df** | **Post hoc P value (Tukey's)** |
| --- | --- | --- | --- | --- | --- |
| Area X | Spherical | 0.00272 | 7.751 | 3, 14 | Tr vs Bl: 0.00164 |
|  |  |  |  |  | Tr vs Ut: 0.02079 |
| MSt | Spherical | 0.00147 | 8.942 | 3, 14 | Tr vs Bl: 0.00127 |
|  |  |  |  |  | Tr vs Ut: 0.00611 |
| dNC | Spherical | 0.0196 | 4.577 | 3, 14 | Tr vs Bl: 0.0194 |
| iNC | Spherical | 0.0161 | 4.857 | 3, 14 | Tr vs Bl: 0.00951 |
| mNCL | Spherical | 0.0267 | 4.153 | 3, 14 | Tr vs Bl: 0.0248 |
| lNCL | Spherical | 0.0387 | 3.667 | 3, 14 | Tr vs Bl: 0.0304 |
| vNC | Spherical | 0.0175 | 4.738 | 3, 14 | Tr vs Bl: 0.0104 |

**Table S9.** Statistical details of structural analysis of DCX neurons in Area X and MSt.

| **Area** | **Neuron Morphometry parameters** | **Kruskal P value** | **χ2 value** | **df** | **Post hoc P value (Dunn’s)** |
| --- | --- | --- | --- | --- | --- |
| Area X | Neurite length | 1.68E-15 | 71.886 | 3 | Bl vs Na: 2.7614e-13 |
|  |  |  |  |  | Bl vs Tr: 1.0340e-06 |
|  |  |  |  |  | Na vs Tr: 3.3577e-02 |
|  |  |  |  |  | Na vs Ut: 1.0028e-09 |
|  |  |  |  |  | Tr vs Ut: 5.4905e-04 |
|  | Endings | 3.19E-07 | 33.022 | 3 | Bl vs Na: 7.0659e-07 |
|  |  |  |  |  | Bl vs Tr: 2.9107e-02 |
|  |  |  |  |  | Na vs Tr: 2.6922e-02 |
|  |  |  |  |  | Na vs Ut: 1.9751e-05 |
|  | Node | 1.83E-06 | 29.421 | 3 | Bl vs Na: 8.2048e-06 |
|  |  |  |  |  | Na vs Tr: 1.9709e-02 |
|  |  |  |  |  | Na vs Ut: 1.5103e-05 |
|  | Cell body area | 7.19E-07 | 31.346 | 3 | Bl vs Na: 1.1221e-04 |
|  |  |  |  |  | Bl vs Tr: 1.6871e-06 |
|  |  |  |  |  | Bl vs Ut: 7.0123e-05 |
|  | Convex hull area | <2.2e-16 | 96.007 | 3 | Bl vs Na: 8.9660e-18 |
|  |  |  |  |  | Bl vs Tr: 3.0441e-10 |
|  |  |  |  |  | Bl vs Ut: 9.7218e-03 |
|  |  |  |  |  | Na vs Tr: 2.2490e-02 |
|  |  |  |  |  | Na vs Ut: 4.7805e-11 |
|  |  |  |  |  | Tr vs Ut: 4.8279e-05 |
| MSt | Neurite length | <2.2e-16 | 97.362 | 3 | Bl vs Na: 2.5606e-18 |
|  |  |  |  |  | Bl vs Tr: 7.7442e-09 |
|  |  |  |  |  | Bl vs Ut: 1.2189e-02 |
|  |  |  |  |  | Na vs Tr: 6.8511e-03 |
|  |  |  |  |  | Na vs Ut: 1.7258e-12 |
|  |  |  |  |  | Tr vs Ut: 1.4138e-04 |
|  | Endings | 3.16E-11 | 51.892 | 3 | Bl vs Na: 6.4528e-10 |
|  |  |  |  |  | Bl vs Tr: 1.4710e-02 |
|  |  |  |  |  | Na vs Tr: 1.0794e-03 |
|  |  |  |  |  | Na vs Ut: 4.2590e-09 |
|  | Node | 3.73E-10 | 46.856 | 3 | Bl vs Na: 2.2765e-09 |
|  |  |  |  |  | Na vs Tr: 9.5374e-05 |
|  |  |  |  |  | Na vs Ut: 4.9110e-08 |
|  | Cell body area | 5.21E-06 | 27.252 | 3 | Bl vs Na: 1.4980e-03 |
|  |  |  |  |  | Bl vs Tr: 3.4184e-06 |
|  |  |  |  |  | Bl vs U: 7.3460e-04 |
|  | Convex hull area | <2.2e-16 | 104.73 | 3 | Bl vs Na: 2.3367e-20 |
|  |  |  |  |  | Bl vs Tr: 3.0679e-10 |
|  |  |  |  |  | Bl vs Ut: 2.7254e-03 |
|  |  |  |  |  | Na vs Tr: 3.4063e-03 |
|  |  |  |  |  | Na vs Ut: 4.8908e-12 |
|  |  |  |  |  | Tr vs Ut: 2.5958e-04 |

**Table S10**. Statistical details of Sholl analysis of inactive DCX neurons in Area X and MSt.

| **Area** | **Radius (µm)** | **Intersections (P value)** | **χ2** | **df** | **Intersections (Dunn’s P value)** | **Neurite length (P value)** | **χ2** | **df** | **Neurite length (Dunn’s P value)** |  |
| --- | --- | --- | --- | --- | --- | --- | --- | --- | --- | --- |
|  |  |  |  |  |  |  |  |  |  |  |
|  |  |  |  |  |  |  |  |  |  |  |
| Area X | 28 | 1.05E-08 | 40.03 | 3 | Bl vs Na: 8.8285e-08 | 1.62E-06 | 29.67 | 3 | Bl vs Na: 4.7901e-06 |  |
|  |  |  |  |  | Bl vs Tr: 1.2090e-04 |  |  |  | Bl vs Tr: 1.7566e-03 |  |
|  |  |  |  |  | Na vs Ut: 4.5461e-05 |  |  |  | Na vs Ut: 6.1731e-04 |  |
|  |  |  |  |  | Tr vs Ut: 1.5231e-02 |  |  |  |  |  |
|  | 38 | <2.2e-16 | 86.07 | 3 | Bl vs Na: 9.3555e-16 | 2.32E-15 | 71.24 | 3 | Bl vs Na: 7.5432e-13 |  |
|  |  |  |  |  | Bl vs Tr: 2.0132e-09 |  |  |  | Bl vs Tr: 2.5125e-08 |  |
|  |  |  |  |  | Bl vs Ut: 1.8099e-02 |  |  |  | Bl vs Ut: 3.4016e-02 |  |
|  |  |  |  |  | Na vs Tr: 3.9360e-02 |  |  |  | Na vs Ut: 3.2989e-08 |  |
|  |  |  |  |  | Na vs Ut: 5.0444e-10 |  |  |  | Tr vs Ut: 1.6367e-04 |  |
|  |  |  |  |  | Tr vs Ut: 7.3697e-05 |  |  |  |  |  |
|  | 48 | 5.27E-14 | 64.90 | 3 | Bl vs Na: 6.4355e-14 | 2.25E-14 | 66.63 | 3 | BL vs Na: 1.0655e-13 |  |
|  |  |  |  |  | Bl vs Tr: 2.5824e-06 |  |  |  | Bl vs Tr: 4.1978e-07 |  |
|  |  |  |  |  | Bl vs Ut: 5.7402e-03 |  |  |  | Bl vs Ut: 6.4435e-03 |  |
|  |  |  |  |  | Na vs Tr: 3.3033e-03 |  |  |  | Na vs Tr: 1.1140e-02 |  |
|  |  |  |  |  | Na vs Ut: 7.0574e-07 |  |  |  | Na vs Ut: 3.2540e-07 |  |
|  |  |  |  |  | Tr vs Ut: 3.4252e-02 |  |  |  | Tr vs Ut: 1.3019e-02 |  |
|  | 58 | 1.54E-07 | 34.52 | 3 | Bl vs Na: 3.2219e-07 | 7.13E-11 | 50.23 | 3 | Bl vs Na: 1.7291e-10 |  |
|  |  |  |  |  | Bl vs Tr: 2.1425e-03 |  |  |  | Bl vs Tr: 9.2329e-06 |  |
|  |  |  |  |  | Bl vs Ut: 3.7791e-02 |  |  |  | Bl vs Ut: 4.0353e-03 |  |
|  |  |  |  |  | Na vs Tr: 4.3333e-02 |  |  |  | Na vs Tr: 4.4294e-02 |  |
|  |  |  |  |  | Na vs Ut: 2.7473e-04 |  |  |  | Na vs Ut: 2.8619e-05 |  |
|  |  |  |  |  |  |  |  |  | Tr vs Ut: 3.8143e-02 |  |
|  | 68 | 0.01377 | 10.65 | 3 | Bl vs Na: 0.00898 | 0.005703 | 12.56 | 3 | Bl vs Na: 0.00515 |  |
| MSt | 18 | 2.52E-05 | 23.98 | 3 | Bl vs Na: 0.0070 | 0.000686 | 17.06 | 3 | Na vs Ut: 0.0025 |  |
|  |  |  |  |  | Bl vs Tr: 0.0278 |  |  |  | Tr vs Ut: 0.0140 |  |
|  |  |  |  |  | Na vs Ut: 0.0002 |  |  |  |  |  |
|  |  |  |  |  | Tr vs Ut: 0.0028 |  |  |  |  |  |
|  | 28 | <2.2e-16 | 76.56 | 3 | Bl vs Na: 6.7050e-13 | 2.24E-12 | 57.28 | 3 | Bl vs Na: 2.4572e-09 |  |
|  |  |  |  |  | Bl vs Tr: 2.4917e-10 |  |  |  | Bl vs Tr: 4.9921e-07 |  |
|  |  |  |  |  | Bl vs Ut: 1.2603e-02 |  |  |  | Na vs Ut: 7.6749e-07 |  |
|  |  |  |  |  | Na vs Ut: 2.6161e-07 |  |  |  | Tr vs Ut: 6.2201e-05 |  |
|  |  |  |  |  | Tr vs Ut: 1.2414e-05 |  |  |  |  |  |
|  | 38 | <2.2e-16 | 97.32 | 3 | Bl vs Na: 5.1717e-16 | < 2.2e-16 | 103.53 | 3 | Bl vs Na: 2.2520e-17 |  |
|  |  |  |  |  | Bl vs Tr: 5.7017e-13 |  |  |  | Bl vs Tr: 2.9542e-13 |  |
|  |  |  |  |  | Bl vs Ut: 5.0555e-03 |  |  |  | Bl vs Ut: 3.7772e-03 |  |
|  |  |  |  |  | Na vs Ut: 3.7436e-09 |  |  |  | Na vs Ut: 5.0266e-10 |  |
|  |  |  |  |  | Tr vs Ut: 3.9490e-07 |  |  |  | Tr vs Ut: 3.6621e-07 |  |
|  | 48 | < 2.2e-16 | 83.46 | 3 | Bl vs Na: 3.9975e-14 | < 2.2e-16 | 85.99 | 3 | Bl vs Na: 4.7081e-14 |  |
|  |  |  |  |  | Bl vs Tr: 4.8811e-09 |  |  |  | Bl vs Tr: 5.4184e-10 |  |
|  |  |  |  |  | Bl vs Ut: 4.1834e-02 |  |  |  | Bl vs Ut: 3.7562e-02 |  |
|  |  |  |  |  | Na vs Ut: 2.5992e-10 |  |  |  | Na vs Ut: 4.1003e-10 |  |
|  |  |  |  |  | Tr vs Ut: 1.1173e-05 |  |  |  | Tr vs Ut: 1.9306e-06 |  |
|  | 58 | 6.96E-08 | 36.15 | 3 | Bl vs Na: 5.9417e-06 | 1.29E-10 | 49.03 | 3 | Bl vs Na: 2.3068e-07 |  |
|  |  |  |  |  | Bl vs Tr: 4.7789e-03 |  |  |  | Bl vs Tr: 1.3014e-04 |  |
|  |  |  |  |  | Na vs Ut: 5.0157e-06 |  |  |  | Na vs Ut: 1.5210e-07 |  |
|  |  |  |  |  | Tr vs Ut: 1.9323e-02 |  |  |  | Tr vs Ut: 4.1277e-04 |  |
|  | 68 | 0.00021 | 19.50 | 3 | Bl vs Na: 0.0070 | 2.67E-05 | 23.86 | 3 | Bl vs Na: 0.0004 |  |
|  |  |  |  |  | Bl vs Tr: 0.0079 |  |  |  | Bl vs Tr: 0.0004 |  |
|  |  |  |  |  | Na vs Ut: 0.0081 |  |  |  | Na vs Ut: 0.0131 |  |
|  |  |  |  |  | Tr vs Ut: 0.0130 |  |  |  | Tr vs Ut: 0.0148 |  |

**Table S11.** Statistical details of structural analysis of active and inactive DCX neurons in MSt.

| **Cell type** | **Area** | **Neuron Morphometry parameters** | **Kruskal-Wallis P value** | **χ2 value** | **df** | **Post hoc P value (Dunn’s)** |
| --- | --- | --- | --- | --- | --- | --- |
| Active | MSt | Cell body area | 0.0123 | 8.7885 | 2 | Tr vs Ut: 0.0092 |
|  |  |  |  |  |  |  |
| Inactive | MSt | Neurite length | 2.93E-08 | 34.691 | 2 | Na vs Ut: 2.954254e-06 |
|  |  |  |  |  |  | Tr vs Ut: 1.429155e-06 |
|  |  | Endings | 2.67E-08 | 34.88 | 2 | Na vs Ut: 7.2774e-06 |
|  |  |  |  |  |  | Tr vs Ut: 5.6143e-07 |
|  |  | Nodes | 2.35E-08 | 35.13 | 2 | Na vs Ut: 9.8818e-06 |
|  |  |  |  |  |  | Tr vs Ut: 3.6874e-07 |
|  |  | Neurite field area | 3.84E-07 | 29.545 | 2 | Na vs Ut: 1.1762e-05 |
|  |  |  |  |  |  | Tr vs Ut: 1.5009e-05 |

**Table S12.** Statistical details of Sholl analysis of active and inactive DCX neurons in MSt.

| **Cell type** | **Area** | **Radius (µm)** | **df** | **Intersections (Kruskal-Wallis / ANOVA P-Value)** | **Intersections (Dunn’s / Tukey’s P value)** | **Neurite length (Kruskal-Wallis / ANOVA P-Value)** | **χ2/F value** | **df** | **Neurite length (Dunn’s / Tukey’s P value)** |
| --- | --- | --- | --- | --- | --- | --- | --- | --- | --- |
| Active | MSt | 18 |  |  | - | 0.0156 | 4.43 | 2, 67 | Tr vs Ut: 0.0121 |
|  |  |  |  |  |  |  |  |  |  |
| Inactive | MSt | 18 | 2 | 1.25E-04 | Na vs Ut: 0.0006 | 4.21E-04 | 15.54 | 2 | Na vs Ut: 0.0015 |
|  |  |  |  |  | Tr vs Ut: 0.0014 |  |  |  | Tr vs Ut: 0.0038 |
|  |  | 28 | 2, 67 | 5.31E-08 | Na vs Ut: <1e-04 | 4.43E-07 | 18.34 | 2, 67 | Na vs Ut: <1e-04 |
|  |  |  |  |  | Tr vs Ut: <1e-04 |  |  |  | Tr vs Ut: <1e-04 |
|  |  | 38 | 2, 66 | 3.01E-07 | Na vs Ut: <1e-04 | 2.31E-07 | 19.43 | 2, 66 | Na vs Ut: <1e-05 |
|  |  |  |  |  | Tr vs Ut: <1e-04 |  |  |  | Tr vs Ut: <1e-05 |
|  |  | 48 | 2 | 1.11E-06 | Na vs Ut: 0.00002 | 2.34E-07 | 19.49 | 2, 65 | Na vs Ut: <1e-05 |
|  |  |  |  |  | Tr vs Ut: 0.00002 |  |  |  | Tr vs Ut: <1e-05 |
|  |  | 58 | 2 | 3.59E-05 | Na vs Ut: 1.3675e-03 | 1.08E-05 | 13.88 | 2, 61 | Na vs Ut: <1e-04 |
|  |  |  |  |  | Tr vs Ut: 7.7969e-05 |  |  |  | Tr vs Ut: <1e-04 |
|  |  | 68 | 2 | 0.01022 | Na vs Ut: 0.0233 | 0.00433 | 10.88 | 2 | Na vs Ut: 0.0264 |
|  |  |  |  |  | Tr vs Ut: 0.0176 |  |  |  | Tr vs Ut: 0.0047 |

**Table S13.** Statistical information of comparisons between active and inactive DCX neurons structural features in MSt.

| **Area** | **Experimental group** | **Parameter** | **Test** | **n** | **df** | **t/W** | **P** |
| --- | --- | --- | --- | --- | --- | --- | --- |
| MSt | Undertrained | Neurite length | Wilcoxon rank sum test | 30, 30 |  | 270 | 0.0073 |
|  | Undertrained | Endings | Wilcoxon rank sum test | 30, 30 |  | 208.5 | 0.0003 |
|  | Undertrained | Nodes | Wilcoxon rank sum test | 30, 30 |  | 253.5 | 0.0035 |
|  | Undertrained | Neurite field area | Wilcoxon rank sum test | 30, 30 |  | 270 | 0.0073 |
|  | Undertrained | Soma Area | Welch’s t-test | 30, 30 | 56.485 | -3.3243 | 0.0015 |
|  | No-Association | Soma Area | Welch’s t-test | 20, 20 | 35.069 | -2.4415 | 0.01981 |

**Table S14**. Statistical details of structural analysis of DCX neurons in NC subdivisions.

| **Area** | **Neuron Morphometry parameters** | **Kruskal P value** | **χ2 value** | **df** | **Post hoc P value (Dunn’s)** |
| --- | --- | --- | --- | --- | --- |
| dNC | Neurite length | < 2.2e-16 | 87.582 | 3 | Bl vs Na: 2.818817e-12 |
|  |  |  |  |  | Bl vs Tr: 1.343757e-17 |
|  |  |  |  |  | Bl vs Ut: 3.639237e-07 |
|  |  |  |  |  | Na vs Ut: 2.135021e-02 |
|  |  |  |  |  | Tr vs Ut: 6.848278e-05 |
|  | Nodes | 1.54E-11 | 53.358 | 3 | Bl vs Na: 3.565885e-08 |
|  |  |  |  |  | Bl vs Tr: 8.021323e-10 |
|  |  |  |  |  | Bl vs Ut: 4.405485e-03 |
|  |  |  |  |  | Na vs Ut: 3.127850e-03 |
|  |  |  |  |  | Tr vs Ut: 2.820970e-04 |
|  | Neurite field area | 4.46E-15 | 69.912 | 3 | Bl vs Na: 2.485072e-10 |
|  |  |  |  |  | Bl vs Tr: 1.792783e-13 |
|  |  |  |  |  | Bl vs Ut: 1.236318e-04 |
|  |  |  |  |  | Na vs Ut: 3.890183e-03 |
|  |  |  |  |  | Tr vs Ut: 9.620369e-05 |
|  | Soma Area | 0.006497 | 12.275 | 3 | Bl vs Na: 0.00513 |
| iNC | Neurite length | 2.48E-13 | 61.752 | 3 | Bl vs Na: 5.156065e-13 |
|  |  |  |  |  | Bl vs Tr: 2.415872e-08 |
|  |  |  |  |  | Bl vs Ut: 1.381076e-05 |
|  |  |  |  |  | Na vs Ut: 1.241345e-03 |
|  | Nodes | 0.0003052 | 18.769 | 3 | Bl vs Na: 0.00073 |
|  |  |  |  |  | Bl vs Tr: 0.00599 |
|  |  |  |  |  | Na vs Ut: 0.03407 |
|  | Neurite field area | 4.01E-07 | 32.551 | 3 | Bl vs Na: 2.736157e-07 |
|  |  |  |  |  | Bl vs Tr: 7.344751e-03 |
|  |  |  |  |  | Na vs Ut: 2.343574e-04 |
|  | Soma Area | 0.008818 | 11.617 | 3 | Bl vs Na: 0.03804 |
|  |  |  |  |  | Bl vs Tr: 0.01658 |
| mNCL | Neurite length | < 2.2e-16 | 85.203 |  | Bl vs Na: 1.694703e-12 |
|  |  |  |  |  | Bl vs Tr: 9.235544e-17 |
|  |  |  |  |  | Bl vs Ut: 1.454247e-09 |
|  |  |  |  |  | Tr vs Ut: 6.447023e-03 |
|  | Nodes | 3.69E-11 | 51.573 |  | Bl vs Na: 3.886423e-06 |
|  |  |  |  |  | Bl vs Tr: 3.087404e-11 |
|  |  |  |  |  | Bl vs Ut: 1.126038e-03 |
|  |  |  |  |  | Tr vs Ut: 2.497804e-04 |
|  | Neurite field area | 2.12E-14 | 66.75 |  | Bl vs Na: 3.198573e-11 |
|  |  |  |  |  | Bl vs Tr: 2.285996e-12 |
|  |  |  |  |  | Bl vs Ut: 5.475690e-07 |
|  |  |  |  |  | Na vs Ut: 4.802876e-02 |
|  |  |  |  |  | Tr vs Ut: 2.169973e-02 |
| lNCL | Neurite length | < 2.2e-16 | 79.901 |  | Bl vs Na: 5.657415e-14 |
|  |  |  |  |  | Bl vs Tr: 5.144917e-14 |
|  |  |  |  |  | Bl vs Ut: 5.819495e-08 |
|  |  |  |  |  | Na vs Ut: 1.443792e-02 |
|  |  |  |  |  | Tr vs Ut: 9.996455e-03 |
|  | Nodes | 1.28E-08 | 39.632 |  | Bl vs Na: 5.092833e-08 |
|  |  |  |  |  | Bl vs Tr: 3.234468e-06 |
|  |  |  |  |  | Bl vs Ut: 3.641940e-03 |
|  |  |  |  |  | Na vs Ut: 8.334678e-03 |
|  | Neurite field area | 6.48E-07 | 31.561 |  | Bl vs Na: 2.509658e-05 |
|  |  |  |  |  | Bl vs Tr: 7.933987e-06 |
|  |  |  |  |  | Bl vs Ut: 3.975881e-02 |
|  |  |  |  |  | Na vs Ut: 2.268959e-02 |
|  |  |  |  |  | Tr vs Ut: 1.193686e-02 |
| vNC | Neurite length | 1.65E-10 | 48.523 |  | Bl vs Na: 1.883061e-09 |
|  |  |  |  |  | Bl vs Tr: 5.385661e-08 |
|  |  |  |  |  | Bl vs Ut: 3.106734e-04 |
|  |  |  |  |  | Na vs Ut: 2.204467e-02 |
|  | Nodes | 0.0001229 | 20.676 |  | Bl vs Na: 0.00047 |
|  |  |  |  |  | Bl vs Tr: 0.00122 |
|  | Neurite field area | 2.09E-07 | 33.894 |  | Bl vs Na: 4.981936e-07 |
|  |  |  |  |  | Bl vs Tr: 1.541250e-04 |
|  |  |  |  |  | Na vs Ut: 1.986350e-03 |

**Table S15**. Statistical details of Sholl analysis of DCX neurons in NC subdivisions.

| **Area** | **Radius (µm)** | **Intersections (P value)** | **χ2 value** | **df** | **Intersections (Dunn’s P value)** | **Neurite length (P value)** | **χ2 value** | **df** | **Neurite length (Dunn’s P value)** |  |
| --- | --- | --- | --- | --- | --- | --- | --- | --- | --- | --- |
|  |  |  |  |  |  |  |  |  |  |  |
| dNC | 20 | 3.75E-10 | 46.84 | 3 | Bl vs Na: 4.585896e-06 | 1.06E-06 | 30.54 | 3 | Bl vs Na: 9.566860e-06 |  |
|  |  |  |  |  | Bl vs Tr: 2.896733e-10 |  |  |  | Bl vs Tr: 1.423749e-05 |  |
|  |  |  |  |  | Bl vs Ut: 3.080685e-04 |  |  |  | Bl vs Ut: 1.595202e-02 |  |
|  |  |  |  |  | Tr vs Ut: 3.458343e-03 |  |  |  | Tr vs Ut: 4.899398e-02 |  |
|  | 30 | 4.65E-14 | 65.15 | 3 | Bl vs Na: 3.982865e-09 | 9.38E-14 | 63.73 | 3 | Bl vs Na: 9.913265e-09 |  |
|  |  |  |  |  | Bl vs Tr: 2.205398e-13 |  |  |  | Bl vs Tr: 3.282826e-13 |  |
|  |  |  |  |  | Bl vs Ut: 5.332721e-05 |  |  |  | Bl vs Ut: 8.500791e-05 |  |
|  |  |  |  |  | Na vs Ut: 3.440019e-02 |  |  |  | Na vs Ut: 4.012314e-02 |  |
|  |  |  |  |  | Tr vs Ut: 2.231618e-04 |  |  |  | Tr vs Ut: 1.827075e-04 |  |
|  | 40 | 7.78E-08 | 35.92 | 3 | Bl vs Na: 3.883177e-05 | 1.01E-08 | 40.10 | 3 | Bl vs Na: 1.524015e-06 |  |
|  |  |  |  |  | Bl vs Tr: 2.675517e-07 |  |  |  | Bl vs Tr: 6.732655e-08 |  |
|  |  |  |  |  | Bl vs Ut: 2.591807e-02 |  |  |  | Bl vs Ut: 5.165195e-03 |  |
|  |  |  |  |  | Na vs Ut: 2.887066e-02 |  |  |  | Na vs Ut: 1.569242e-02 |  |
|  |  |  |  |  | Tr vs Ut: 1.100249e-03 |  |  |  | Tr vs Ut: 3.238231e-03 |  |
|  | 50 | 1.16E-05 | 25.59 | 3 | Bl vs Na: 0.0001885800 | 7.47E-05 | 21.72 | 3 | Bl vs Na: 0.0008980322 |  |
|  |  |  |  |  | Bl vs Tr: 0.0001131071 |  |  |  | Bl vs Tr: 0.0011681818 |  |
|  |  |  |  |  | Bl vs Ut: 0.0486381887 |  |  |  | Na vs Ut: 0.0169900890 |  |
|  |  |  |  |  | Na vs Ut: 0.0209814017 |  |  |  | Tr vs Ut: 0.0203214057 |  |
|  |  |  |  |  | Tr vs Ut: 0.0146355544 |  |  |  |  |  |
|  | 60 | 4.79E-04 | 17.82 | 3 | Na vs Ut: 0.001744675 | 0.0004694 | 17.86 | 3 | Bl vs Na: 0.018244873 |  |
|  |  |  |  |  | Tr vs Ut: 0.023201548 |  |  |  | Bl vs Tr: 0.028744224 |  |
|  |  |  |  |  |  |  |  |  | Na vs Ut: 0.006209491 |  |
|  |  |  |  |  |  |  |  |  | Tr vs Ut: 0.027372011 |  |
| iNC | 20 | 4.50E-04 | 17.95 | 3 | Bl vs Na: 0.001055695 |  |  |  |  |  |
|  |  |  |  |  | Bl vs Tr: 0.001887158 |  |  |  |  |  |
|  |  |  |  |  | Bl vs Ut: 0.013421908 |  |  |  |  |  |
|  | 30 | 1.82E-11 | 53.02 | 3 | Bl vs Na: 5.958740e-11 | 3.00E-09 | 42.59 | 3 | Bl vs Na: 1.042134e-08 |  |
|  |  |  |  |  | Bl vs Tr: 1.072118e-07 |  |  |  | Bl vs Tr: 1.115683e-06 |  |
|  |  |  |  |  | Bl vs Ut: 3.614554e-06 |  |  |  | Bl vs Ut: 6.845753e-05 |  |
|  |  |  |  |  | Na vs Ut: 3.271787e-02 |  |  |  |  |  |
|  | 40 | 5.58E-09 | 41.33 | 3 | Bl vs Na: 1.330541e-08 | 1.95E-09 | 43.47 | 3 | Bl vs Na: 1.400143e-09 |  |
|  |  |  |  |  | Bl vs Tr: 9.561257e-06 |  |  |  | Bl vs Tr: 3.578969e-06 |  |
|  |  |  |  |  | Bl vs Ut: 9.333206e-03 |  |  |  | Bl vs Ut: 3.338069e-04 |  |
|  |  |  |  |  | Na vs Ut: 7.456280e-04 |  |  |  | Na vs Ut: 5.724331e-03 |  |
|  |  |  |  |  | Tr vs Ut: 4.030937e-02 |  |  |  |  |  |
|  | 50 | 4.59E-07 | 32.27 | 3 | Bl vs Na: 9.550034e-07 | 4.96E-06 | 27.35 | 3 | Bl vs Na: 3.649621e-06 |  |
|  |  |  |  |  | Bl vs Tr: 1.718148e-03 |  |  |  | Bl vs Tr: 2.351212e-03 |  |
|  |  |  |  |  | Na vs Ut: 2.760243e-04 |  |  |  | Bl vs Ut: 4.116088e-02 |  |
|  |  |  |  |  |  |  |  |  | Na vs Ut: 3.561309e-03 |  |
|  | 60 | 1.16E-03 | 15.95 | 3 | Bl vs Na: 0.00769535 | 0.001621 | 15.24 | 3 | Bl vs Na: 0.02387079 |  |
|  |  |  |  |  | Bl vs Tr: 0.01636620 |  |  |  | Bl vs Tr: 0.02537852 |  |
|  |  |  |  |  | Na vs Ut: 0.02812947 |  |  |  | Na vs Ut: 0.02678765 |  |
|  |  |  |  |  |  |  |  |  | Tr vs Ut: 0.04169965 |  |
| mNCL | 20 | 1.73E-08 | 39.00 | 3 | Bl vs Na: 1.983993e-04 | 2.33E-06 | 28.92 | 3 | Bl vs Na: 7.371346e-04 |  |
|  |  |  |  |  | Bl vs Tr: 5.732879e-09 |  |  |  | Bl vs Tr: 1.599728e-06 |  |
|  |  |  |  |  | Bl vs Ut: 2.564593e-04 |  |  |  | Bl vs Ut: 6.170838e-04 |  |
|  |  |  |  |  | Tr vs Ut: 2.059328e-02 |  |  |  |  |  |
|  | 30 | < 2.2e-16 | 79.42 | 3 | Bl vs Na: 5.736234e-10 | 2.77E-13 | 61.53 | 3 | Bl vs Na: 1.139491e-07 |  |
|  |  |  |  |  | Bl vs Tr: 8.720984e-17 |  |  |  | Bl vs Tr: 2.523560e-13 |  |
|  |  |  |  |  | Bl vs Ut: 1.738298e-09 |  |  |  | Bl vs Ut: 2.775307e-07 |  |
|  |  |  |  |  | Tr vs Ut: 5.274188e-03 |  |  |  | Tr vs Ut: 1.096734e-02 |  |
|  | 40 | 1.03E-06 | 30.61 | 3 | Bl vs Na: 3.497607e-05 | 3.33E-09 | 42.38 | 3 | Bl vs Na: 2.830228e-06 |  |
|  |  |  |  |  | Bl vs Tr: 5.245500e-07 |  |  |  | Bl vs Tr: 1.042309e-09 |  |
|  |  |  |  |  | Bl vs Ut: 2.332137e-04 |  |  |  | Bl vs Ut: 3.374997e-05 |  |
|  |  |  |  |  |  |  |  |  | Tr vs Ut: 1.469346e-02 |  |
|  | 50 | 0.003764 | 13.45 | 3 | Bl vs Na: 0.019700334 | 0.007858 | 11.87 | 3 | Bl vs Tr: 0.01385610 |  |
|  |  |  |  |  | Bl vs Tr: 0.009277887 |  |  |  |  |  |
|  | 60 | 0.000101 | 21.07 | 3 | Bl vs Na: 0.025756400 | 0.002494 | 14.33 | 3 | Bl vs Na: 0.04418072 |  |
|  |  |  |  |  | Na vs Ut: 0.000475648 |  |  |  | Na vs Ut: 0.01182431 |  |
|  |  |  |  |  | Tr vs Ut: 0.012925888 |  |  |  |  |  |
| lNCL | 20 | 8.74E-10 | 45.12 | 3 | Bl vs Na: 8.172467e-08 | 0.000165 | 20.05 | 3 | Bl vs Na: 0.0001484976 |  |
|  |  |  |  |  | Bl vs Tr: 1.531016e-08 |  |  |  | Bl vs Tr: 0.0033991177 |  |
|  |  |  |  |  | Bl vs Ut: 2.170761e-04 |  |  |  | Bl vs Ut: 0.0145200127 |  |
|  |  |  |  |  | Tr vs Ut: 3.828554e-02 |  |  |  |  |  |
|  | 30 | 1.63E-12 | 57.92 | 3 | Bl vs Na: 6.292596e-11 | 6.82E-14 | 64.38 | 3 | Bl vs Na: 1.654101e-11 |  |
|  |  |  |  |  | Bl vs Tr: 9.879320e-10 |  |  |  | Bl vs Tr: 2.989246e-11 |  |
|  |  |  |  |  | Bl vs Ut: 2.183683e-05 |  |  |  | Bl vs Ut: 1.742387e-05 |  |
|  |  |  |  |  | Na vs Ut: 1.052563e-02 |  |  |  | Na vs Ut: 6.190890e-03 |  |
|  |  |  |  |  | Tr vs Ut: 2.949063e-02 |  |  |  | Tr vs Ut: 6.144994e-03 |  |
|  | 40 | 1.57E-10 | 48.63 | 3 | Bl vs Na: 2.018935e-09 | 1.63E-12 | 57.93 | 3 | Bl vs Na: 5.194983e-11 |  |
|  |  |  |  |  | Bl vs Tr: 1.365312e-08 |  |  |  | Bl vs Tr: 3.559509e-10 |  |
|  |  |  |  |  | Bl vs Ut: 1.581720e-05 |  |  |  | Bl vs Ut: 7.348038e-07 |  |
|  | 50 | 8.11E-06 | 26.34 | 3 | Bl vs Na: 1.037431e-05 | 7.48E-06 | 26.50 | 3 | Bl vs Na: 1.293657e-05 |  |
|  |  |  |  |  | Bl vs Tr: 7.554106e-05 |  |  |  | Bl vs Tr: 2.622548e-05 |  |
|  |  |  |  |  | Bl vs Ut: 7.881417e-03 |  |  |  | Bl vs Ut: 2.904527e-03 |  |
|  | 60 | 2.51E-05 | 23.99 | 3 | Bl vs Na: 5.555223e-05 | 0.000165 | 20.05 | 3 | Bl vs Na: 0.0001119339 |  |
|  |  |  |  |  | Bl vs Tr: 3.376646e-04 |  |  |  | Bl vs Tr: 0.0006402055 |  |
|  |  |  |  |  | Bl vs Ut: 2.254855e-02 |  |  |  | Bl vs Ut: 0.0104985715 |  |
|  |  |  |  |  | Na vs Ut: 2.462277e-02 |  |  |  |  |  |
|  | 70 |  |  |  |  | 0.04398 | 6.25 | 3 | Na vs Ut: 0.04461682 |  |
| vNC | 20 | 0.02899 | 9.02 | 3 |  |  |  |  |  |  |
|  | 30 | 1.74E-10 | 48.41 | 3 | Bl vs Na: 2.563286e-09 | 7.02E-07 | 31.39 | 3 | Bl vs Na: 4.738316e-06 |  |
|  |  |  |  |  | Bl vs Tr: 5.481956e-08 |  |  |  | Bl vs Tr: 2.292456e-05 |  |
|  |  |  |  |  | Bl vs Ut: 5.849791e-04 |  |  |  | Bl vs Ut: 2.257601e-02 |  |
|  |  |  |  |  | Na vs Ut: 7.121111e-03 |  |  |  | Na vs Ut: 2.478406e-02 |  |
|  |  |  |  |  | Tr vs Ut: 2.596918e-02 |  |  |  | Tr vs Ut: 3.610709e-02 |  |
|  | 40 | 6.74E-11 | 50.35 | 3 | Bl vs Na: 5.691402e-09 | 3.56E-11 | 51.65 | 3 | Bl vs Na: 1.035833e-09 |  |
|  |  |  |  |  | Bl vs Tr: 7.380146e-09 |  |  |  | Bl vs Tr: 8.772967e-09 |  |
|  |  |  |  |  | Bl vs Ut: 1.734934e-03 |  |  |  | Bl vs Ut: 4.253376e-04 |  |
|  |  |  |  |  | Na vs Ut: 4.969408e-03 |  |  |  | Na vs Ut: 6.351867e-03 |  |
|  |  |  |  |  | Tr vs Ut: 3.984141e-03 |  |  |  | Tr vs Ut: 1.395540e-02 |  |
|  | 50 | 4.08E-06 | 27.76 | 3 | Bl vs Na: 2.911524e-06 | 2.00E-07 | 33.98 | 3 | Bl vs Na: 3.664008e-07 |  |
|  |  |  |  |  | Bl vs Tr: 3.884803e-04 |  |  |  | Bl vs Tr: 5.972800e-06 |  |
|  |  |  |  |  | Bl vs Ut: 1.791795e-02 |  |  |  | Bl vs Ut: 2.984771e-03 |  |
|  |  |  |  |  | Na vs Ut: 1.829421e-02 |  |  |  | Na vs Ut: 3.006880e-02 |  |
|  | 60 | 0.00048 | 17.82 | 3 | Bl vs Na: 0.001010108 | 0.000321 | 18.66 | 3 | Bl vs Na: 0.0004884742 |  |
|  |  |  |  |  | Bl vs Tr: 0.001831081 |  |  |  | Bl vs Tr: 0.0015469228 |  |
|  | 70 |  |  |  |  | 0.0242 | 9.42 | 3 | Na vs Ut: 0.01942243 |  |

**Table S16**. Statistical details of structural analysis of inactive DCX neurons in NCL subdivisions.

| **Area** | **Parameters** | **Kruskal-Wallis P value** | **χ2 value** | **df** | **Post hoc P value (Dunn’s)** |
| --- | --- | --- | --- | --- | --- |
| mNCL | Neurite length | 3.27E-05 | 20.659 | 2 | Na vs Ut: 0.0006 |
|  |  |  |  |  | Tr vs Ut: 0.0002 |
|  | Endings | 0.0027 | 11.787 | 2 | Na vs Ut: 0.0241 |
|  |  |  |  |  | Tr vs Ut: 0.0048 |
|  | Nodes | 0.0007 | 14.368 | 2 | Na vs Ut: 0.0051 |
|  |  |  |  |  | Tr vs Ut: 0.0027 |
|  | Neurite field area | 0.0002 | 16.475 | 2 | Na vs Ut: 0.0019 |
|  |  |  |  |  | Tr vs Ut: 0.0014 |
| lNCL | Neurite length | 6.06E-06 | 24.029 | 2 | Na vs Ut: 1.8124e-05 |
|  |  |  |  |  | Tr vs Ut: 8.0732e-04 |
|  | Endings | 2.21E-06 | 26.042 | 2 | Na vs Ut: 5.4507e-06 |
|  |  |  |  |  | Tr vs Ut: 7.2868e-04 |
|  | Nodes | 1.30E-06 | 27.102 | 2 | Na vs Ut: 2.9842e-06 |
|  |  |  |  |  | Tr vs Ut: 6.6800e-04 |
|  | Neurite field area | 1.54E-06 | 26.766 | 2 | Na vs Ut: 4.1588e-06 |
|  |  |  |  |  | Tr vs Ut: 2.7428e-04 |

**Table S17**. Statistical details of Sholl analysis of inactive DCX neurons in NCL subdivisions.

| **Area** | **Radius (µm)** | **Intersections (P-Value)** | **χ2 /F value** | **df** | **Intersections (Dunn’s / Tukey’s P value)** | **Neurite length (P-Value)** | **χ2 /F value** | **df** | **Neurite length (Dunn’s / Tukey’s P value)** |
| --- | --- | --- | --- | --- | --- | --- | --- | --- | --- |
| lNCL | 20 | 0.00461 | 5.84 | 2, 67 | Na vs Ut: 0.0102 | - |  |  | - |
|  |  |  |  |  | Tr vs Ut: 0.0244 |  |  |  |  |
|  | 30 | 1.52E-08 | 23.84 | 2, 67 | Na vs Ut: <1e-04 | 2.55E-05 | 21.15 | 2 | Na vs Ut: 5.94e-05 |
|  |  |  |  |  | Tr vs Ut: <1e-04 |  |  |  | Tr vs Ut: 2.079e-03 |
|  | 40 | 1.79E-08 | 23.55 | 2, 67 | Na vs Ut: <1e-05 | 5.15E-08 | 21.78 | 2, 67 | Na vs Ut: 1.14e-06 |
|  |  |  |  |  | Tr vs Ut: <1e-05 |  |  |  | Tr vs Ut: 1.71e-06 |
|  | 50 | 1.92E-05 | 21.73 | 2 | Na vs Ut: 5.9789e-05 | 1.32E-05 | 22.47 | 2 | Na vs Ut: 4.43e-05 |
|  |  |  |  |  | Tr vs Ut: 9.8375e-04 |  |  |  | Tr vs Ut: 7.56e-04 |
|  | 60 | 0.0004 | 15.64 | 2 | Na vs Ut: 0.0003 | 0.000 | 14.83 | 2 | Na vs Ut: 0.0008 |
|  |  |  |  |  | Tr vs Ut: 0.0229 |  |  |  | Tr vs Ut: 0.0097 |
|  | 70 | - |  |  | - | 0.0191 | 7.92 | 2 | Na vs Ut: 0.0167 |
|  |  |  |  |  |  |  |  |  |  |
| mNCL | 30 | 0.001846 | 12.59 | 2 | Na vs Ut: 0.0172 | 0.01492 | 8.41 | 2 | Tr vs Ut: 0.0123 |
|  |  |  |  |  | Tr vs Ut: 0.0036 |  |  |  |  |
|  | 40 | 4.11E-05 | 20.20 | 2 | Na vs Ut: 0.0008 | 0.00261 | 6.51 | 2, 67 | Na vs Ut: 0.0397 |
|  |  |  |  |  | Tr vs Ut: 0.0002 |  |  |  | Tr vs Ut: 0. 0.0034 |
|  | 50 | 4.10E-05 | 20.20 | 2 | Na vs Ut: 0.0004 | 3.59E-05 | 20.47 | 2 | Na vs Ut: 0.0003 |
|  |  |  |  |  | Tr vs Ut: 0.0003 |  |  |  | Tr vs Ut: 0.0003 |
|  | 60 | 0.0001324 | 17.86 | 2 | Na vs Ut: 0.0014 | 0.001692 | 12.76 | 2 | Na vs Ut: 0.0057 |
|  |  |  |  |  | Tr vs Ut: 0.0005 |  |  |  | Tr vs Ut: 0.0064 |
|  | 70 | 0.00649 | 10.08 | 2 | Na vs Ut: 0.0077 | 0.003973 | 11.06 | 2 | Na vs Ut: 0.0090 |
|  |  |  |  |  | Tr vs Ut: 0.0416 |  |  |  | Tr vs Ut: 0.0121 |

**Table S18**. Statistical details of comparisons between active and inactive DCX neurons in NCL subdivisions.

| **Area** | **Experimental group** | **Parameter** | **Test** | **n** | **df** | **t/W** | **P** |
| --- | --- | --- | --- | --- | --- | --- | --- |
| lNCL | Undertrained | Soma area | Wilcoxon rank sum test | 30, 30 |  | 211 | 2.90E-04 |
|  | Trained | Neurite length | Wilcoxon rank sum test | 20, 20 |  | 280.5 | 0.03042 |
|  | No-Association | Neurite length | Wilcoxon rank sum test | 20, 20 |  | 352 | 1.14E-05 |
|  | No-Association | Endings | Welch’s t-test | 20, 20 | 36.558 | 4.645 | 4.30E-05 |
|  | No-Association | Nodes | Welch’s t-test | 20, 20 | 34.863 | 4.8907 | 2.25E-05 |
|  | No-Association | Neurite field area | Wilcoxon rank sum test | 20, 20 |  | 337 | 0.000104 |
|  | No-Association | Soma area | Welch’s t-test | 20, 20 | 37.187 | -2.1684 | 0.03659 |
| mNCL | Undertrained | Soma area | Wilcoxon rank sum test | 30,30 |  | 140 | 4.74E-06 |
|  | Trained | Neurite length | Welch’s t-test | 20, 20 | 35.141 | 3.4994 | 0.001287 |
|  | Trained | neurite field area | Wilcoxon rank sum test | 20, 20 |  | 350 | 1.57E-05 |
|  | Trained | Soma area | Welch’s t-test | 20, 20 | 37.939 | -3.0798 | 0.003841 |
|  | No-Association | Neurite length | Welch’s t-test | 20, 20 | 35.454 | 5.2081 | 8.26E-06 |
|  | No-Association | Endings | Wilcoxon rank sum test | 20, 20 |  | 284 | 0.02325 |
|  | No-Association | Nodes | Wilcoxon rank sum test | 20, 20 |  | 293 | 0.01194 |
|  | No-Association | Neurite field area | Wilcoxon rank sum test | 20, 20 |  | 372 | 2.64E-07 |
|  | No-Association | Soma area | Welch’s t-test | 20, 20 | 34.91 | -2.8849 | 0.006668 |
